# A review of typical biological activities of glycyrrhetinic acid and its derivatives

**DOI:** 10.1039/d3ra08025k

**Published:** 2024-02-22

**Authors:** Liang Chen, Jingwen Gong, Xu Yong, Youbin Li, Shuojin Wang

**Affiliations:** a Hainan Provincial Key Laboratory for Research and Development of Tropical Herbs, Key Laboratory of Tropical Translational Medicine of Ministry of Education, School of Pharmacy Hainan Medical University No. 3, XueYuan Road, LongHua District Haikou City Hainan Province 571199 China wang.shuojin@hainmc.edu.cn; b Department of Thoracic Surgery, Shanghai Pulmonary Hospital, School of Medicine, Tongji University Shanghai 200433 China xuyong@tongji.edu.cn

## Abstract

Glycyrrhetinic acid, a triterpenoid compound primarily sourced from licorice root, exhibits noteworthy biological attributes, including anti-inflammatory, anti-tumor, antibacterial, antiviral, and antioxidant effects. Despite these commendable effects, its further advancement and application, especially in clinical use, have been hindered by its limited druggability, including challenges such as low solubility and bioavailability. To enhance its biological activity and pharmaceutical efficacy, numerous research studies focus on the structural modification, associated biological activity data, and underlying mechanisms of glycyrrhetinic acid and its derivatives. This review endeavors to systematically compile and organize glycyrrhetinic acid derivatives that have demonstrated outstanding biological activities over the preceding decade, delineating their molecular structures, biological effects, underlying mechanisms, and future prospects for assisting researchers in finding and designing novel glycyrrhetinic acid derivatives, foster the exploration of structure–activity relationships, and aid in the screening of potential candidate compounds.

## Abstract 2

Glycyrrhetinic acid, a triterpenoid compound primarily sourced from licorice root, as well as its derivatives produced through structural modification, exhibit noteworthy biological attributes, including anti-inflammatory, anti-tumor, antibacterial, antiviral, and antioxidant effects.

## Graphical abstract

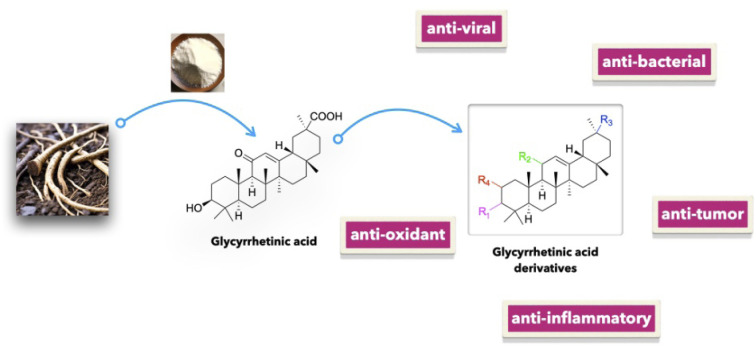

## Introduction

**Fig. 1:**
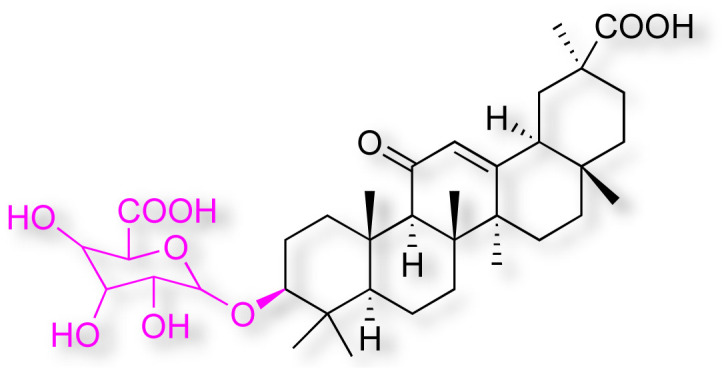
Structure of glycyrrhizic acid.

**Fig. 2:**
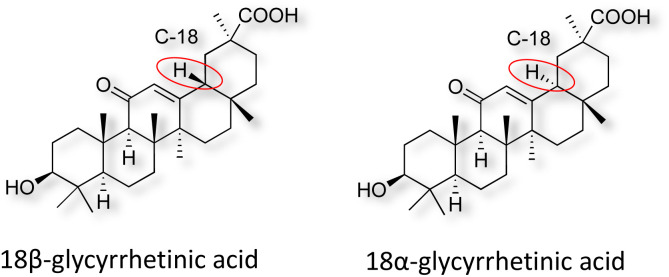
Structure of glycyrrhetinic acid.

Natural products play a crucial role in the exploration of new drugs as they possess broad-spectrum activity against bacteria, fungi, viruses, cancer, and other diseases, and they exhibit a vast array of chemically diverse structures, which hold the potential to serve as lead compounds in drug discovery. In particular, numerous compounds derived from natural products have already exhibited substantial therapeutic potential in the treatment of specific ailments.^1–5^ Among these natural products, glycyrrhetinic acid is the triterpenoid aglycone constituent of glycyrrhizinic acid (Fig. 1), derived from the roots of the licorice plant (*Glycyrrhiza glabra*).^6,7^ There are two isomers of glycyrrhetinic acid (GA), one is (3β,18β)-3-hydroxy-11-oxoolean-12-en-30-oic acid, often called 18β-glycyrrhetinic acid or enoxolone, denoted by 18β-GA. Another one is (3β,18α)-3-hydroxy-11-oxoolean-12-en-29-oic acid, known as 18α-glycyrrhetinic acid, denoted by 18α-GA, as shown in Fig. 2. 18β-GA is the major bioactive constituent of *Glycyrrhiza glabra* and has been investigated to possess a wide range of biological activities, including anti-inflammatory, antitumor, antibacterial, antiviral, and antioxidant. Apart from these characteristic activities, glycyrrhetinic acid has been observed to exhibit additional properties, such as anti-diabetic, anticoagulant, immunoregulatory, anti-cholinesterase, antiarrhythmic, and anti-tetanus toxin actions.^8^

However, 18β-GA's poor druggability, including low solubility and bioavailability, limits its clinical use.^9–12^ To improve the pharmacokinetic properties and enhance the bioactivity, various structural modifications of glycyrrhetinic acid have been carried out to develop novel derivatives for making them attractive candidates for further development as potential drug leads; in the process, extensive studies on the structure–activity relationship (SAR) of 18β-GA and its derivatives have been extensively investigated.^13^ Furthermore, these modifications focused on altering the chemical structure, including the introduction of functional groups, changes in stereochemistry, and modifications of the aglycone skeleton. Studies on the pharmacological activities of 18β-GA derivatives have shown their potential as therapeutics for various diseases, such as inflammatory diseases, cancer, bacterial and viral infections, diabetes, and liver diseases, especially in the past two years.

**Fig. 3:**
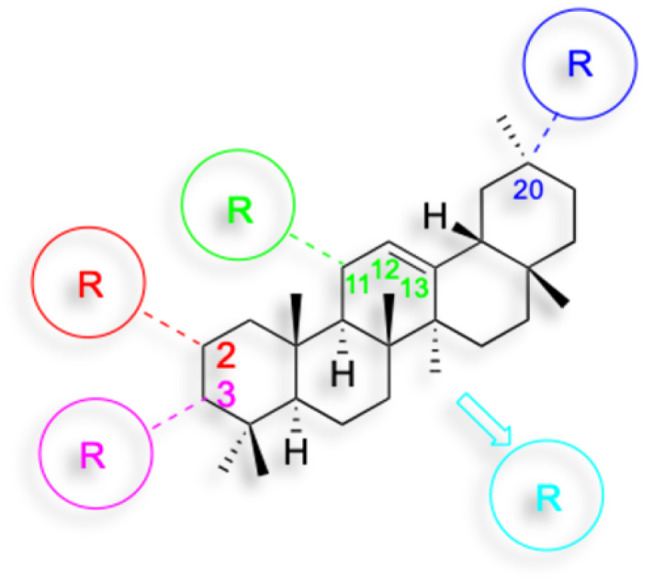
Modification of C-3 sites are labeled in pink, modification of C-2 sites are labeled in red, and modification of C-11 to C-13 sites modification are labeled in fluorescent green. The C-20 carboxyl sites are labeled in blue, while the other sites are labeled in fluorescent blue.

The references incorporated in this review were exclusively sourced from the databases of Google Scholar, PubMed, and Web of Science. The compilation focusing on 18β-GA and its derivatives was based in works published within the temporal span of 2000 to 2023. Significantly, the majority of these citations were published within the most recent half-decade, highlighting the contemporaneity of our curated selection. In addition, we meticulously scrutinized 266 compounds with significant biological activity from a pool of over 500 derivatives sourced from these cited references. To provide a more comprehensive and organized overview, we have compiled tables summarizing the chemical structures and effects or mechanisms of the typical biological activities of 18β-GA and its derivatives, including anti-inflammatory, anti-tumor, antibacterial, antiviral and antioxidant effects. The labeling scheme for the modification sites of all 18β-GA derivatives is described in the form of a diagram. Please refer to Fig. 3 for a visual representation of the labeling scheme.

## Anti-inflammatory activity

Inflammation is considered to be a driver of many diseases, including arteriosclerosis, cancer, autoimmunity, and chronic infections.^14^ The inflammatory process involves multiple cell types, signaling pathways, and molecular mechanisms, leading to adverse reactions such as immunosuppression and gastrointestinal problems.^15–21^ Therefore, the design and optimization of drugs become more complicated. The presence of active ingredients in natural products opens up new opportunities for the development of anti-inflammatory drugs. Extensive research has shown that 18β-GA demonstrates anti-inflammatory effects and holds significant potential as a therapeutic agent for various ailments.^22^ For instance, 18β-GA inhibits the expression of various inflammatory mediators, such as intercellular adhesion molecule-1 (ICAM-1), tumor necrosis factor-alpha (TNF-α), cyclooxygenase-2 (Cox-2), and inducible nitric oxide synthase (iNOS), by inhibiting the activity of the nuclear factor-κB (NF-κB) pathway.^23^ Additionally, 18β-GA has been found to reduce the production of inflammatory cytokines by inhibiting the activity of NF-κB and phosphoinositide 3-kinase (PI3K) and inhibiting the production of NO, prostaglandin E_2_ (PGE_2_), and reactive oxygen species (ROS) under lipopolysaccharide (LPS) stimulation.^24^ However, in an Ana-1 mouse macrophage model, 18β-GA induced the expression of Toll-like receptor 4 and activated the TLR-4 signaling pathway *via* the myeloid differentiation primary response 88 (MYD88) pathway.^25^

In recent years, the research of 18β-GA on anti-inflammation has been deepened. 18β-GA (40 mg kg^−1^ day^−1^) has been found to effectively improve lung function in ovalbumin (OVA)-induced asthma mouse model, reduce lung inflammation and inflammatory cell infiltration, and inhibit the phosphorylation of NF-κB in the treatment of airway allergic inflammation. These effects are achieved through a decrease in the levels of interleukin-5 (IL-5) by approximately 40%, interleukin-13 (IL-13) by approximately 30%, and TNF-α by approximately 70%. Additionally, there is an increase in the levels of nuclear factor erythroid 2-related factor2 (Nrf2) by approximately 50% and heme oxygenase 1 (HO-1) by approximately 50%.^26^ Gupta *et al.* found that 18β-GA has potential therapeutic effects in treating depression. Specifically, it can improve symptoms caused by chronic unpredictable mild stress by activating the brain-derived neurotrophic factor (BDNF)/Tropomyosin receptor kinase B (TrkB) signaling pathway in the prefrontal cortex (PFC) and hippocampus. This activation leads to a reduction in neuroinflammation, liver biomarkers, and stress hormones while increasing the body weight and brain neurotransmitter concentrations.^27^

Additionally, the complex of 18β-GA also exhibits remarkable anti-inflammatory activity. Ishida *et al.* demonstrated that the complex of 18β-GA and hydroxypropyl-β-cyclodextrin can mitigate indomethacin-induced small intestinal injury by reducing TNF-α expression by 27.5%, interleukin-6 (IL-6) by 16.2%, and interleukin-1β (IL-1β) by 17.9% compared to indomethacin-treated tissue.^28^ The salt of 18β-GA and l-arginine can be formed through a co-solvent evaporation reaction, and a solid dispersion called 18β-GA-SD can be created by adding a polymer solvent, Soluplus®, with a hydrophilic-hydrophobic chemical structure. 18β-GA-SD has higher solubility, cell utilization rate, and bioavailability than 18β-GA itself. Following treatment with 18β-GA-SD, enzyme-linked immunosorbent assay (ELISA) analysis revealed an increase in LPS-induced secretion levels of cytokines such as IL-1β, IL-6, macrophage inflammatory protein-1 (MCP-1), TNF-α, interleukin-23 (IL-23), and interleukin-17A (IL-17A) in RAW 264.7 cells; meanwhile, there was a decrease in the levels of interleukins-4 (IL-4) and -10 (IL-10).^11^

In the context of *COVID-19*, 18β-GA has been found to affect the disease by inhibiting the interleukin-17 (IL-17), IL-6, and TNF-α signaling pathways, thereby holding potential as a treatment strategy.^29^ Another study found that a combination of 18β-GA and vitamin C (VC) treatment for *COVID-19* was associated with an increase in immunity and a decrease in inflammatory stress, as well as activation of the T cell receptor signaling pathway, regulation of Fc gamma R-mediated phagocytosis, ErbB signaling pathway, and vascular endothelial growth factor signaling pathway.^30^ Furthermore, highly biocompatible 18β-GA nanoparticles have been synthesized and have shown promise as a treatment strategy for severe acute respiratory syndrome coronavirus 2 (SARS-CoV-2) infections.^31^ Zhou *et al.* demonstrated that 18β-GA inhibited the expression of intercellular adhesion molecule-1 (ICAM-1), TNF-α, COX-2, and iNOS, which was attributed to the inhibition of NF-κB expression and the attenuation of NF-κB nuclear translocation.^32^

**Fig. 4:**
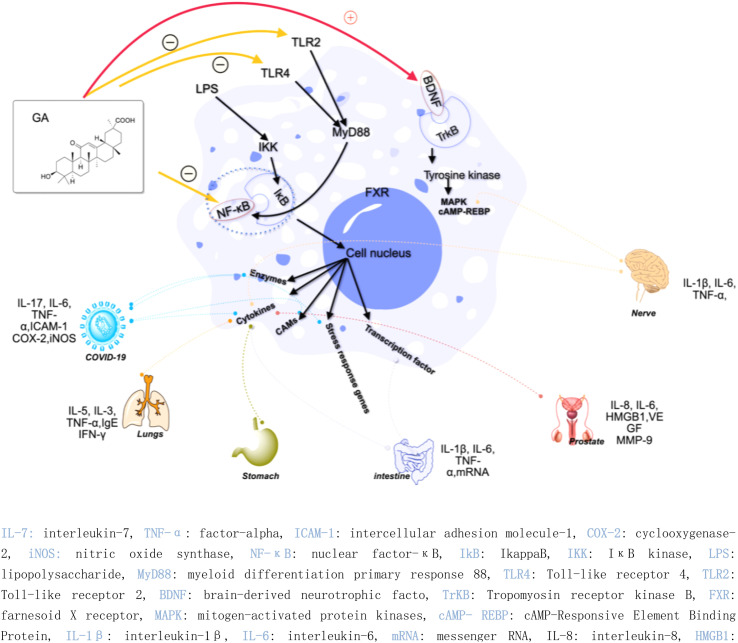
Anti-inflammatory mechanisms of glycyrrhetinic acid and its derivatives.

Moreover, another study discovered that 18α-GA suppressed the invasion on Matrigel-coated transwells of DU145 prostate cancer cells by regulating the expression of nu NF-κB (p65), vascular endothelial growth factor (VEGF), and metalloproteinase-9 (MMP-9). 18α-GA also augmented the expression of non-steroidal anti-inflammatory gene-1 (NAG-1) in DU-145 cells, thereby indicating its capacity for anti-inflammatory activity against prostate cancer cells.^33^ The mechanisms underlying the anti-inflammatory effects of GA discussed above are graphically depicted in Fig. 4. In the realm of hepatoprotective activity, 18β-GA has been shown to mitigate hepatic inflammatory injury caused by hepatitis virus infection by blocking the release of the high mobility group box 1 (HMGB1) cytokine and inhibiting its activity.^34,35^ Furthermore, 18β-GA has potential as a hepatoprotective agent through activating of Nuclear factor erythroid 2-related factor 2 (Nrf2) and peroxisome proliferator-activated receptor gamma (PPAR-γ), and subsequent suppression of NF-κB, and 18β-GA has been shown to protect the liver from cholestatic liver injury induced by lithocholic acid (LCA) by inhibiting the TLR2/NF-κB pathway and upregulating hepatic farnesoid X receptor (FXR) expression, while reducing inflammation and promoting bile excretion. 18β-GA significantly increased the protein levels of the tubular bile acid (BA) efflux transporter bile salt export pump (BSEP) and the basolateral BA efflux transporters multidrug resistance-associated proteins 3 and 4 (MRP3 and MRP4) but decreased the expression of the BA uptake transporter OATP2A1.^23,36–39^ Since the hepatic protection effect of 18β-GA is not only realized through the anti-inflammatory mechanism but could also through the antioxidant mechanism, the review about hepatic protection discussion is in the antioxidant part; Fig. 6 depicts all relevant studies.

In other investigations, various compounds derived from 18β-GA, such as 1–15 (Table 1), have exhibited anti-inflammatory effects. For instance, Ma *et al.* identified three major metabolites (compounds 1–3) produced by the microbial transformation of 18β-GA. These metabolites exhibited potent anti-inflammatory activity by inhibiting LPS-induced NO production in mouse microglia BV2 cells.^40^ The structure and inhibitory activity are shown in Table 1. Another investigation found that compound 4 showed improved pharmacokinetic properties and reduced toxicity in a similar way to fungal metabolism and LPS-induced mouse models.^41^ Li *et al.* found that compound 5 decreased the expression of iNOS, COX-2, and mitogen-activated protein kinases (MAPKs) as well as the activation of NF-κB in LPS-stimulated RAW 264.7 cells.^42^ More recently, Yang *et al.* investigated the anti-inflammatory effects of compound 6 on ear edema in mice and LPS-stimulated RAW 264.7 macrophages, respectively.^43^ Compound 6 was shown to decrease approximately 59.69% of 12-*O*-tetradecanoylphorbol-13-acetate (TPA)-induced ear edema with a gavage treatment of 40.0 mg mL^−1^, and immunohistochemistry results revealed that this effect was related to the inhibition of TPA-induced upregulation of TNF-α. Compound 7 effectively inhibited the protein and mRNA expression of iNOS and the mRNA expression of TNF-α, IL-6, and IL-1β in LPS-stimulated RAW 264.7 macrophages. Bian *et al.* investigated the anti-inflammatory effects of compound 8 on LPS-induced RAW 264.7 cells and found that it suppressed the expression of pro-inflammatory cytokines including IL-6, TNF-α, and NO.^44^ Compounds 9–12 showed significant inhibition activity against NO and IL-6.^45–47^ Among these compounds, compound 12 was identified as the most potent anti-inflammatory agent, exhibiting a significant reduction in inflammatory cytokine levels in the mouse model of AKI by inhibiting TNF-α and IL-6 in a dose-dependent manner. Compound 13 also has anti-inflammatory activity, and studies have shown that it interacts with proteins in the inflammatory process, such as matrix metalloproteinase MMP9, neutrophil elastase, and thrombin.^48^ Tu *et al.* focus on the anti-inflammatory activity of novel 18β-GA derivatives. The study evaluated the derivatives' activity in mouse models of acute inflammation induced by carrageenan. The results showed that several compounds demonstrated significant inhibition of paw edema and leukocyte infiltration.^49^ The results obtained from both *in vitro* and *in vivo* experiments indicate that compound 14 and compound 15 exhibit anti-inflammatory effects by reducing the expression of NO, pro-inflammatory cytokines, and chemokines, such as IL-1β, IL-6, IL-12, TNF-α, MCP-1, and macrophage inflammatory protein-1 alpha (MIP-1α) while increasing the expression of anti-inflammatory cytokine IL-10. Wang *et al.* introduced Soluplus®-glycyrrhetinic acid solid dispersion, which significantly improves the bioavailability and anti-inflammatory activity of 18β-GA. The solubility of 18β-GA increased with the addition of Soluplus®, and the bioavailability was enhanced 2.61-fold. The anti-inflammatory activity of 18β-GA was also improved by 32.3%.^11^ Compounds 16–21 have been structurally modified at the C-2 and C-30 carboxyl positions of 18β-GA. These derivatives of 18β-GA have previously demonstrated outstanding anti-inflammatory activity, as seen in Table 1.^50–52^

**Table 1:** Chemical structure and anti-inflammation activity of glycyrrhetinic acid and its derivatives 1–21

Compounds	18β-GA	1	2	3
Structure	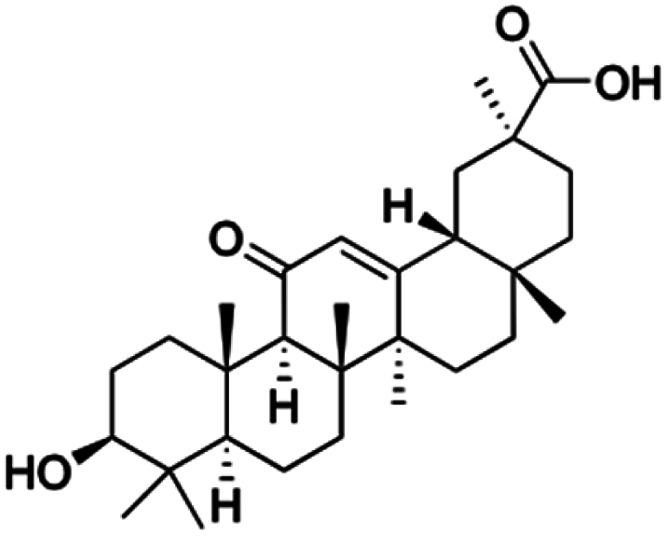	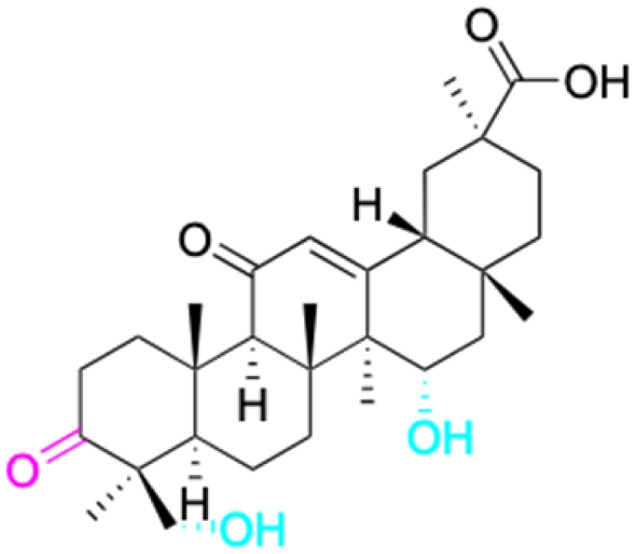	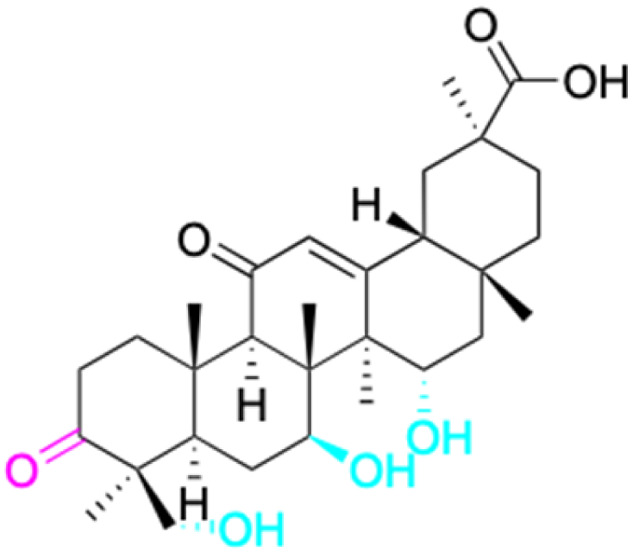	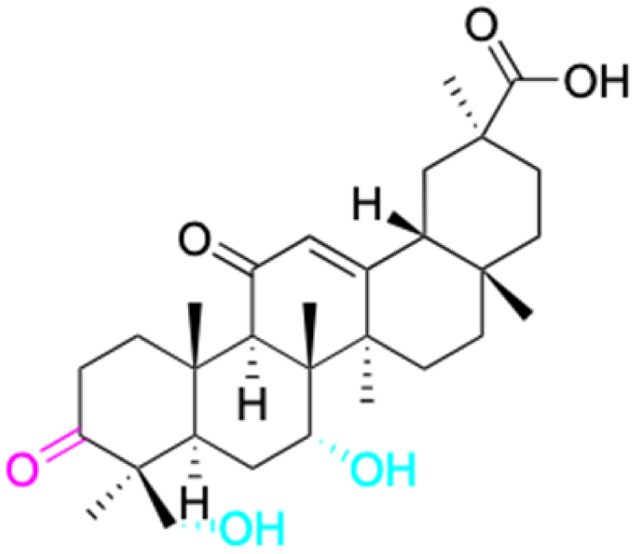
Effects or mechanisms	11 β-HSD1: IC_50_ = 0.778 μM	1:	2:	3:
	11 β-HSD2: IC_50_ = 0.257 μM	NO inhibitory assay in microglia BV2 cells: IC_50_ = 760 μM	NO inhibitory assay in microglia BV2 cells: IC_50_ = 940 μM	NO inhibitory assay in microglia BV2 cells: IC_50_ = 160 μM
Reference	51	40	40	40
Compounds	4		5	
Structure	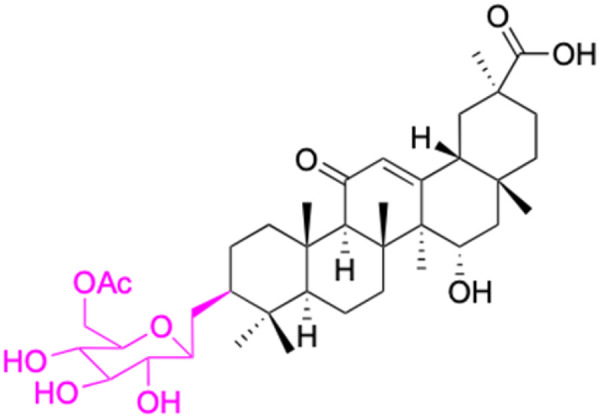		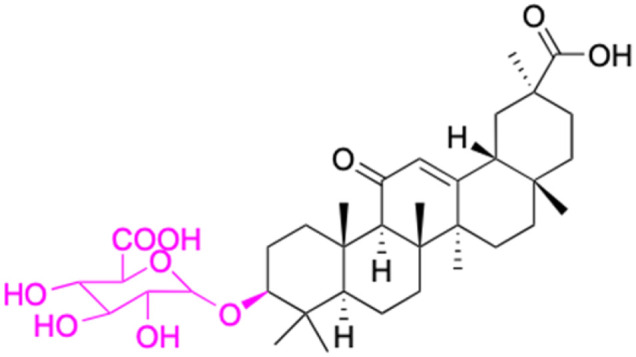	
Effects or mechanisms	4:		5:	
	NO inhibitory assay in RAW 264.7: IC_50_ = 10.13 μM		Inhibited iNOS, COX-2, MAPKs, and NF-κB in the LPS-stimulated RAW 264.7 cells	
Reference	46		42	
Compounds	6	7	8	
Structure	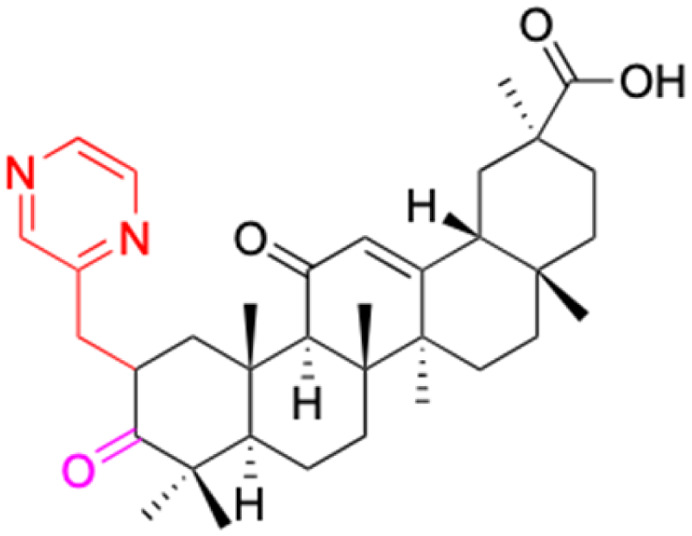	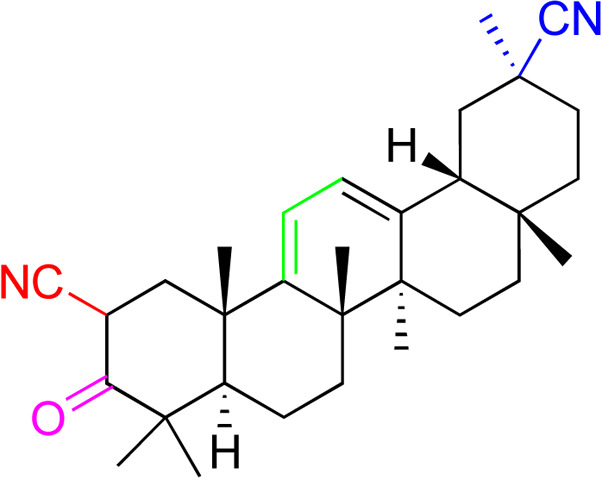	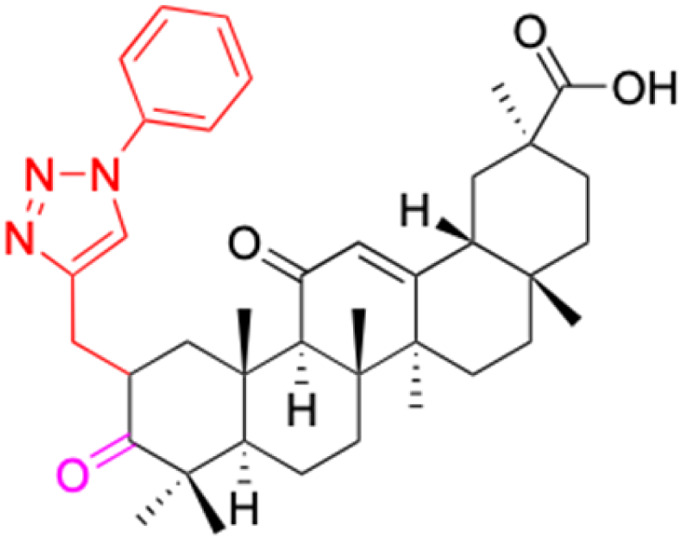	
Effects or mechanisms	6:	7:	8:	
	Delayed TPA-induced (20 mg kg^−1^) overexpression of TNF-α was better than the ibuprofen (40 mg kg^−1^). For IL-1β, at 40 mg kg^−1^ was preferable to ibuprofen at 40 mg kg^−1^	Inhibited LPS-induced NO production. Inhibited iNOS, TNF-α, IL-6, and IL-1β in LPS-stimulated RAW 264.7 macrophages	Inhibited TPA-induced up regulation of the pro-inflammatory cytokines TNF-α and IL-1β and decreased the expression level of p65 in the NF-κB signaling pathway	
		Inhibition at 50 μM: 99.08%		
Reference	43	53	44	
Compounds	9		10	
Structure	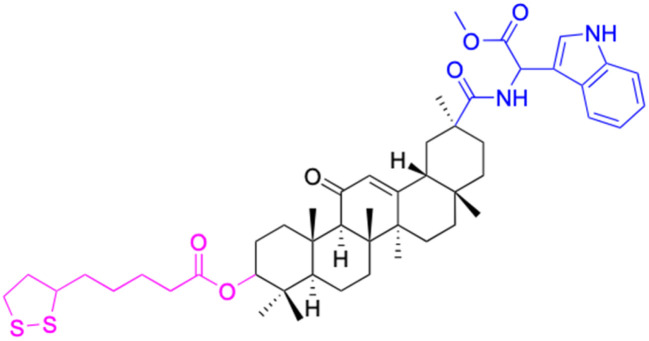		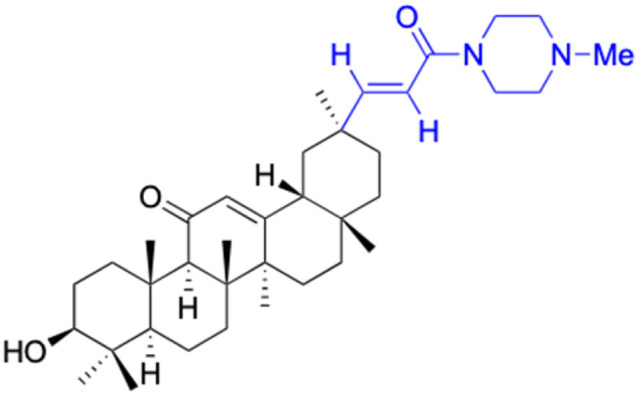	
Effects or mechanisms	9:		10:	
	NO inhibitory assay in RAW 264.7: IC_50_ = 18.5 μM		NO and IL-6 inhibitory activity in RAW 264.7: IC_50_ = 13.3 μM	
Reference	45		46	
Compounds	11		12	
Structure	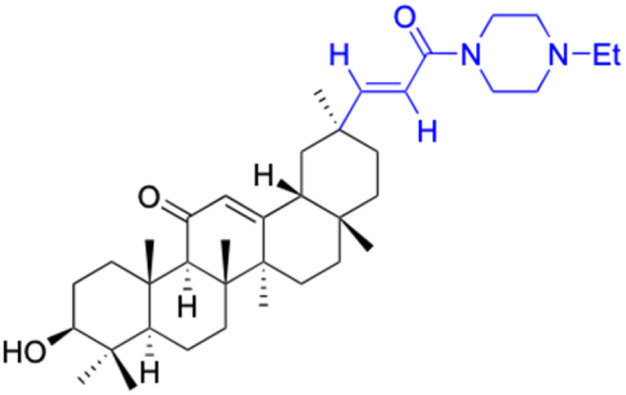		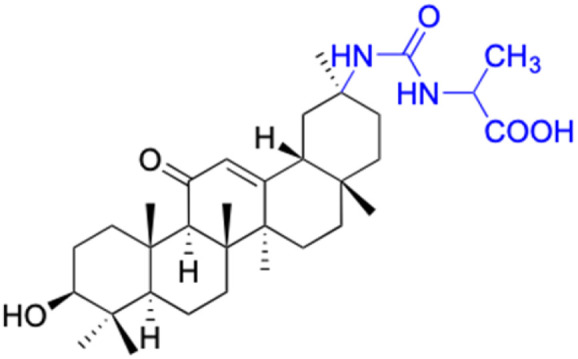	
Effects or mechanisms	11:		12:	
	NO and IL-6 inhibitory activity in RAW 264.7: IC_50_ = 15.5 μM		NO inhibitory assay in RAW 264.7: IC_50_ = 2.04 μM	
Reference	46		47	
Compounds	13		14–15	
Structure	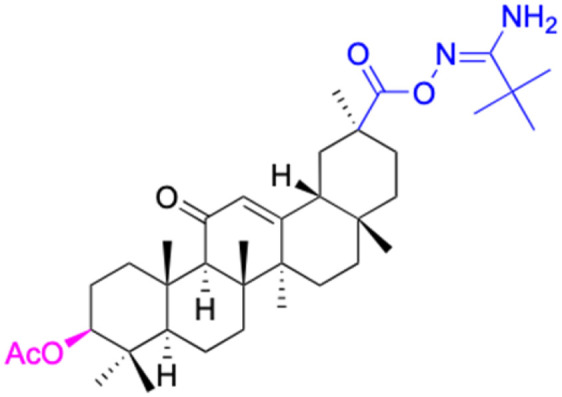		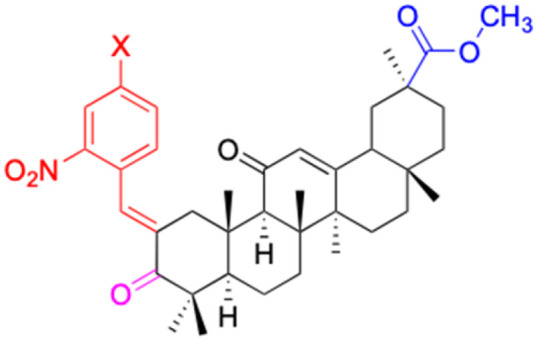	
Effects or mechanisms	13:		14:	
	Inhibit inflammatory response (10–50 μM) induced by IFNγ in macrophages *in vitro* and carrageenan in murine models *in vivo*, probably by primary interactions with active sites of MMP9, neutrophil elastase, and thrombin		X = Cl, IC_50_ = 53.0 μM	
			15:	
			X = F, IC_50_ = 55.4 μM	
			Anti-inflammatory activities through the downregulation of NO, pro-inflammatory cytokines and chemokines (IL-1β, IL-6, IL-12, TNF-α, MCP-1, and MIP-1α) and upregulation of anti-inflammatory cytokines (IL-10). IC_50_ of NO inhibitory assay in microglia BV2 cells	
Reference	48 and 52		49	
Compounds	16		17	
Structure	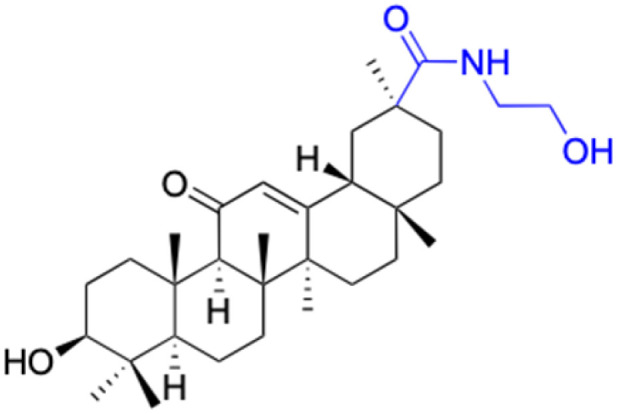		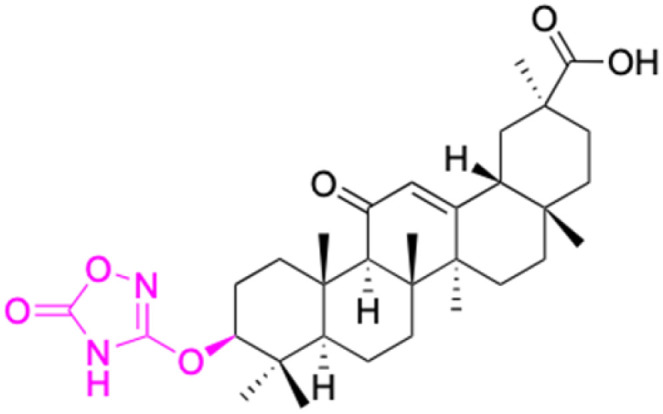	
Effects or mechanisms	16:		17:	
	11β-HSD2: IC_50_ = 0.004 nM		11β-HSD1: IC_50_ = 0.14 μM	
			11β-HSD2: IC_50_ = 0.011 μM	
Reference	50		51	
Compounds	18		19	
Structure	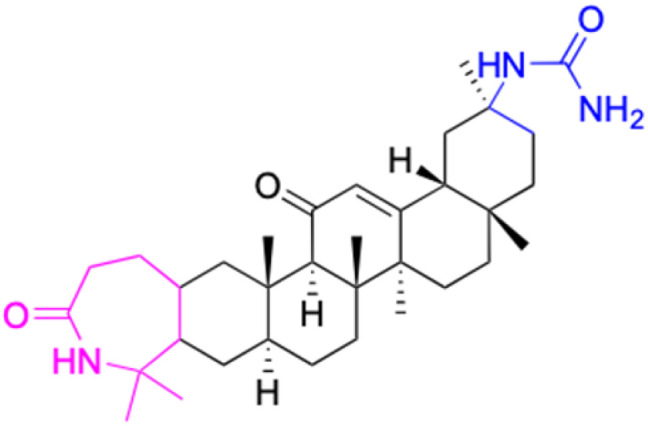		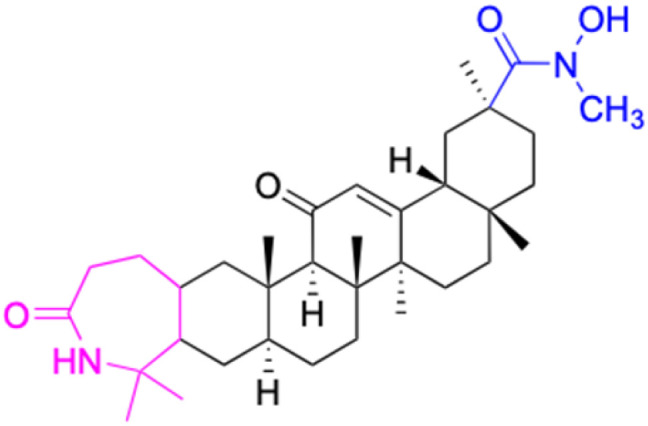	
Effects or mechanisms	18:		19:	
	11β-HSD1: IC_50_ = 45 μM		11β-HSD1: IC_50_ > 40 μM	
	11β-HSD2: IC_50_ = 0.033 μM		11β-HSD2: IC_50_ = 0.011 μM	
Reference	54		54	
Compounds	20		21	
Structure	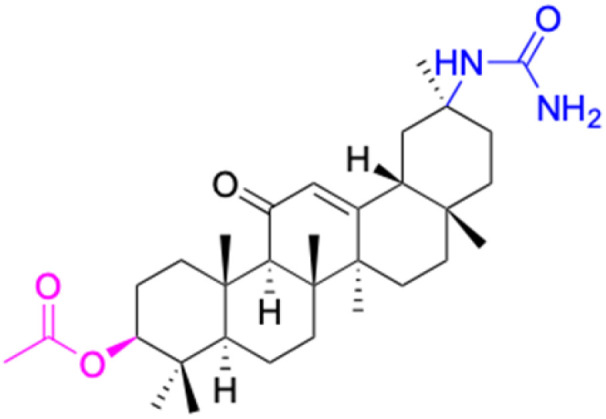		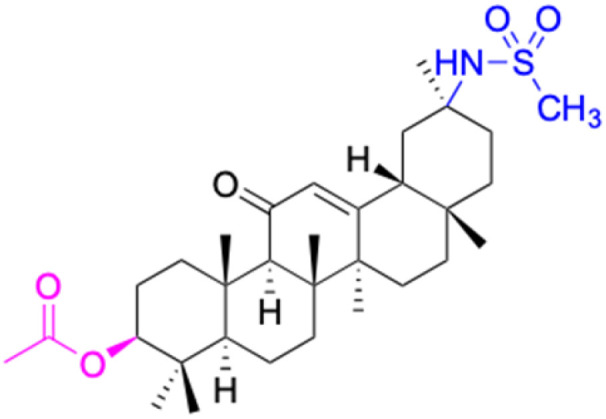	
Effects or mechanisms	20:		21:	
	11β-HSD1: IC_50_ = 8.3 μM		11β-HSD1: IC_50_ > 40 μM	
	11β-HSD2: IC_50_ = 0.104 μM		11β-HSD2: IC_50_ = 0.0069 μM	
Reference	52		52	
Abbreviations	IL-6: the Interleukin-6. NF-κB: nuclear factor kappa-light-chain-enhancer of activated b cells. TNF-α: the tumor necrosis factor. COX-2: cyclooxygenase-2. MAPKs: mitogen-activated protein kinases. MIP-1α: macrophage inflammatory protein-1 alpha. 11β-HSD: 11β-hydroxysteroid dehydrogenase			

In conclusion, 18β-GA has potential therapeutic applications for various conditions due to its anti-inflammatory effects. Although more research is required, the use of 18β-GA and its derivatives may provide new avenues for treating inflammation-related diseases.

## Antitumor activity

Cancer ravages and cripples the earth's inhabitants, ranking among the foremost destroyers of life.^55^ For countless years, scholars have been devoting themselves to the quest for a cure for tumors. Presently, the globe is awash with more than 80 conventional anti-tumor medications, ranging from cytotoxic drugs and hormones, to biological response modifiers (BRMs) and monoclonal antibodies.^56^ The majority of anticancer medications exhibit notable toxicity and necessitate administration in periodic cycles to mitigate adverse effects and impede the emergence of drug resistance. However, the excellent vitality of natural compounds adds new impetus to the research and development of anticancer drugs.^57^ And within this pantheon of treatment options stands the 18β-GA compound—a veritable powerhouse in its ability to vanquish cancerous cells from any part of the human body with unrivaled efficacy. Scores of meticulous studies attest to the fact that this drug is a game-changer in the fight against various forms of cancer. The sterling performance against malignant cells has been proven time and time again, and it holds immense potential as an agent in the battle against cancer. Wang *et al.* demonstrated that 18β-GA has potent inhibitory effects on colorectal cancer cell proliferation *in vitro* and *in vivo*. This study showed that 18β-GA treatment resulted in a significant reduction in cell migration, invasion, and wound healing capability, accompanied by the downregulation of matrix metalloproteinase (MMP) expression. Moreover, 18β-GA decreased the protein levels of phosphorylated PI3K, protein kinase B (AKT), Signal Transducer and Activator of Transcription 3 (STAT3), c-Jun N-terminal Kinase (JNK), p38 mitogen-activated protein kinase (p38), and NF-κB p65, where the phosphorylation of PI3K and STAT3 decreased as early as 2 h after 18β-GA treatment.^58^ Luo *et al.* found that 18β-GA-induced apoptosis and G2/M cell cycle arrest and inhibited migration *via* the ROS/MAPK/STAT3/NF-κB signaling pathways in A549 lung cancer cells. They also found that 18β-GA could reduce tumor growth in a mouse xenograft model. In breast cancer treatment,^59^ Shi *et al.* found that a combination of 18β-GA and doxorubicin enhanced cytotoxicity, apoptosis, and loss of mitochondrial membrane potential *via* the upregulation of a mitochondrial-dependent apoptosis pathway against MCF-7 (breast adenocarcinoma cell line) cells.^60^ In recent years, 18β-GA has also been found to have potential in liver cancer-targeted therapy. Speciale *et al.* provided a comprehensive review of the topic.^61^

The derivatives of 18β-GA have been unearthed to harbor even more potent cancer properties in comparison to the progenitor compound. One of the most remarkable advantages of 18β-GA lies in its all-encompassing efficacy in targeting a myriad of cancer types. It has conspicuously showcased outstanding effectiveness against cancers of the digestive tract, liver, nervous system, reproductive system, immune system, thyroid, and other organ-related cancers. This renders it an invaluable weapon in the war against cancer.^62,63^ The 18β-GA's anti-cancer effects are believed to stem from its capacity to incite apoptosis, a process of purposeful cell death, in cancer cells. Additionally, it also exhibits anti-inflammatory and antioxidant properties that can shield cells from harm and amplify the growth of healthy cells. As demonstrated in Table 2, we have amassed an extensive collection of 18β-GA derivatives with extraordinary anticancer activity.

**Table 2:** Chemical structure and antitumor activity of glycyrrhetinic acid and its derivatives 22–154

Compounds	18β-GA	22–25	26–27	28–33
Structure	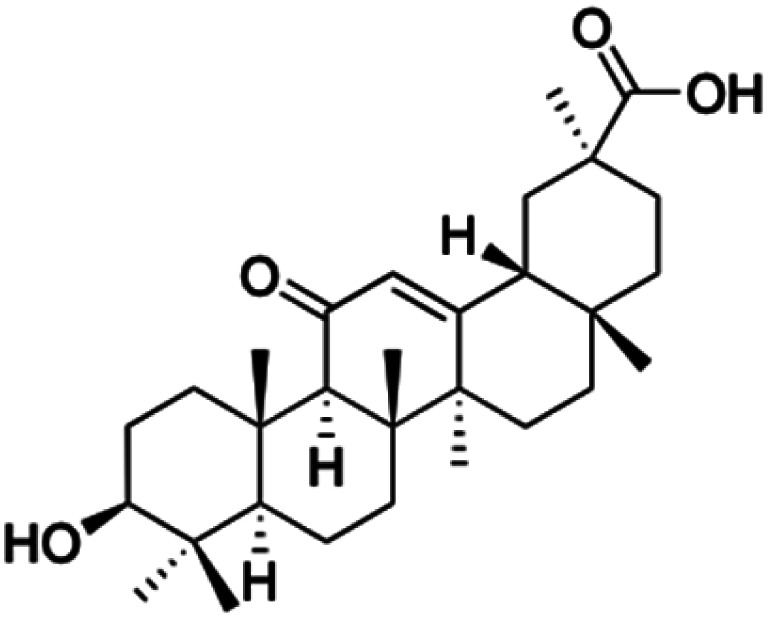	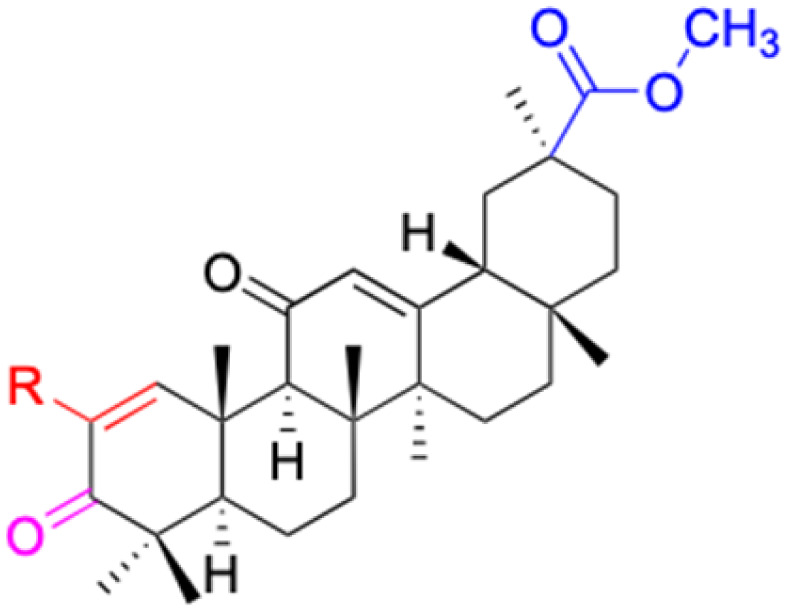	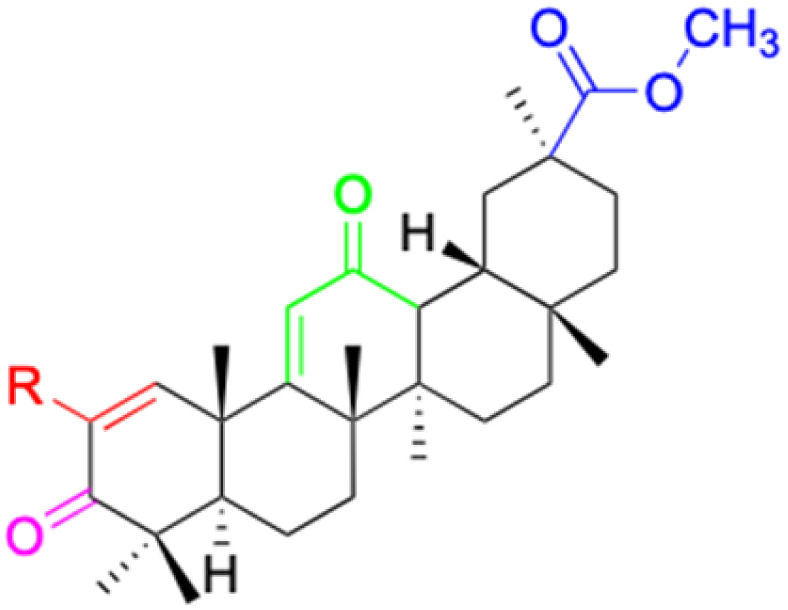	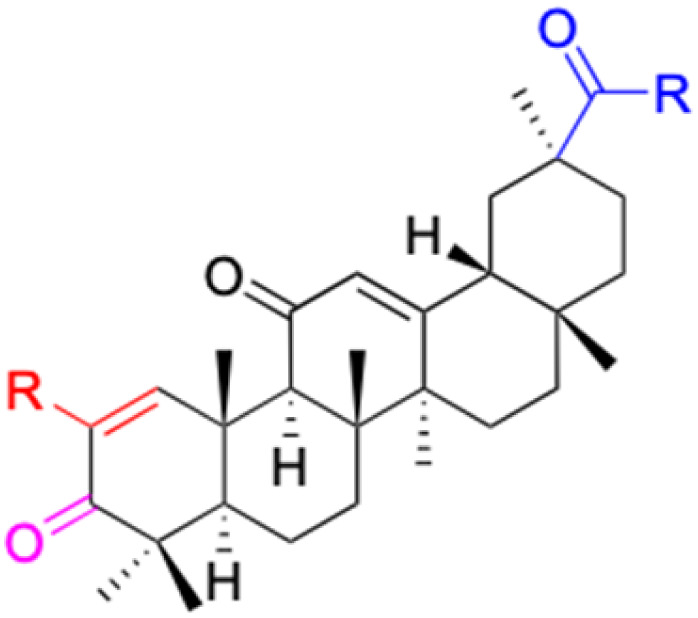
Effects or mechanisms	518A2: IC_50_ = 83.92 μM	22: R = SO_2_CH_3_	26: R = I	28: R = OCH_3_
	8505C: IC_50_ = 86.50 μM	KU7: IC_50_ = 3.3 μM	253JB-V: C_50_ = 3.6 μM	253JB-V: IC_50_ = 0.25 μM
	A253: IC_50_ = 80.78 μM	Panc-1: IC_50_ = 7.6 μM	KU7I: C_50_ = 2.6 μM	KU7: IC_50_ = 1.59 pM
	A2780: IC_50_ = 74.57 μM	Panc-28: IC_50_ = 9.7 μM	Panc-1: IC_50_ = 4.4 μM	Panc-1: IC_50_ = 1.22 μM
	A431: IC_50_ = 79.58 μM	23: R = I	Panc-28: IC_50_ = 3.6 μM	Panc-28: IC_50_ = 1.80 μM
	A549: IC_50_ = 82.76 μM	253JB-V: IC_50_ = 2.6 μM	27: R = CF_3_	29: R = H
	DLD-1: IC_50_ = 81.21 μM	KU7: IC_50_ = 3.0 μM	253JB-V: IC_50_ = 0.3 μM	253JB-V: IC_50_ = 6.10 μM
	FADU: IC_50_ = 84.55 μM	Panc-1: IC_50_ = 4.0 μM	KU7: IC_50_ = 1.3 μM	KU7: IC_50_ = 5.88 μM
	HCT-8: IC_50_ = 78.85 μM	24: R = P=O(OCH_3_)_2_	Panc-1: IC_50_ = 0.68 μM	Panc-1: IC_50_ = 3.81 μM
	HT-29: IC_50_ = 80.09 μM	253JB-V: IC_50_ = 7.9 μM	Panc-28: IC_50_ = 1.1 μM	Panc-28: IC_50_ = 7.32 μM
	LIPO: IC_50_ = 81.44 μM	KU7: IC_50_ = 3.7 μM		30: R = piperidinyl
	MCF-7: IC_50_ = 84.70 μM	Panc-1: IC_50_ = 6.1 μM		HL-60: IC_50_ = 1.4 μM
	SW480: IC_50_ = 86.80 μM	Panc-28: IC_50_ = 8.1 μM		31: R= 1,4-bipiperidinyl
	SW1736: IC_50_ = 76.93 μM	25: R = CF_3_		HL-60: IC_50_ = 0.8 μM
	NIH 3T3: IC_50_ = 18.52 μM	253JB-V: IC_50_ = 0.67 μM		32: R = 4-methylpiperazinyl
	HCT-11: IC_50_ = 78.83 μM	KU7: IC_50_ = 0.38 μM		HL-60: IC_50_ = 1.2 μM
	HCT-116:IC_50_ = 78.83 μM	Panc-1: IC_50_ = 0.82 μM		33: R = piperazinyl
		Panc-28: IC_50_ = 1.1 μM		HL-60: IC_50_ = 1.7 μM
Reference	59–61 and 88	82 and 83	82 and 83	82 and 83
Compounds	34	35–37	38–41	42–43
Structure	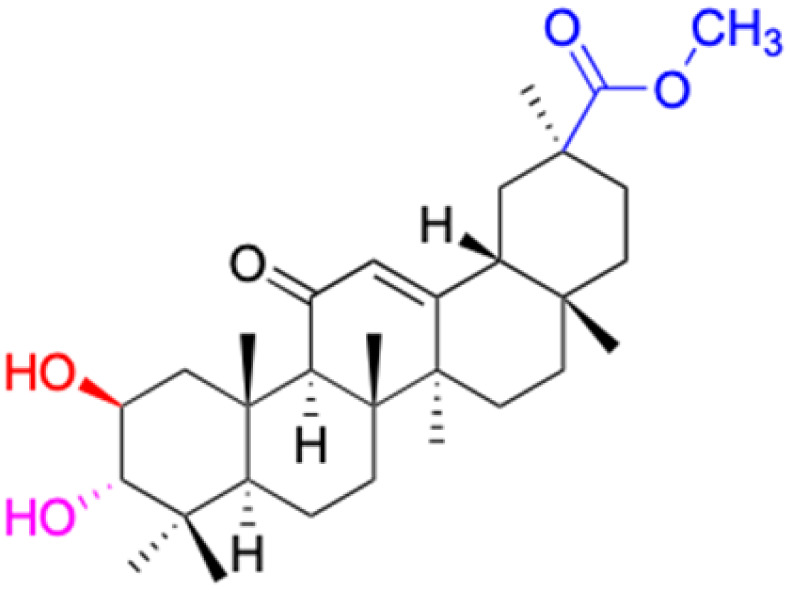	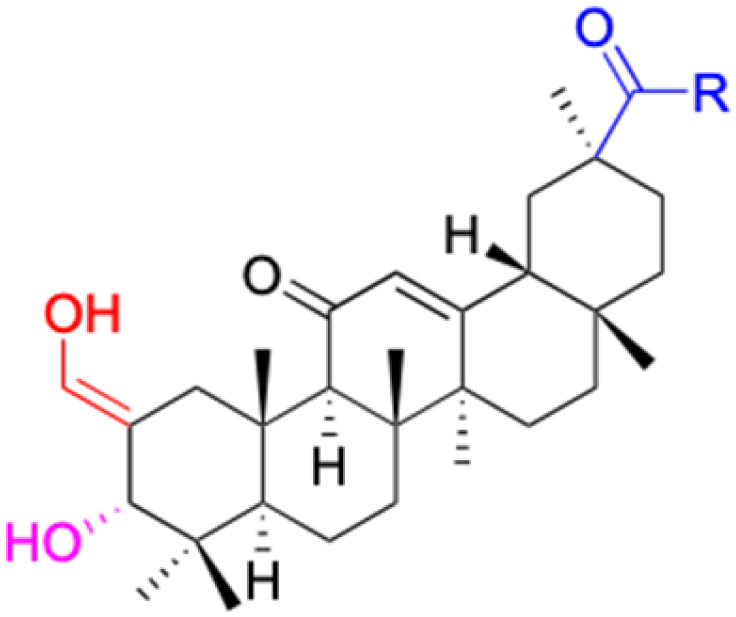	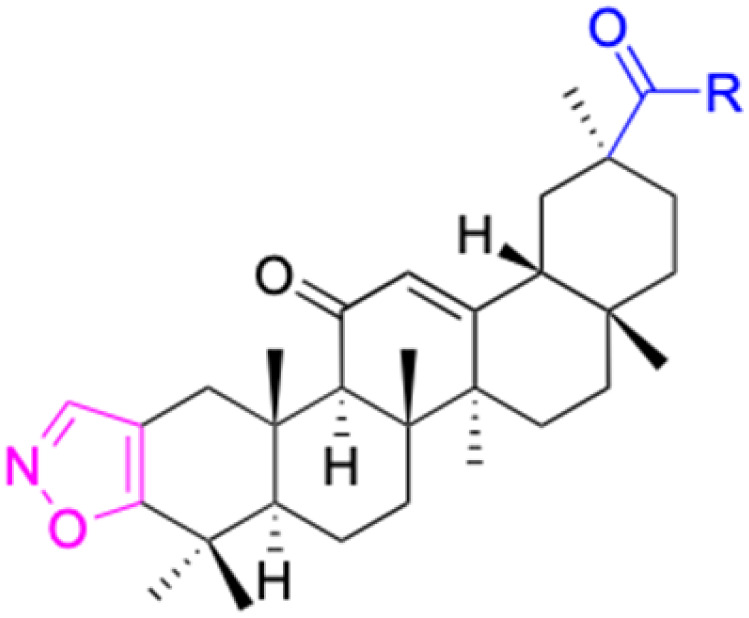	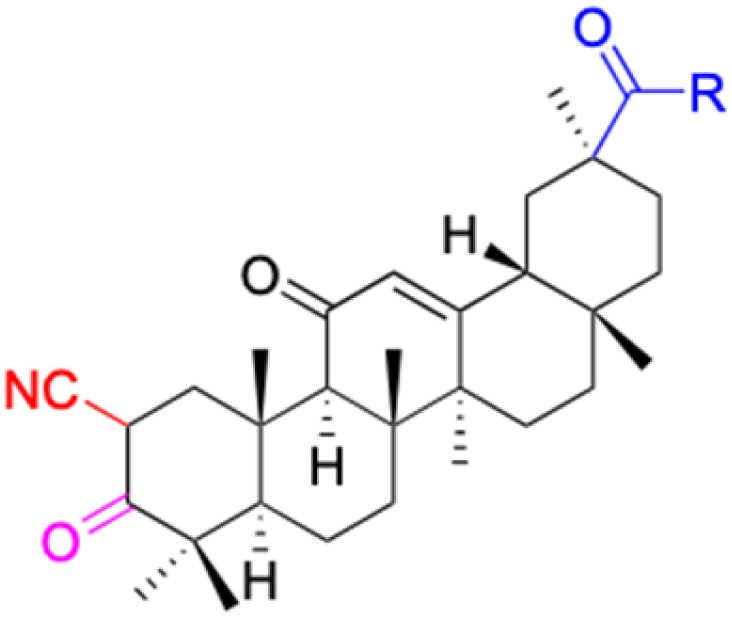
Effects or mechanisms	34:	35: R = piperidinyl	38: R = piperidinyl	42: R = piperidinyl
	HepG-2:	HL-60: IC_50_ = 5.5 μM	HL-60: IC_50_ = 1.7 μM	HL-60: IC_50_ = 8.6 μM
	IC_50_ = 0.22 μM	36: R = 1,4′-bipiperidinyl	39: R = 1,4′-bipiperidinyl	43: R = 1,4′-bipiperidinyl
		HL-60: IC_50_ = 3.3 μM	HL-60: IC_50_ = 7.7 μM	HL-60: IC_50_ = 7.5 μM
		37: R = 4-methylpiperazinyl	40: R = 4-methylpiperazinyl	
		HL-60: IC_50_ = 6.1 μM	HL-60: IC_50_ = 7.9 μM	
			41: R = piperazinyl	
			HL-60: IC_50_ = 8.2 μM	
Reference	65	83	83	83
Compounds	44	45		
Structure	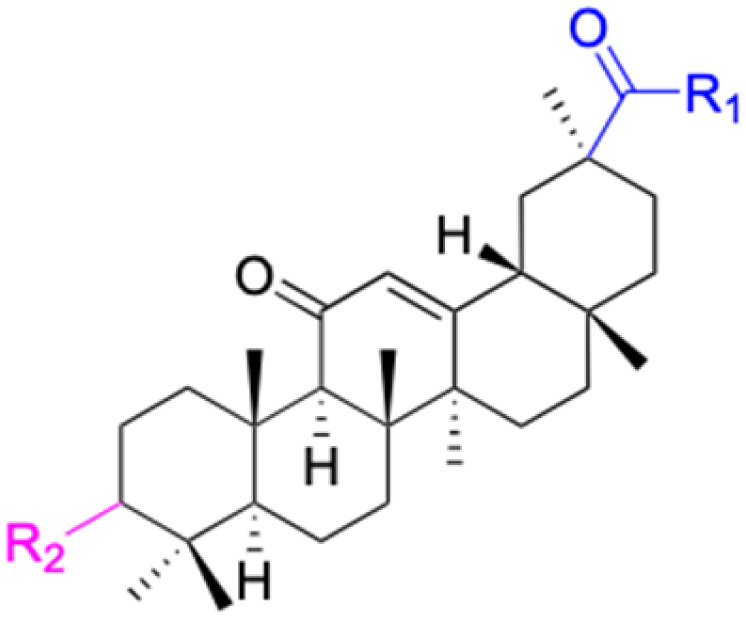	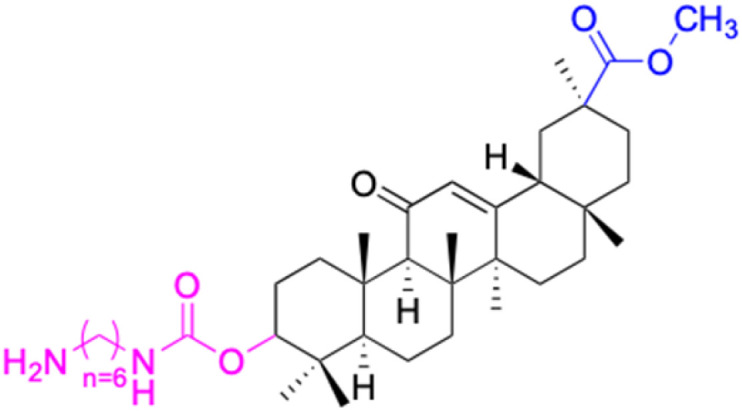		
Effects or mechanisms	44:	45:		
	R_1_ = O-i-Pr or OEt or OCH_3_ or OBn	518A2: IC_50_ = 1.0 μM, 8505C: IC_50_ = 1.6 μM, A253: IC_50_ = 1.1 μM		
	R_2_ = *O*-β-alanine or *O*-l-alanine or *O*-glycine	A2780: IC_50_ = 1.3 μM, A549: IC_50_ = 1.5 μM, DLD-1: IC_50_ = 0.91 μM		
	8505C: IC_50_ = 1.9–7.4 μM, A253: IC_50_ = 2.2–6.2 μM, A2780: IC_50_ = 1.3–5.9 μM	FADU: IC_50_ = 1.7 μM, HCT-116: IC_50_ = 1.1 μM, HCT-8: IC_50_ = 0.6 μM		
	A549: IC_50_ = 1.7–6.4 μM, DLD-1: IC_50_ = 2.5–8.5 μM, LIPO: IC_50_ = 2.3–7.5 μM	HT-29: IC_50_ = 0.5 μM, LIPO: IC_50_ = 1.5 μM, MCF-7: IC_50_ = 1.1 μM		
	Average:	SW1736: IC_50_ = 1.6 μM, SW480: IC_50_ = 2.2 μM		
	IC_50_ = 2.3–7.0 μM			
Reference	89	80		
Compounds	46–51	52–60		
Structure	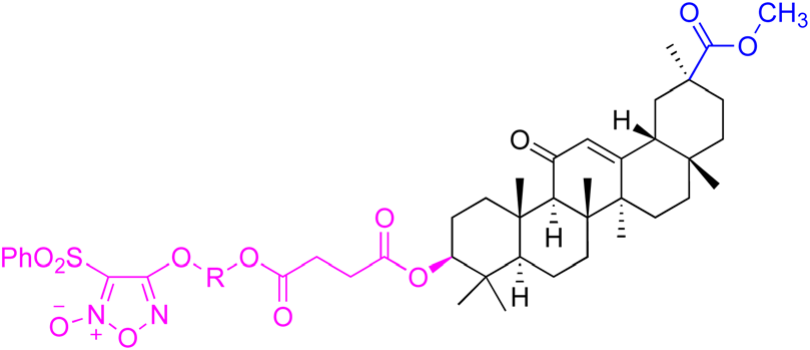	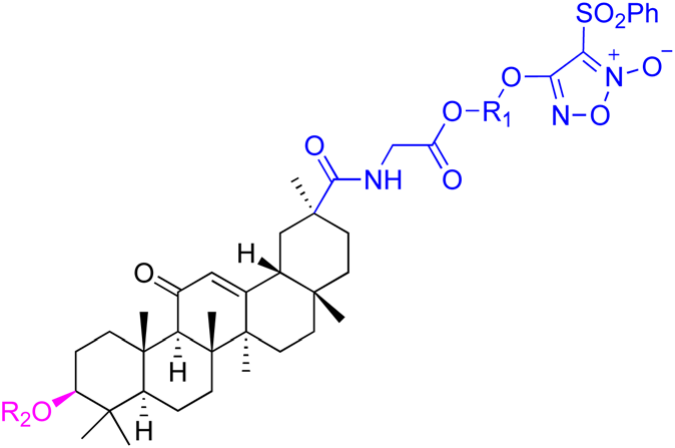		
Effects or mechanisms	46: R = (CH_2_)_2_O	52: R_1_ = (CH_2_)_2_, R_2_ = H		
	BEL7402: IC_50_ = 7.8 μM	HepG2: IC_50_ = 9.0 μM, BEL7402: IC_50_ = 1.3 μM		
	47: R = (CH_2_)_3_O	53: R_1_ = (CH_2_)_3_, R_2_ = H		
	BEL7402: IC_50_ = 9.2 μM	HepG2: IC_50_ = 3.7 μM, BEL7402: IC_50_ = 0.43 μM		
	48: R = (CH_2_)_2_CH(CH_3_)O	54: R_1_ = (CH_2_)_2_CH(CH_3_), R_2_ = H		
	BEL7402: IC_50_ = 6.0 μM	HepG2: IC_50_ = 3.0 μM, BEL7402: IC_50_ = 1.1 μM		
	49: R = (CH_2_)_4_O	55: R_1_ = (CH_2_)_4_, R_2_ = H		
	BEL7402: IC_50_ = 8.2 μM	HepG2: IC_50_ = 6.7 μM, BEL7402: IC_50_ = 0.25 μM		
	50: R = CH_2_CH=CHCH_2_O	56: R_1_ = (CH_2_)_2_O(CH_2_)_2_, R_2_ = H		
	HepG2: IC_50_ = 7.9 μM, BEL7402: IC_50_ = 7.3 μM	HepG2: IC_50_ = 5.1 μM, BEL7402: IC_50_ = 3.7 μM		
	51: R = CH_2_CH_2_NH	57: R_1_ = CH_2_CH=CHCH_2_, R_2_ = H		
	HepG2: IC_50_ = 2.9 μM, BEL7402: IC_50_ = 2.9 μM	HepG2: IC_50_ = 1.3 μM, BEL7402: IC_50_ = 0.32 μM		
		58: R_1_ = CH_2_CH=CHCH_2_, R_2_ = H		
		HepG2: IC_50_ = 3.3 μM, BEL7402: IC_50_ = 0.84 μM		
		59: R_1_ = (CH_2_)_4_, R_2_ = Ac		
		HepG2: IC_50_ = 8.3 μM, BEL7402: IC_50_ = 4.8 μM		
		60: R_1_ = (CH_2_)_2_O(CH_2_)_2_, R_2_ = H		
		HepG2: IC_50_ = 6.4 μM, BEL7402: IC_50_ = 9.4 μM		
Reference	64	64		
Compounds	61–62	63		
Structure	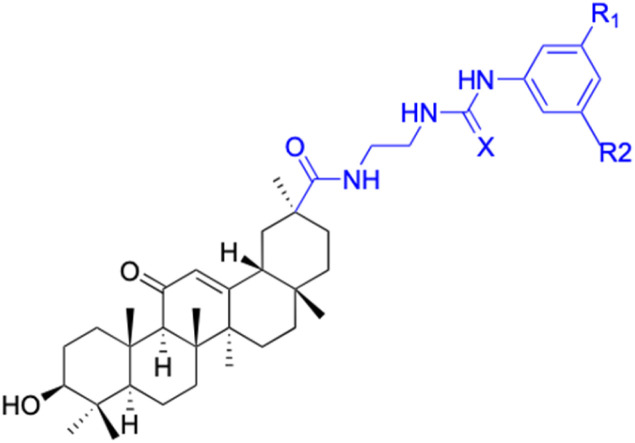	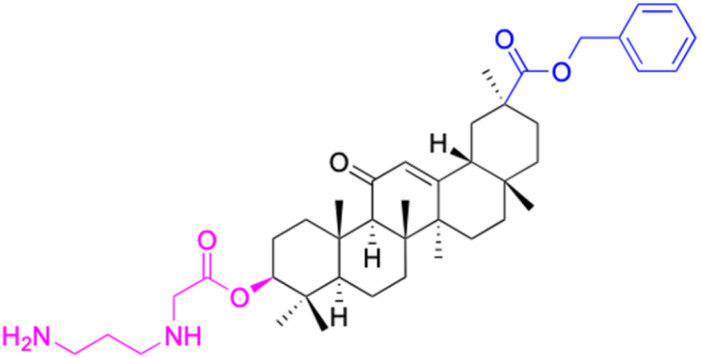		
Effects or mechanisms	61: R_1_ = R_2_ = CF_3_, X = O	63:		
	A549: IC_50_ = 7 μM, SKMEL: IC_50_ = 9 μM	518A2: IC_50_ = 5.1 μM, 8505C: IC_50_ = 2.0 μM, A253: IC_50_ = 1.9 μM		
	HS683: IC_50_ = 6 μM, U373: IC_50_ = 6 μM	A549: IC_50_ = 4.7 μM, DLD-1: IC_50_ = 4.9 μM, Lipo: IC_50_ = 2.9 μM		
	PC3: IC_50_ = 8 μM, MCF7: IC_50_ = 4 μM			
	816F10: IC_50_ = 4 μM			
	62: R_1_ = R_2_ = H, X = S			
	HS683: IC_50_ = 8 μM, PC3: IC_50_ = 9 μM			
Reference	74	90		
Compounds	64	65–66		
Structure	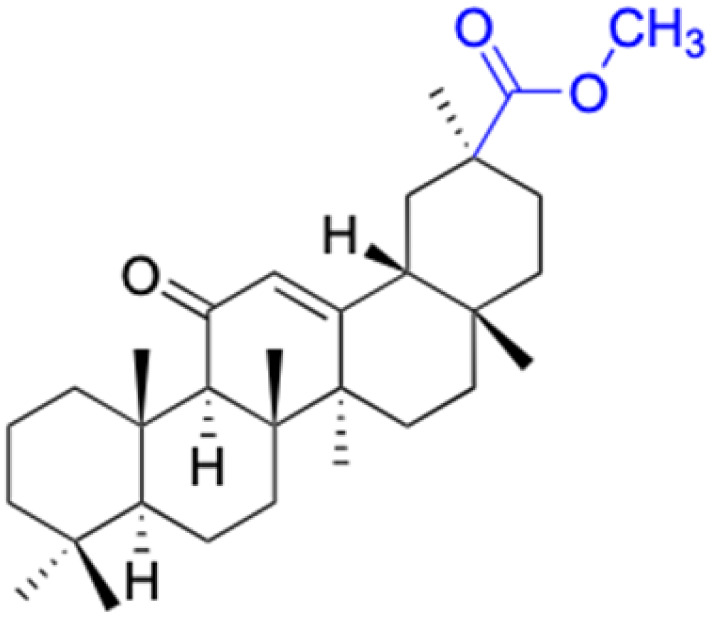	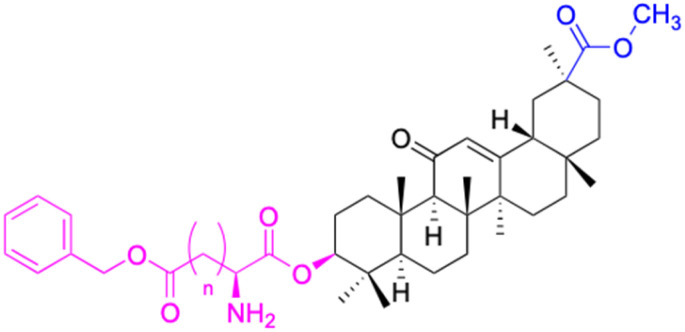		
Effects or mechanisms	64:	65: *n* = 1		
	518A2: IC_50_ = 23.69 μM, 8505C: IC_50_ = 24.30 μM, A2780: IC_50_ = 10.39 μM	A253: IC_50_ = 7.9 μM, A2780: IC_50_ = 8.8 μM, MCF-7: IC_50_ = 7.3 μM		
	LIPO: IC_50_ = 25.52 μM, SW1736: IC_50_ = 16.98 μM	66: *n* = 2		
		518A2: IC_50_ = 1.7 μM, 8505C: IC_50_ = 1.7 μM, A253: IC_50_ = 1.2 μM		
		A2780: IC_50_ = 1.6 μM, A549: IC_50_ = 1.7 μM, LIPO: IC_50_ = 1.7 μM		
		MCF-7: IC_50_ = 1.2 μM, SW1736: IC_50_ = 2.3 μM		
Reference	90	91		
Compounds	67–71	72		
Structure	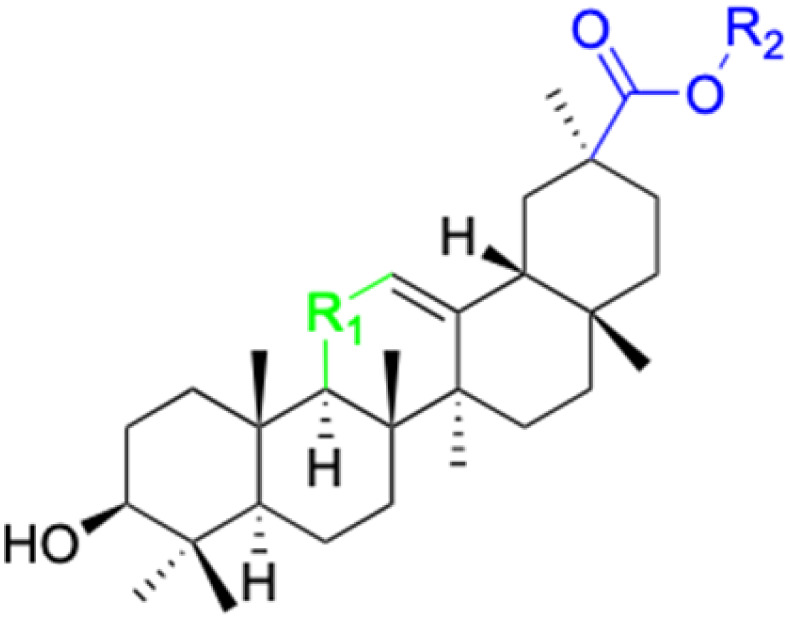	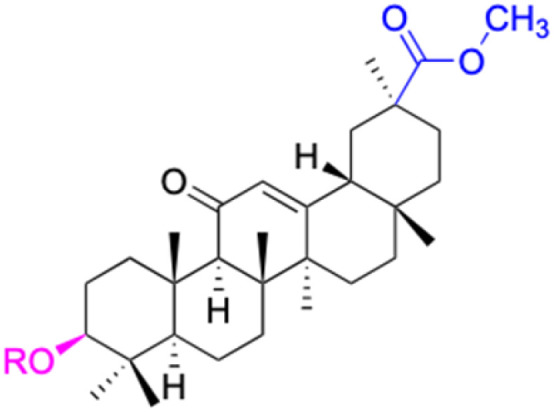		
Effects or mechanisms	67: R_1_ = CH_2_, R_2_ = H	72:		
	518A2: IC_50_ = 71.49 μM, 8505C: IC_50_ = 78.52 μM, A2780: IC_50_ = 62.78 μM	R = l-2,4-diaminobutanoyl or d-alanyl or sacrosyl or l-prolyl or l-phenylalanyl or l-methionyl or l-ornithyl or l-lysyl		
	A431: IC_50_ = 86.13 μM, A549: IC_50_ = 79.13 μM, DLD-1: IC_50_ = 90.50 μM	8505C: IC_50_ = 2.4–9.6 μM, A253: IC_50_ = 2.2–7.4 μM, A2780: IC_50_ = 1.5–5.5 μM		
	HCT-116: IC_50_ = 87.70 μM, HCT-8: IC_50_ = 88.76 μM, HT-29: IC_50_ = 90.30 μM	A549: IC_50_ = 2.1–9.9 μM, DLD-1: IC_50_ = 1.4–8.7 μM, LIPO: IC_50_ = 0.8–7.9 μM		
	LIPO: IC_50_ = 73.88 μM, MCF-7: IC_50_ = 90.19 μM, SW1736: IC_50_ = 72.47 μM	MCF-7: IC_50_ = 2.2–6.0 μM		
	NIH 3T3: IC_50_ = 68.70 μM			
	68: R_1_ = C=O, R_2_ = CH_3_			
	518A2: IC_50_ = 27.54 μM, 8505C: IC_50_ = 26.07 μM, A2780: IC_50_ = 25.54 μM			
	A431: IC_50_ = 25.28 μM, A549: IC_50_ = 23.50 μM, DLD-1: IC_50_ = 26.12 μM			
	HCT-116: IC_50_ = 22.10 μM, HCT-8: IC_50_ = 24.36 μM, HT-29: IC_50_ = 27.54 μM			
	LIPO: IC_50_ = 20.47 μM, MCF-7: IC_50_ = 22.14 μM, SW1736: IC_50_ = 34.87 μM			
	NIH 3T3: IC_50_ = 22.81 μM			
	69: R_1_ =CH_2_, R_2_ = CH_3_			
	518A2: IC_50_ = 34.54 μM, 8505C: IC_50_ = 33.88 μM, A2780: IC_50_ = 23.58 μM			
	A431: IC_50_ = 33.55 μM, A549: IC_50_ = 31.59 μM, DLD-1: IC_50_ = 31.73 μM			
	HCT-116: IC_50_ = 31.82 μM, HCT-8: IC_50_ = 31.34 μM, HT-29: IC_50_ = 23.89 μM			
	LIPO: IC_50_ = 34.81 μM, MCF-7: IC_50_ = 34.37 μM, SW1736: IC_50_ = 32.35 μM			
	NIH 3T3: IC_50_ = 42.22 μM			
	70: R_1_ = C=O, R_2_ = Et			
	518A2: IC_50_ = 25.23 μM, 8505C: IC_50_ = 24.58 μM, A2780: IC_50_ = 26.96 μM			
	A431: IC_50_ = 23.45 μM, A549: IC_50_ = 22.74 μM, DLD-1: IC_50_ = 28.14 μM			
	HCT-116: IC_50_ = 21.58 μM, HCT-8: IC_50_ = 43.42 μM, HT-29: IC_50_ = 22.14 μM			
	LIPO: IC_50_ = 27.66 μM, MCF-7: IC_50_ = 18.61 μM, SW1736: IC_50_ = 13.37 μM			
	NIH 3T3: IC_50_ = 23.66 μM			
	71: R_1_ = CH–OH, R_2_ = Et			
	518A2: IC_50_ = 51.52 μM, 8505C: IC_50_ = 52.80 μM, A2780: IC_50_ = 57.01 μM			
	A431: IC_50_ = 46.55 μM, A549: IC_50_ = 48.97 μM, DLD-1: IC_50_ = 52.80 μM			
	HCT-116: IC_50_ = 47.78 μM, HCT-8: IC_50_ = 44.32 μM, HT-29: IC_50_ = 44.32 μM			
	LIPO: IC_50_ = 52.80 μM, MCF-7: IC_50_ = 48.97 μM, SW1736: IC_50_ = 45.48 μM			
	NIH 3T3: IC_50_ = 43.16 μM			
Reference	91	92		
Compounds	73	74		
Structure	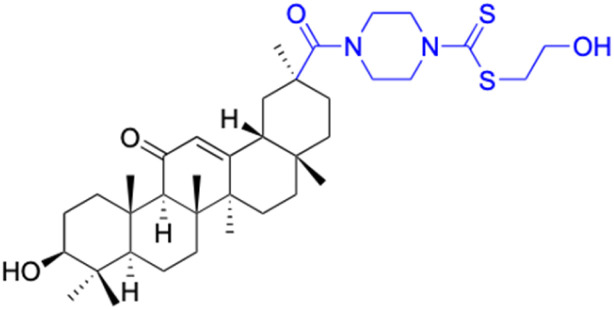	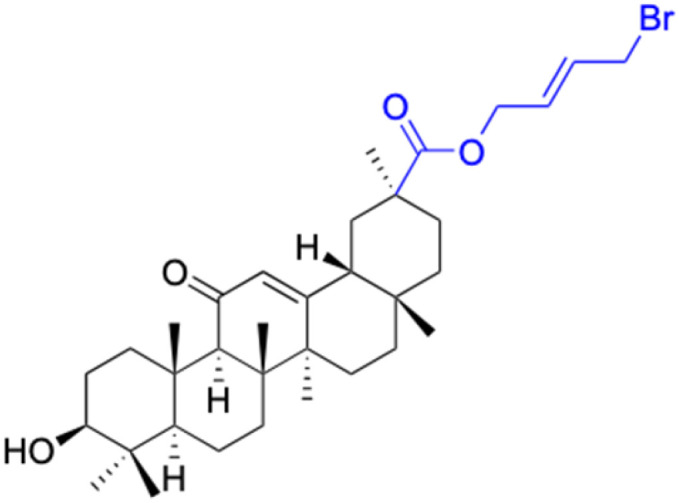		
Effects or mechanisms	73:	74:		
	SMMC-7721 (after 72 h): IC_50_ = 14.42 μg mL^−1^	8505C: IC_50_ = 8.8 μM, SW1736: IC_50_ = 1.8 μM		
Reference	73–76	93		
Compounds	75	76		
Structure	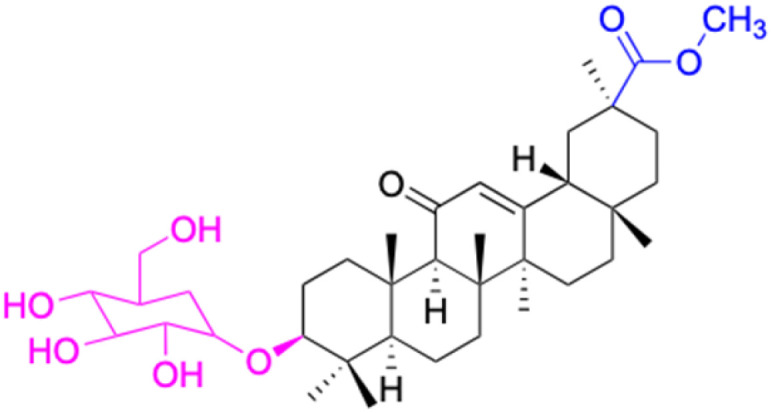	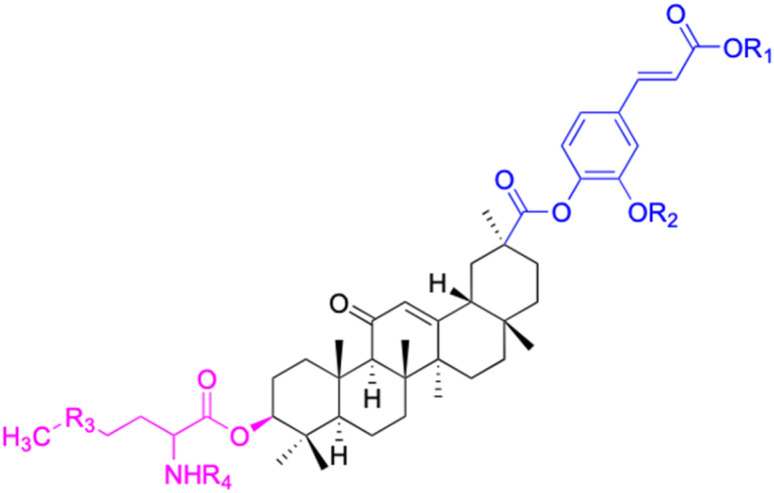		
Effects or mechanisms	75:	76:		
	MCF-7: IC_50_ = 1.8–8.6 μM	R_1_ = CH_3_ or Et, R_2_ = CH_3_ or H, R_3_ = S or Se, R_4_ = CO_2_*t*Bu or H		
	MDA-MB-231: IC_50_ = 1.3–6.4 μM	MCF-7: IC_50_ = 1.8–8.6 μM		
		MDA-MB-231: IC_50_ = 1.3–6.4 μM		
Reference	75	94		
Compounds	80–90			
Structure	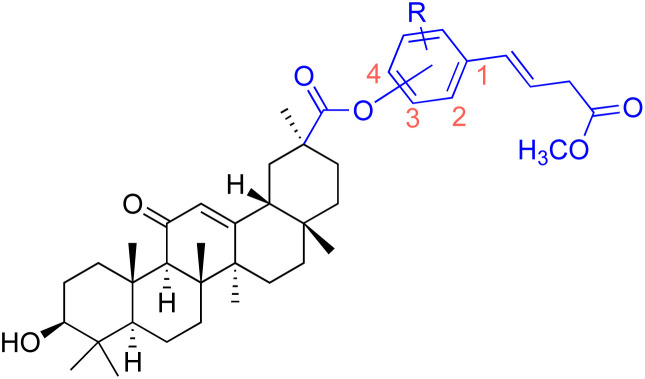			
Effects or mechanisms	80: 18β-GAO-4	87: 18β-GAO-3		
	R = H	R = 4-OCH_3_		
	A549: IC_50_ = 2.0 μM, SKMEL: IC_50_ = 3.0 μM, T98G: IC_50_ = 3.0 μM	KB: ED_50_ = 0.9 μM, KB-VIN: ED_50_ = 1.9 μM, A549: ED_50_ = 2.8 μM		
	HS683: IC_50_ = 3.0 μM, U373: IC_50_ = 2.0 μM, PC3: IC_50_ = 2.0 μM	1A9: ED_50_ = 1.6 μM, HCT-8: ED_50_ = 2.0 μM, ZR-751: ED_50_ = 1.9 μM		
	MCF7: IC_50_ = 3.0 μM, 816F10: IC_50_ = 3.0 μM	PC-3: ED_50_ = 2.8 μM, DU-145: ED_50_ = 9.9 μM, LN-Cap: ED_50_ = 6.5 μM		
	81: 18β-GAO-4	88: 18β-GAO-3		
	R = 3-OCH_3_	R = 3-OEt		
	MDA-MB-231: IC_50_ = 5.0 μM	KB: ED_50_ = 1.8 μM, KB-VIN: ED_50_ = 1.7 μM, A549: ED_50_ = 1.7 μM		
	82: 18β-GAO-4	1A9: ED_50_ = 1.1 μM, HCT-8: ED_50_ = 2.7 μM, ZR-751: ED_50_ = 5.2 μM		
	R = 3-OEt	PC-3: ED_50_ = 3.3 μM, DU-145: ED_50_ = 5.8 μM, LN-Cap: ED_50_ = 1.1 μM		
	MDA-MB-231: IC_50_ = 8.1 μM	89: 18β-GAO-2		
	83: 18β-GAO-3	R = 3-OCH_3_		
	R = 4-OCH_3_	KB: ED_50_ = 0.8 μM, KB-VIN: ED_50_ = 2.8 μM, A549: ED_50_ = 2.2 μM		
	MCF-7: IC_50_ = 8.5 μM, MDA-MB-231: IC_50_ = 7.3 μM	1A9: ED_50_ = 0.8 μM, HCT-8: ED_50_ = 1.9 μM, ZR-751: ED_50_ = 3.0 μM		
	84: 18β-GAO-3	PC-3: ED_50_ = 1.1 μM, DU-145: ED_50_ = 3.6 μM, LN-Cap: ED_50_ = 2.8 μM		
	R = 4-OEt	90: 18β-GAO-2		
	MDA-MB-231: IC_50_ = 9.4 μM	R = 3-F		
	85: 18β-GAO-4	KB: ED_50_ = 3.0 μM, KB-VIN: ED_50_ = 8.7 μM, A549: ED_50_ = 3.2 μM		
	R = 3-OCH_3_	1A9: ED_50_ = 1.3 μM, HCT-8: ED_50_ = 2.2 μM, ZR-751: ED_50_ = 2.7 μM		
	KB: ED_50_ = 1.6 μM, KB-VIN: ED_50_ = 2.5 μM, A549: ED_50_ = 2.0 μM	PC-3: ED_50_ = 1.6 μM, DU-145: ED_50_ = 2.7 μM, LN-Cap: ED_50_ = 4.4 μM		
	1A9: ED_50_ = 0.9 μM, HCT-8: ED_50_ = 1.7 μM, ZR-751: ED_50_ = 2.8 μM			
	PC-3: ED_50_ = 1.4 μM, DU-145: ED_50_ = 3.1 μM, LN-Cap: ED_50_ = 0.6 μM			
	86: 18β-GAO-2			
	R = 3-OEt			
	KB: ED_50_ = 2.9 μM, A549: ED_50_ = 3.0 μM, 1A9: ED_50_ = 1.8 μM			
	HCT-8: ED_50_ = 4.9 μM, ZR-751: ED_50_ = 8.8 μM, PC-3: ED_50_ = 3.5 μM			
	LN-Cap: ED_50_ = 6.8 μM			
Reference	75 and 76			
Compounds	91–93	94–99		
Structure	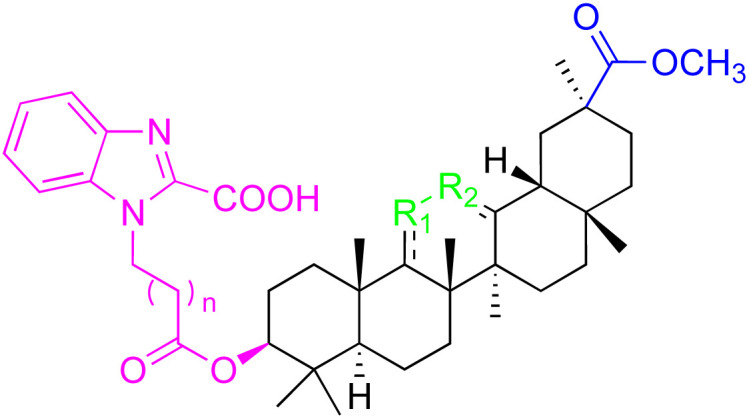	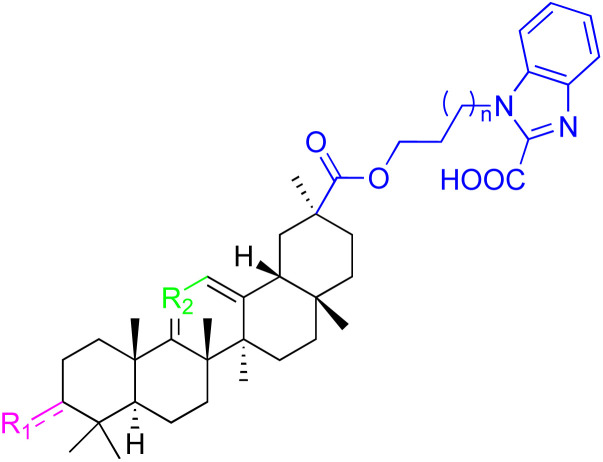		
Effects or mechanisms	91: R_1_ = C=O, R_2_ = CH, *n* = 0	94: R_1_ = B-OAc, R_2_ = C=O, *n* = 1		
	Pin1 inhibition: IC_50_ = 1.0 μM	Pin1 inhibition: IC_50_ = 1.3 μM		
	PC-3: IC_50_ = 7.80 μM	95: R_1_ = B-OAc, R_2_ = C=O, *n* = 0		
	92: R_1_ = CH, R_2_ = CH, *n* = 0	Pin1 inhibition: IC_50_ = 1.0 μM		
	Pin1 inhibition: IC_50_ = 2.3 μM	96: R_1_ = B–OH, R_2_ = CH_2_, *n* = 1		
	93: R_1_ = CH_2_, R_2_ = C=O, n= 1	Pin1 inhibition: IC_50_ = 2.8 μM		
	Pin1 inhibition: IC_50_ = 2.3 μM	97: R_1_ = B-OAc, R_2_ = CH_2_, *n* = 0		
		Pin1 inhibition: IC_50_ = 2.1 μM		
		PC-3: IC_50_ = 3.52 μM, LNCaP: IC_50_ = 7.92 μM		
		98: R_1_ = B-OAc, R_2_ = CH_2_, *n* = 0		
		Pin1 inhibition: IC_50_ = 4.7 μM		
		99: R_1_ = O, R_2_ = CH_2_, *n* = 1		
		Pin1 inhibition: IC_50_ = 3.8 μM		
Reference	95	95		
Compounds	100	101		
Structure	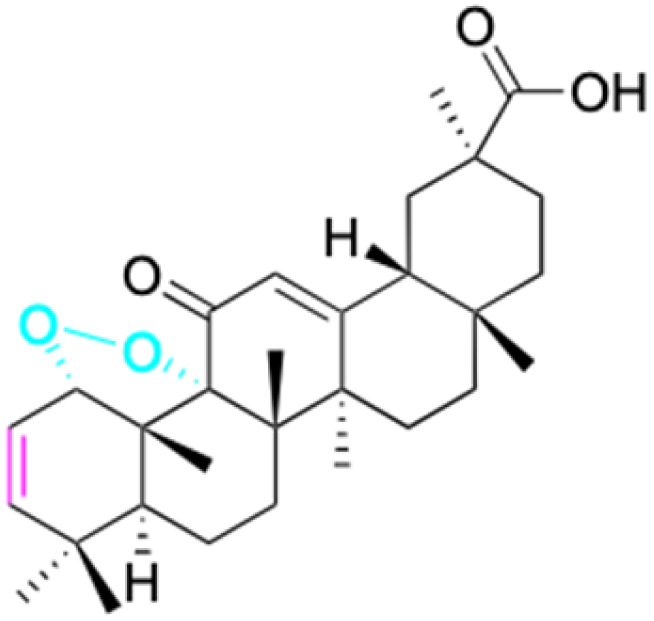	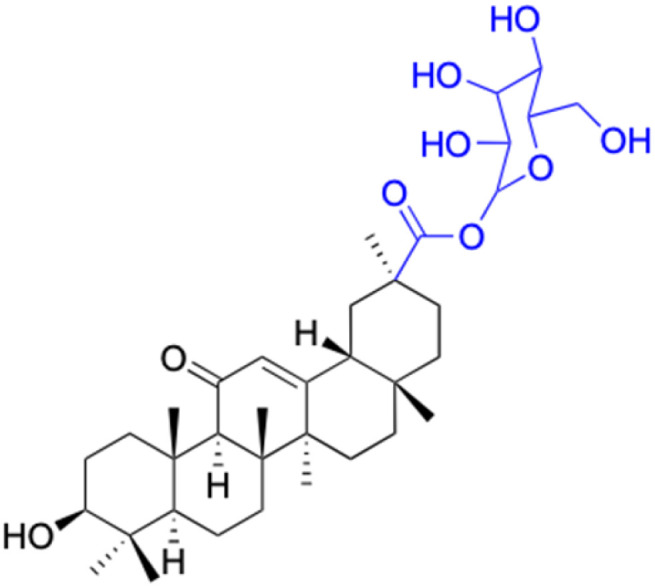		
Effects or mechanisms	100:	101:		
	A375: EC_50_ = 1.5 μM, A2780: EC_50_ = 1.0 μM, HT29: EC_50_ = 1.7 μM	HepG2: IC_50_ = 7.2 μM, MCF-7: IC_50_ = 7.7 μM		
	MCF7: EC_50_ = 2.9 μM, 518A2: EC_50_ = 1.2 μM			
Reference	81	69		
Compounds	102	103–108		
Structure	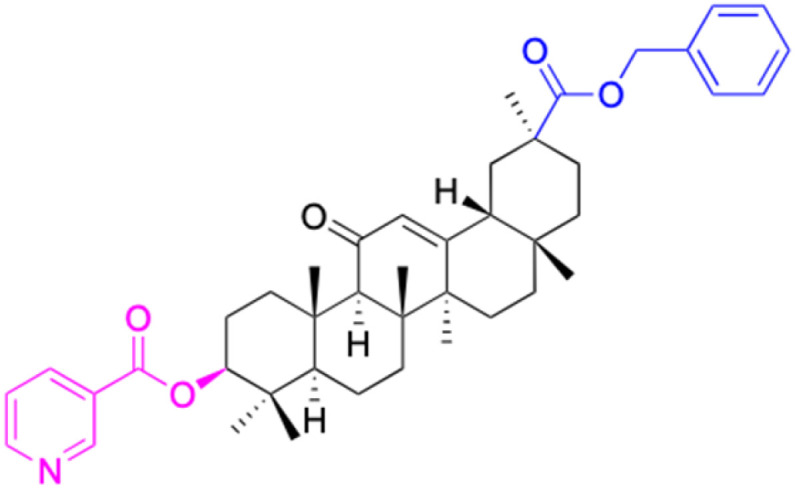	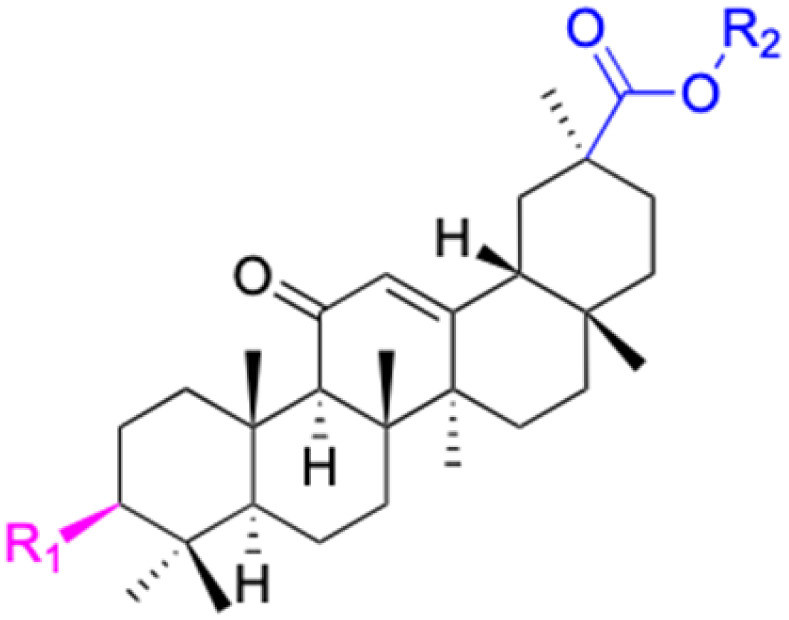		
Effects or mechanisms	102:	103: R_1_ = (*E*)-3-(4-acetoxyphenyl)acryl, R_2_ = Bn		
	SGC-7901: IC_50_ = 7.57 μM, MCF-7: IC_50_ = 5.51 μM, Eca-109: IC_50_ = 5.03 μM	HeLa: IC_50_ = 4.3 μM		
	HeLa: IC_50_ = 20.21 μM, Hep-G2: IC_50_ = 4.11 μM, HSF: IC_50_ = 23.18 μM	104: R_1_ = nicotinyl, R_2_ = Bn		
		SGC-7901: IC_50_ = 7.5 μM, MCF-7: IC_50_ = 5.5 μM		
		Eca-109: IC_50_ = 5.0 μM, Hep-G2: IC_50_ = 4.1 μM		
		105: R_1_ = isonicotinyl, R_2_ = Bn		
		MCF-7: IC_50_ = 8.6 μM, Hep-G2: IC_50_ = 8.7 μM		
		106: R_1_ = 3-acetoxybenzyl, R_2_ = Bn		
		HeLa: IC_50_ = 7.8 μM		
		107: R_1_ = 2-ethoxy-2-oxoacetyl, R_2_ = H		
		A-549: IC_50_ = 1.0 μM		
		108: R_1_ = dodecanyl, R_2_ = H		
		A-549: IC_50_ = 1.2 μM		
Reference	70 and 71	70 and 71		
Compounds	109–114	115		
Structure	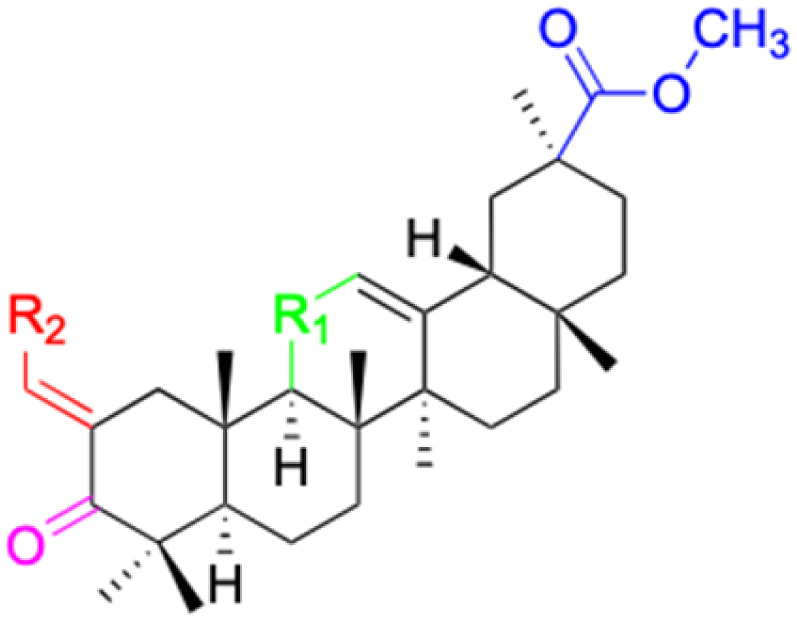	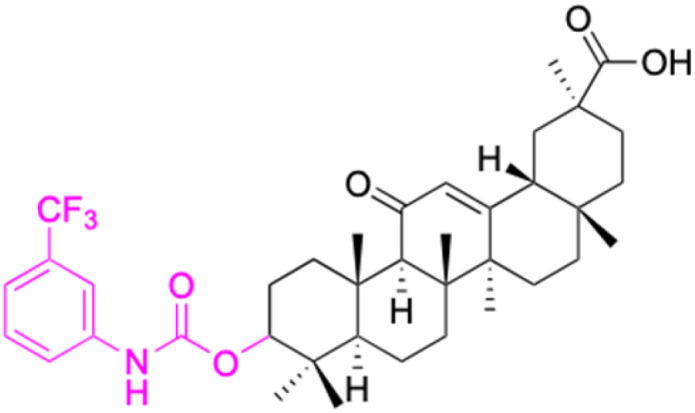		
Effects or mechanisms	109: R_1_ = C=O, R_2_ = 1-imidazolyl	115:		
	MCF7: IC_50_ = 6.4 μM, SH-SY5Y: IC_50_ = 6.0 μM, Jurkat: IC_50_ = 3.2 μM	A549: IC_50_ = 2.81 μM, HT29: IC_50_ = 3.19 μM, HepG2: IC_50_ = 5.55 μM		
	110: R_1_ = CH_2_, R_2_ = 1-imidazolyl	MCF-7: IC_50_ = 5.26 μM, PC-3: IC_50_ = 5.96 μM, Karpas299: IC_50_ = 5.59 μM		
	HT-29: IC_50_ = 3.3 μM, A549: IC_50_ = 2.8 μM, MIAPaca2: IC_50_ = 3.3 μM			
	HeLa: IC_50_ = 2.2 μM, A375: IC_50_ = 2.0 μM, MCF7: IC_50_ = 3.0 μM			
	HepG2: IC_50_ = 3.1 μM, SH-SY5Y: IC_50_ = 1.7 μM, Jurkat: IC_50_ = 1.1 μM			
	BJ: IC_50_ = 6.9 μM			
	111: R_1_ = C=O, R_2_ = 2-methyl-1-imidazolyl			
	HT-29: IC_50_ = 9.4 μM, A375: IC_50_ = 7.1 μM, MCF7: IC_50_ = 5.6 μM			
	SH-SY5Y: IC_50_ = 5.6 μM, Jurkat: IC_50_ = 2.4 μM			
	112: R_1_ = CH_2_, R_2_ = 2-methyl-1-imidazolyl			
	HT-29: IC_50_ = 3.6 μM, A549: IC_50_ = 3.1 μM, MIAPaca2: IC_50_ = 3.3 μM			
	HeLa: IC_50_ = 2.6 μM, A375: IC_50_ = 2.3 μM, MCF7: IC_50_ = 3.2 μM			
	HepG2: IC_50_ = 3.5 μM, SH-SY5Y: IC_50_ = 2.2 μM, Jurkat: IC_50_ = 1.3 μM			
	113: R_1_ = C=O, R_2_ = 1,2,3-triazolyl-4-methyl carboxylate			
	A375: IC_50_ = 7.2 μM, MCF7: IC_50_ = 6.0 μM, SH-SY5Y: IC_50_ = 3.7 μM			
	Jurkat: IC_50_ = 1.7 μM			
	114: R_1_ = CH_2_, R_2_ = 1,2,3-triazolyl-4-methyl carboxylate			
	HT-29: IC_50_ = 8.9 μM, A549: IC_50_ = 7.9 μM, MIAPaca2: IC_50_ = 6.9 μM			
	HeLa: IC_50_ = 5.4 μM, A375: IC_50_ = 4.9 μM, MCF7: IC_50_ = 5.2 μM			
	HepG2: IC_50_ = 9.0 μM, SH-SY5Y: IC_50_ = 3.2 μM, Jurkat: IC_50_ = 1.5 μM			
Reference	66	67		
Compounds	116–122	123–127	128–143	
Structure	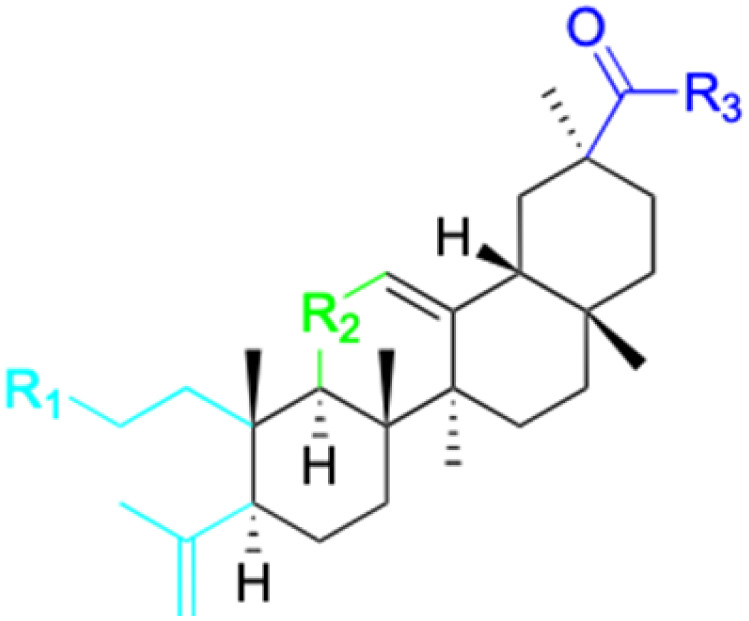	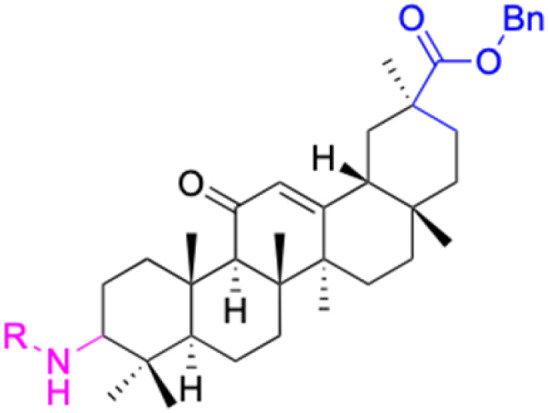	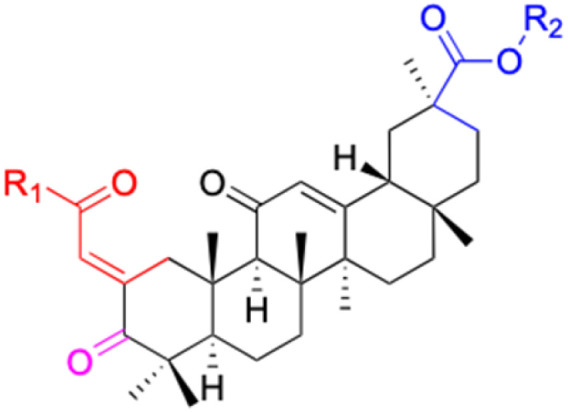	
Effects or mechanisms	116: R_1_ = CO_2_H, R_2_ = C=O, R_3_ = OBn	123: R = l-ala	128: R_1_ = OH, R_2_ = Bn	
	NTUB1: IC_50_ = 2.3 μM	A549: IC_50_ = 2.109 μM	MCF-7: IC_50_ = 3.8 μM, PC-3: IC_50_ = 1.6 μM	
	117: R_1_ = CO_2_ CH_3_, R_2_ = C=O, R_3_ = OBn	MCF-7: IC_50_ = 2.135 μM	129: R_1_ = OCH_3_, R_2_ = Bn	
	NTUB1: IC_50_ = 9.4 μM	HepG2: IC_50_ = 2.439 μM	MCF-7: IC_50_ = 1.1 μM, PC-3: IC_50_ = 1.2 μM	
	118: R_1_ = CO_2_H, R_2_ = C=O, R_3_ = NHC_6_H_5_	HeLa: IC_50_ = 2.39 μM	130: R_1_ = NHCH_3_, R_2_ = Bn	
	NTUB1: IC_50_ = 3.3 μM	MDCK: IC_50_ = 4.645 μM	MCF-7: IC_50_ = 1.1 μM, PC-3: IC_50_ = 0.40 μM	
	119: R_1_ = CO_2_H, R_2_ = C=O, R_3_ = NHCH(CH_3_)_2_	124: R = l-gly	131: R_1_ = NHEt, R_2_ = Bn	
	NTUB1: IC_50_ = 4.7 μM	A549: IC_50_ = 2.442 μM	MCF-7: IC_50_ = 0.59 μM, PC-3: IC_50_ = 0.27 μM	
	120: R_1_ = CO_2_CH_3_, R_2_ = H_2_, R_3_ = NHCH(CH_3_)CO_2_Me	MCF-7: IC_50_ = 2.853 μM	132: R_1_ = NH-nPr, R_2_ = Bn	
	Jurkat: IC_50_ = 9.6 μM	HepG2: IC_50_ = 3.472 μM	MCF-7: IC_50_ = 1.4 μM, PC-3: IC_50_ = 0.46 μM	
	121: R_1_ = CO_2_ CH_3_, R_2_ = CH_2,_ R_3_ = NHCH(CH_3_)CO_2_CH_3_	HeLa: IC_50_ = 3.01 μM	133; R_1_ = pyrrolidinyl, R_2_ = Bn	
	Jurkat: IC_50_ = 6.1 μM	MDCK: IC_50_ = 3.749 μM	MCF-7: IC_50_ = 3.0 μM, PC-3: IC_50_ = 3.4 μM	
	122: R_1_ = CO_2_Et, R_2_ = C=O, R_3_ = OEt	125: R = l-Boc-gly	134: R_1_ = morpholinyl, R_2_ = Bn	
	518A2: IC_50_ = 9.2 μM, A2780: IC_50_ = 5.8 μM	A549: IC_50_ = 2.751 μM	MCF-7: IC_50_ = 4.9 μM, PC-3: IC_50_ = 5.2 μM	
		MCF-7: IC_50_ = 3.811 μM	135: R_1_ = 1,4-bipiperidinyl, R_2_ = Bn	
		HepG2: IC_50_ = 3.306 μM	MCF-7: IC_50_ = 2.1 μM, PC-3: IC_50_ = 3.0 μM	
		HeLa: IC_50_ = 3.296 μM	136: R_1_ = piperazinyl, R_2_ = Bn	
		MDCK: IC_50_ = 4.431 μM	MCF-7: IC_50_ = 3.1 μM, PC-3: IC_50_ = 2.7 μM	
		126: R = l-phe	137: R_1_ = 1-methylpiperazinyl, R_2_ = Bn	
		A549: IC_50_ = 3.006 μM	MCF-7: IC_50_ = 3.3 μM, PC-3: IC_50_ = 3.1 μM	
		MCF-7: IC_50_ = 3.281 μM	138: R_1_ = 1-Boc-piperazinyl, R_2_ = Bn	
		HepG2: IC_50_ = 5.048 μM	MCF-7: IC_50_ = 0.44 μM, PC-3: IC_50_ = 0.23 μM	
		HeLa: IC_50_ = 3.296 μM	139: R_1_ = anilinyl, R_2_ = Bn	
		MDCK: IC_50_ = 5.024 μM	MCF-7: IC_50_ = 0.73 μM, PC-3: IC_50_ = 0.45 μM	
		127: R = l-pro	140: R_1_ = 4-nitroanilinyl, R_2_ = Bn	
		A549: IC_50_ = 3.261 μM	MCF-7: IC_50_ = 5.8 μM, PC-3: IC_50_ = 2.0 μM	
		MCF-7: IC_50_ = 7.623 μM	141: R_1_ = 4-chloroanilinyl, R_2_ = Bn	
		HepG2: IC_50_ = 2.143 μM	MCF-7: IC_50_ = 8.9 μM, PC-3: IC_50_ = 0.85 μM	
		HeLa: IC_50_ = 2.209 μM	142: R_1_ = 4-aminoperidinyl, R_2_ = Bn	
		MDCK: IC_50_ = 2.528 μM	MCF-7: IC_50_ = 0.98 μM, PC-3: IC_50_ = 0.69 μM	
			143: R_1_ = 1-Boc-piperazinyl, R_2_ = CH_3_	
			MCF-7: IC_50_ = 1.0 μM, PC-3: IC_50_ = 0.68 μM	
Reference	66 and 96	68	97	
Compounds	144	145–146		
Structure	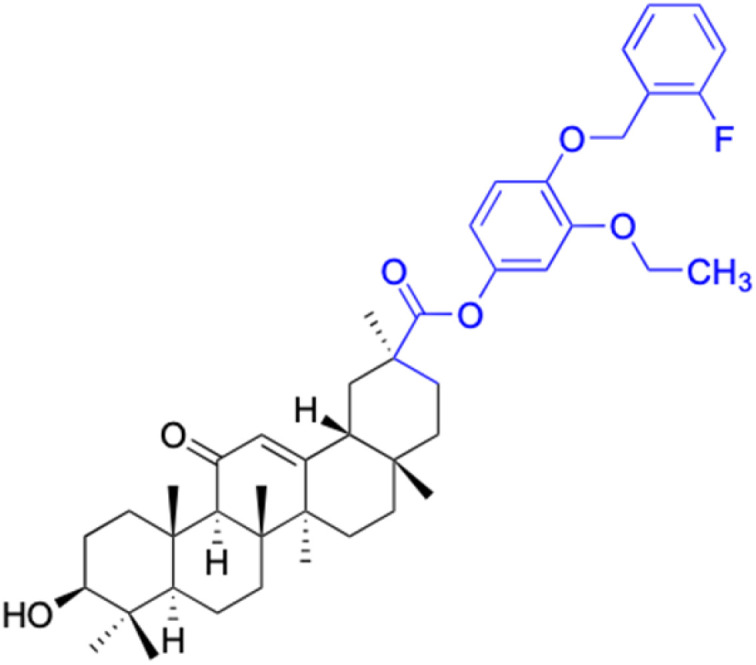	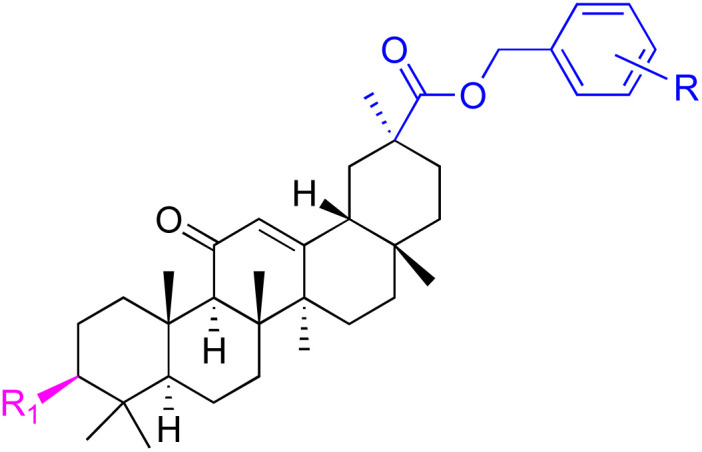		
Effects or mechanisms	144: HeLa: IC_50_ = 1.1 μM	145: R = 2,4-diCl, R_1_ = C=O		
		MDA-MB-231: IC_50_ = 9.6 μM		
		146: R= 3-OEt, 5-F, 4-(methoxymethyl)benzene, R_1_ = OH		
		HeLa: IC_50_ = 1.1 μM		
Reference	98	98		
Compounds	147	148	149	
Structure	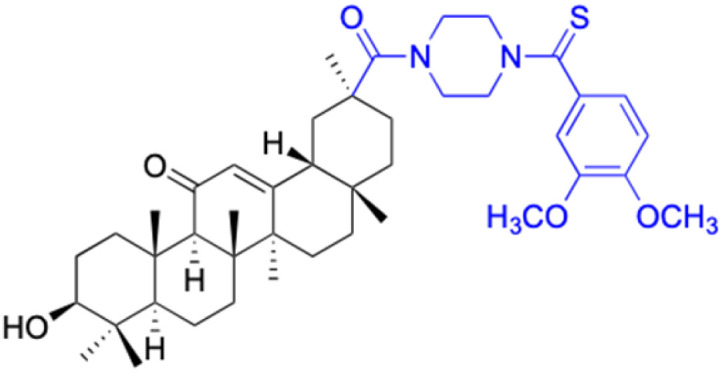	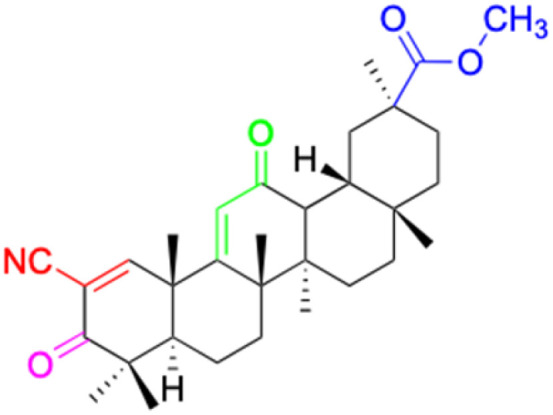	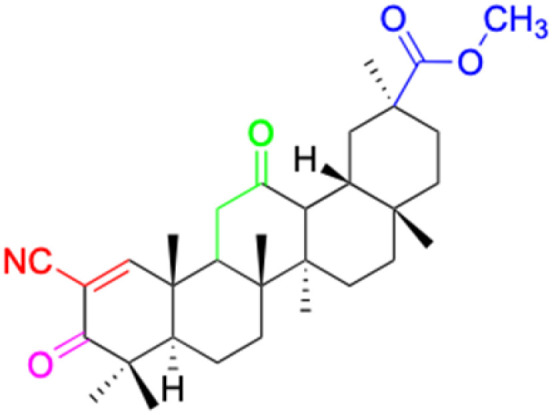	
Effects or mechanisms	147:	148:	149: KB-3-1: IC_50_ = 5.5 μM	
	Karpas299: IC_50_ = 6.51 μM, A549: IC_50_ > 40 μM	253JB-V: IC_50_ = 0.11 μM		
	HepG2: IC_50_ = 6.93 μM, MCF-7: IC_50_ = 18.85 μM	KU7: IC_50_ = 0.12 μM		
	PC-3: IC_50_ = 18.18 μM	Panc-1: IC_50_ = 0.07 μM		
		Panc-28: IC_50_ = 0.05 μM		
		KB-3-1: IC_50_ = 0.3 μM		
		KB-8-5: IC_50_ = 1.2 μM		
		HeLa: IC_50_ = 1.3 μM		
		MCF-7: IC_50_ = 5 μM		
		SK-N-MC: IC_50_ = 0.8 μM		
		MDA-MB-231: IC_50_ = 5.97 μM		
Reference	72	99	99	
Compounds	150	151		
Structure	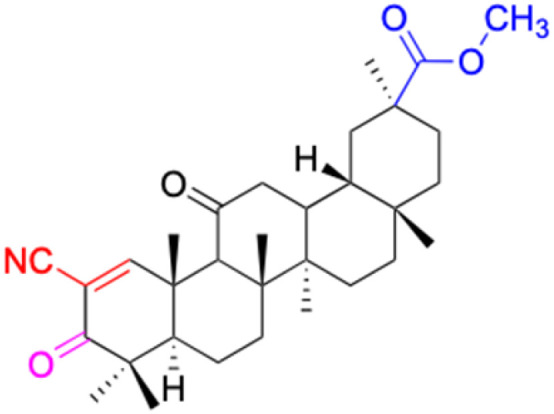	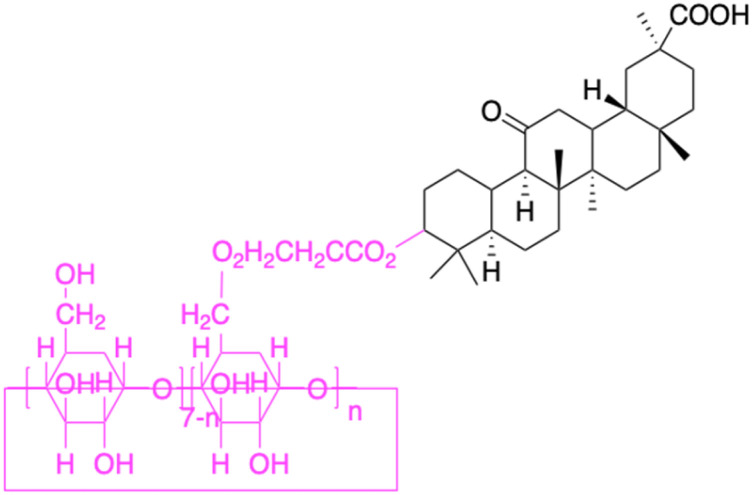		
Effects or mechanisms	150: KB-3-1: IC_50_ = 5.5 μM	151: Hep3B: cytotoxicity (28% cell viability)		
Reference	84–87	77		
Compounds	152	153		
Structure	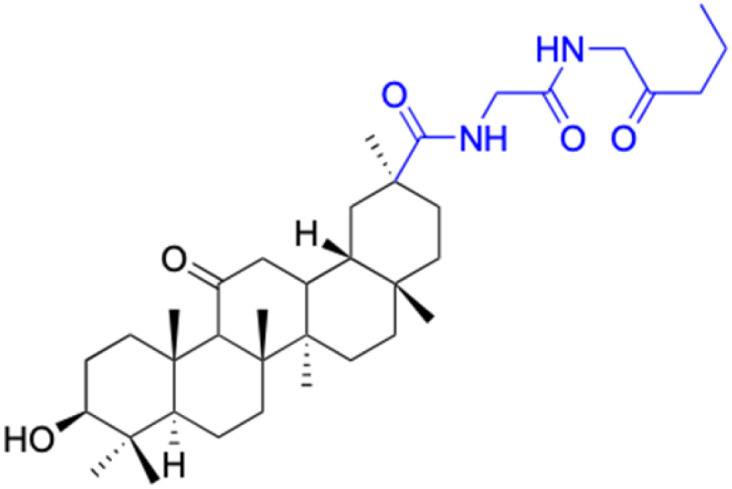	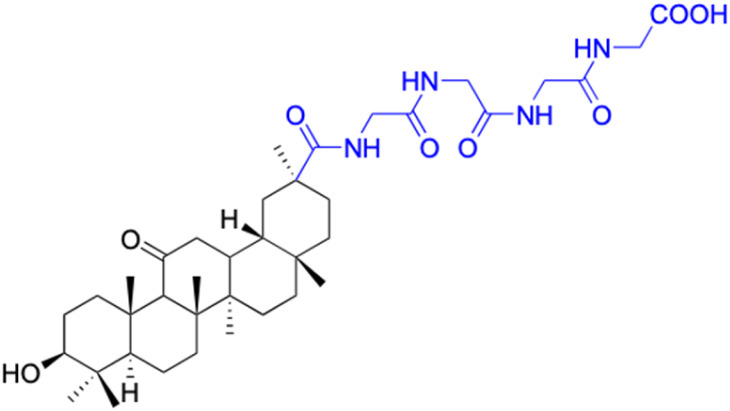		
Effects or mechanisms	152:	153:		
	MCF-7: IC_50/_ = 5.1 μM, HCT-116: IC_50_ = 7.40 μM	MCF-7: IC_50_ = 5.0 μM, HCT-116: IC_50_ = 5.2 μM		
Reference	79	100		
Compounds	154	155–156		
Structure	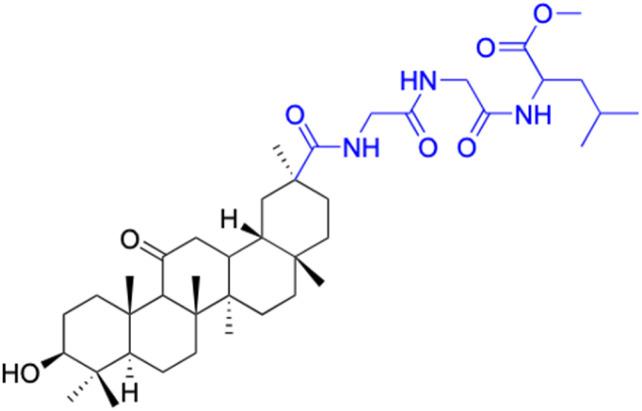	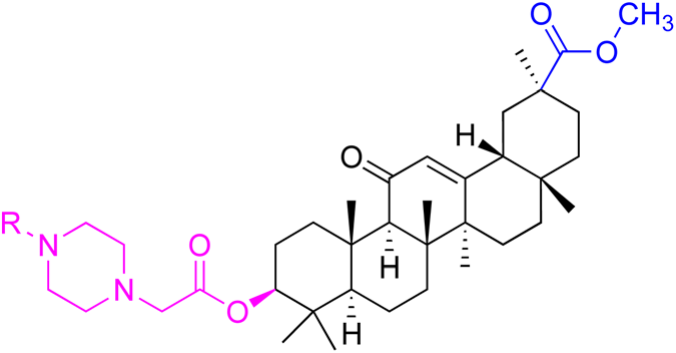		
Effects or mechanisms	154:	155: R = CH_3_		
	MCF-7: IC_50_ = 3.70 μM, HCT-116: IC_50_ = 3.0 μM, HepG-2: IC_50_ = 3.30 μM	MCF-7: IC_50_ = 6.9 μM, HepG2: IC_50_ = 9.9 μM		
		156: R = 4-(trifluoromethyl)benzene		
		MCF-7: IC_50_ = 9.5 μM, HepG2: IC_50_ = 25.6 μM		
Reference	79	101		
Abbreviations	HepG-2: hepatocellular carcinoma cell line. HCT-116: colorectal carcinoma cell line. MCF-7: breast adenocarcinoma cell line. MDCK: Madin–Darby canine kidney cell line. HeLa: cervical cancer cell line. A549: lung adenocarcinoma cell line. Hep3B: hepatocellular carcinoma cell line. SW1736: thyroid carcinoma cell line. LIPO: liposarcoma cell line. A2780: ovarian cancer cell line. 8505C: thyroid cancer cell line. 518A2: melanoma cell line. HSF: fibroblast cell line. Eca-109: esophageal carcinoma cell line. SGC-7901: gastric cancer cell line. average: unknown cell line. DU-145: prostate cancer cell line. Karpas299: lymphoma cell line. DLD-1: colorectal adenocarcinoma cell line. NIH 3T3: mouse embryonic fibroblast cell line. BEL7402: hepatocellular carcinoma cell line. SMMC-7721: hepatocellular carcinoma cell line. NTUB1: bladder cancer cell line. LN-Cap: prostate adenocarcinoma cell line. Jurkat: T-cell leukemia cell line. ZR-751: breast cancer cell line. KB: oral epidermoid carcinoma cell line. KB-VIN: multidrug-resistant oral epidermoid carcinoma cell line. A549: lung adenocarcinoma cell line. 1A9: human lymphoblastoid cell line. HCT-8: colorectal adenocarcinoma cell line. HT-29: colorectal adenocarcinoma cell line. CT-26: colorectal carcinoma cell line. PC-3: prostate cancer cell line. SKMEL: melanoma cell line. T98G: glioblastoma cell line. HS683: glioma cell line. U373: glioblastoma cell line. 816F10: melanoma cell line. Pin1: peptidyl–prolyl *cis*–*trans* isomerase NIMA-interacting 1. FADU: hypopharyngeal carcinoma cell line. Panc-28: pancreatic carcinoma-28 cell line. Panc-1: pancreatic carcinoma-1 cell line. 253JB-V: bladder carcinoma cell line. KU7: a cell line derived from human bladder cancer. SK-N-MC: human neuroblastoma cell line. KB-8-5: human epidermoid carcinoma cell line. KB-3-1: human epidermoid carcinoma cell line. SH-SY5Y: human neuroblastoma cell line. MIAPaca2: pancreatic carcinoma cell line. HL-60: human promyelocytic leukemia cell line			

In the realm of liver cancer treatment, researchers have discovered that 18β-GA holds significant potential due to its ability to exhibit toxicity against multiple liver cancer cell lines. A study conducted by Lai *et al.* found that 18β-GA derivatives 46–60 demonstrated selective cell toxicity against human hepatocellular carcinoma, hepG2 (hepatocellular carcinoma cell line) cells, and BEL-7402 (hepatocellular carcinoma cell line) cells.^64^ Similarly, derivatives 34, 101–102, 109–115, 123–127, and 147 displayed excellent cell toxicity against hepG2.^65–72^ Moreover, derivatives 73 and 74, which were modified at position C30, exhibited noteworthy cell toxicity against SMMC-7721 (hepatocellular carcinoma cell line).^73–76^ Researchers also discovered the complex of 18β-GA-conjugated-β-cyclodextrin and emodin's superior cell toxicity against hep3B (hepatocellular carcinoma cell line) cells when compared to emodin alone.^77^

In the domain of gastrointestinal cancers, encompassing those that affect the mouth, esophagus, colon, and stomach, the extraordinary cytotoxicity of 18β-GA and its derivatives has been strikingly demonstrated, particularly against colon cancer cell lines. The literature is replete with evidence of 18β-GA's potent effects on HCT-116 (colorectal carcinoma cell line), HCT-8 (colorectal adenocarcinoma cell line), DLD-1 (colorectal adenocarcinoma cell line), and HT-29 (colorectal adenocarcinoma cell line) cells. For instance, derivatives 152–154 and 45 exhibit toxicity towards HCT-116, with derivative 45 also affecting HCT-8 cells and DLD-1. Likewise, derivatives 109–125 display remarkable cytotoxicity towards HT-29 cells.^78–80^ Moreover, Seribian *et al.*'s study unveiled the high cytotoxicity of 18β-GA 1,9-peroxide on numerous human tumor cell lines, including HT-29 cells.^81^ Compounds 22–29 manifest substantial activity against Panc-1 (pancreatic carcinoma-1 cell line) and Panc-28 (pancreatic carcinoma-28 cell line) cells, and compounds 109–114 have been established as inhibitors of MIAPaca2 (pancreatic carcinoma cell line) cells.^66,67,82,83^ As for human oral epidermoid cancer cell lines, such as KB-3-1, KB-8-5, KB, and KB-VIN, compounds 85–90 and 148–150 have displayed their significant prowess.^74,76,84–87^

In the context of prostate cancer cell lines such as PC-3 (androgen-independent) and LN-Cap, compounds 61–62, 86–90, and 128–143 have demonstrated significant inhibitory effects.^75,76,97^ In ovarian cancer cell lines like A2780, compounds 64–71 exhibited inhibitory activity up to 1.5 μM.^90,91^ Notably, compounds 109–114, 103, 106,102, 144, and 146 displayed notable inhibitory activity against HeLa cells (cervical cancer cell line).^70,71,81,98^ Additionally, compounds 152–156 showed strong inhibitory activity against MCF-9 breast cancer cell line.^79,101^

Beyond these realms, GA and its derivatives have also exhibited their anticancer activity in other areas. Prior research has established that GA and its derivatives have the ability to inhibit Neurosystem-associated cancer cell lines, such as SH-SY5Y (human neuroblastoma cell line) and SK-N-MC (human neuroblastoma cell line).^66,84^ In the investigation conducted by Csuk *et al.* conducted an investigation, which found that GA and its derivatives displayed robust activity against thyroid cancer.^91^ Li *et al.* found that 18β-GA exert anticancer effects as pin1 inhibitors.^95^ Furthermore, GA and its derivatives have demonstrated significant inhibitory activity against various types of cancer cells including those associated with lung cancer, lymphoma, melanoma, and breast cancer.^66–68,74–76,80,82,83,89,91–94,96^

In conclusion, 18β-GA and its derivatives have shown promising anti-tumor properties in various types of cancer, including colorectal, breast, lung, and liver. The cytotoxic effects of 18β-GA have been attributed to its ability to induce apoptosis, cell cycle arrest, inhibit migration, and downregulate various signaling pathways involved in cancer progression. In addition, 18β-GA has been shown to enhance the cytotoxicity of conventional chemotherapeutic agents, making it a potential adjuvant therapy for cancer treatment. Although 18β-GA and its derivatives have shown potential as anti-tumor agents, further studies are needed to fully understand their mechanisms of action and to optimize their pharmacological properties for clinical applications.

## Antibacterial activity

The emergence and spread of drug-resistant bacteria pose a significant threat to global health. Conventional antibiotics are often rendered ineffective against these resistant strains, leading to prolonged and complicated treatment regimens, as well as increased morbidity and mortality rates. Consequently, there is a critical need to identify novel antibiotics that can effectively target and eliminate these drug-resistant bacteria.^102^ Researchers have turned their attention to natural compounds as potential sources of new antibiotics. Natural compounds have long been recognized for their diverse chemical structures and biological activities. By studying and modifying these compounds, scientists hope to develop more potent and effective antibiotics. Among the natural compounds explored for their antibacterial properties, 18β-GA and related compounds have shown promise. These compounds have exhibited antibacterial effects against various bacterial strains, suggesting their potential as therapeutic agents. Further investigations are underway to elucidate the mechanisms of action and optimize the activity of these compounds.^103^

The antimicrobial properties of 18β-GA, a compound extracted from the licorice plant, have been extensively studied by various researchers. Kim *et al.* discovered that 18β-GA has the ability to disrupt bacterial cell membranes, leading to the eradication of these microorganisms. This finding has generated significant interest in the potential of 18β-GA as a novel antibacterial agent.^104^ Salari *et al.* further supported the antibacterial activity of 18β-GA against periodontopathogenic and capnophilic bacteria, while another investigation found that this natural compound can inhibit the growth of *Helicobacter pylori*.^105,106^ In a comprehensive study, Schrader *et al.* explored the antibacterial properties of various natural plant compounds, including 18β-GA and 18α-GA, and evaluated their efficacy against common pathogens found in pond-cultured channel catfish.^107^ It has been demonstrated that 18β-GA can effectively combat antibiotic-resistant bacterial strains, such as methicillin-resistant *Staphylococcus aureus* (MRSA), by inhibiting their survival and virulence gene expression.^108^ Furthermore, this compound has shown potential in preventing the growth and formation of supragingival plaque bacteria and treating *H. pylori* infections.^109,110^ In the fight against opportunistic nosocomial *P. aeruginosa*, 18β-GA has proven to be a valuable ally.^111^ Additionally, 18β-GA has been investigated for its ability to enhance the activity of tobramycin and polymyxin B against MRSA.^112^ In the quest to combat opportunistic nosocomial *P. aeruginosa*, 18β-GA has been found to be a valuable ally.^113^ Moreover, 18β-GA has been used in combination with nanoparticles and hydrogels to combat bacterial infections. Darvishi *et al.* developed and evaluated the antibacterial activity of 18β-GA-loaded PL18β-GA nanoparticles, which demonstrated significant antibacterial activity against both Gram-positive and Gram-negative bacteria.^114^ Similarly, Zhao *et al.* engineered an injectable moldable hydrogel assembled from natural glycyrrhizic acid, which exhibited remarkable antibacterial activity against both types of bacteria.^115^ Recently, the remarkable antibacterial capabilities of 18β-GA derivatives have come to light. These derivatives have shown promising inhibitory effects against various bacterial strains, making them potential candidates for combating bacterial infections.^116^ In this review, our objective is to classify and elucidate the antibacterial activities of different 18β-GA derivatives against specific bacterial species. 18β-GA and its derivatives, as shown in Table 3, have demonstrated significant potential in inhibiting pathogens.

**Table 3:** Chemical structure and antibacterial activity of glycyrrhetinic acid and its derivatives 155–223

Compounds	18β-GA	157–163	164–166
Structure	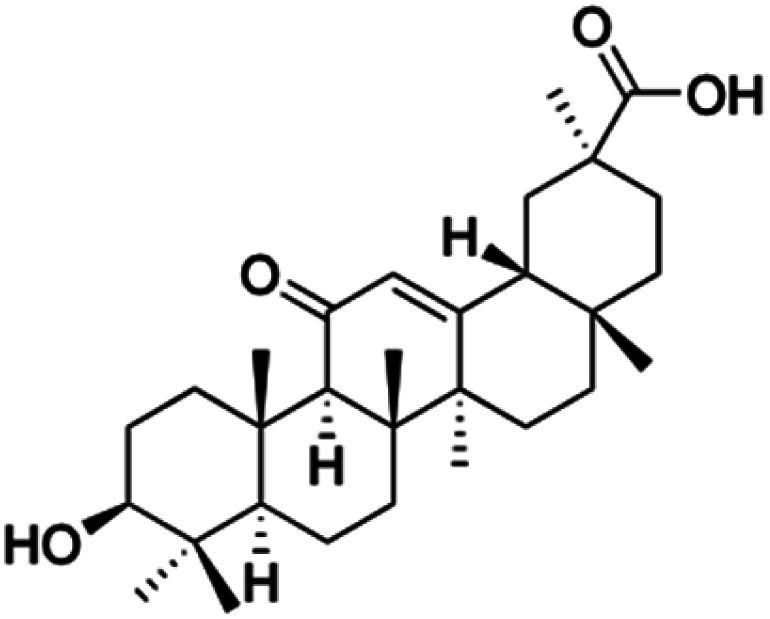	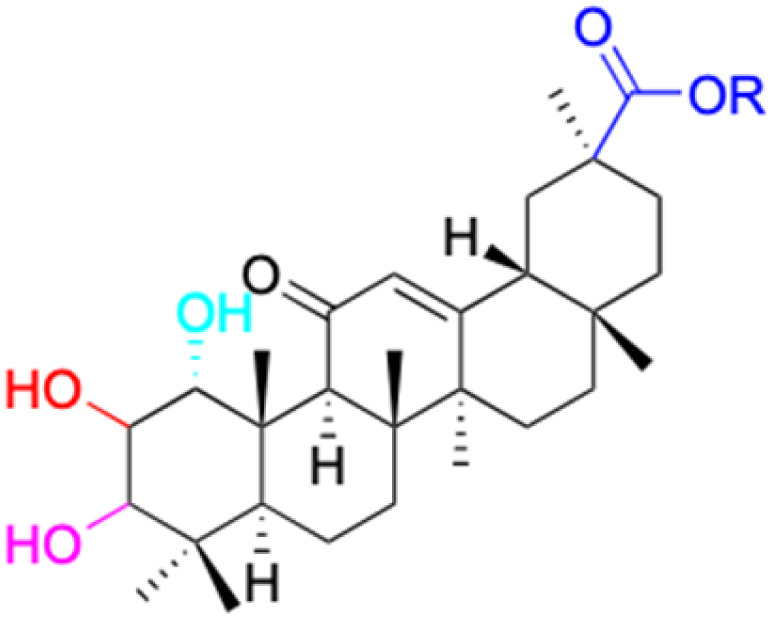	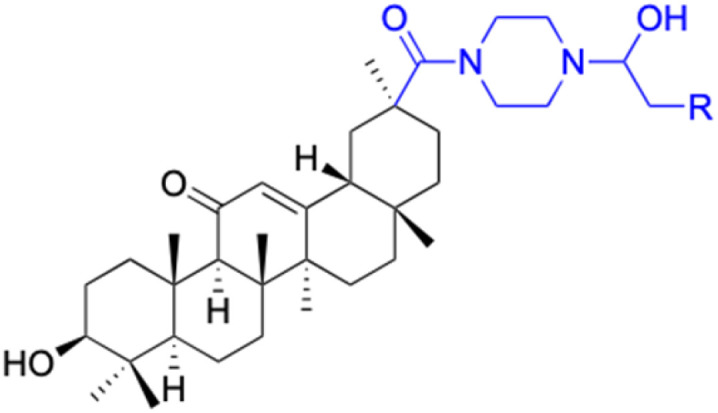
Effects or mechanisms	*Bacillus subtilis*: MIC = 7.6 μg mL^−1^	157: R = CH_2_CH_3_	164: R =
	*Staphylococcus*: MIC = 12.5 μg mL^−1^	*B. subtilis*: MIC = 16.9 μg mL^−1^	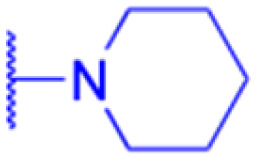
	*A. actinomycetemcomitans*:	*S. scabies*: MIC = 2.1 μg mL^−1^	Xoo: EC_50_ = 2.28 μg mL^−1^
	MIC = 8 μg mL^−1^	*S. aureus*: MIC = 4.2 μg mL^−1^	Xac: EC_50_ = 1.42 μg mL^−1^
	*E. corrodens*: MIC = 16 μg mL^−1^	MRSA: MIC = 4.0 μg mL^−1^	165: R =
	*C. sputigena*: MIC = 8 μg mL^−1^	158: R = (CH_2_)_2_CH_3_	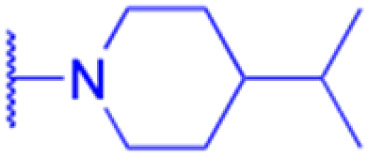
	*Edwardsiella ictaluri*:	*B. subtilis*: MIC = >34.8 μg mL^−1^	Xoo: EC_50_ = 3.57 μg mL^−1^
	MIC > 470.7 μg mL^−1^	*S. scabies*: MIC = 4.3 μg mL^−1^	Xac: EC_50_ = 0.93 μg mL^−1^
	*H. pylori*: MIC = 20.8 μg mL^−1^	*S. aureus*: MIC = 4.3 μg mL^−1^	166: R =
	*P. aeruginosa*: MIC = 160 μg mL^−1^	MRSA: MIC = 2.0 μg mL^−1^	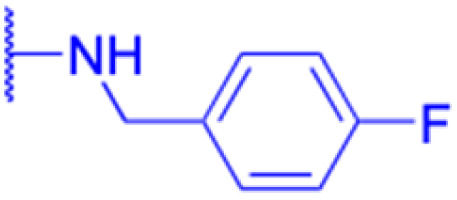
	*P. gingivalis* ATCC 33277:	159: R = (CH_2_)_3_CH_3_	Xoo: EC_50_ = 2.63 μg mL^−1^
	MIC = 64 μg mL^−1^	*B. subtilis*: MIC = >34.8 μg mL^−1^	Xac: EC_50_ = 2.31 μg mL^−1^
	*S. gordonii*: MIC = 64 μg mL^−1^	*S. scabies*: MIC = 4.3 μg mL^−1^	
	*N. gonorrhoeae*:	*S. aureus*: MIC = 4.3 μg mL^−1^ MRSA: MIC = 2.0 μg mL^−1^	
	MIC = 3.9–62.5 μg mL^−1^	160: R = CH_3_	
		*B. subtilis*: MIC = 4.0 μg mL^−1^	
		*S. scabies*: MIC = 1.0 μg mL^−1^	
		*S. aureus*: MIC = 2.0 μg mL^−1^	
		161: R = CH_2_CH_3_	
		*B. subtilis*: MIC = 2.0 μg mL^−1^	
		*S. scabies*: MIC = 4.1 μg mL^−1^	
		*S. aureus*: MIC = 1.0 μg mL^−1^	
		MRSA: MIC = 1.0 μg mL^−1^	
		162: R = CH(CH_3_)_2_	
		*B. subtilis*: MIC = >33.9 μg mL^−1^	
		*S. scabies*: MIC = 4.2 μg mL^−1^	
		*S. aureus*: MIC = 8.4 μg mL^−1^	
		MRSA: MIC = 2.0 μg mL^−1^	
		163: R = (CH_2_)_3_CH_3_	
		*B. subtilis*: MIC = >34.8 μg mL^−1^	
		*S. scabies*: MIC = 4.3 μg mL^−1^	
		*S. aureus*: MIC = >34.8 μg mL^−1^	
		MRSA: MIC = >32.0 μg mL^−1^	
Reference	104, 105, 107, 110, 111, 113 and 125	117	118
Compounds	167–171		172
Structure	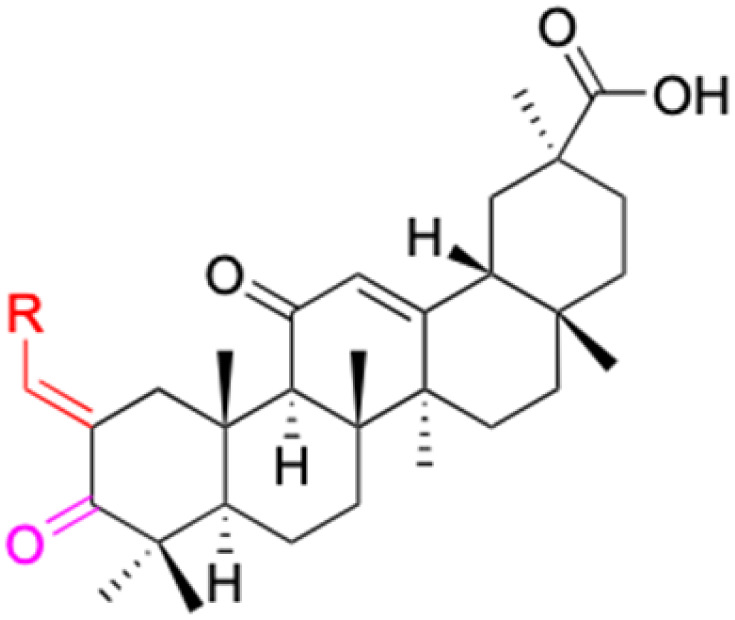		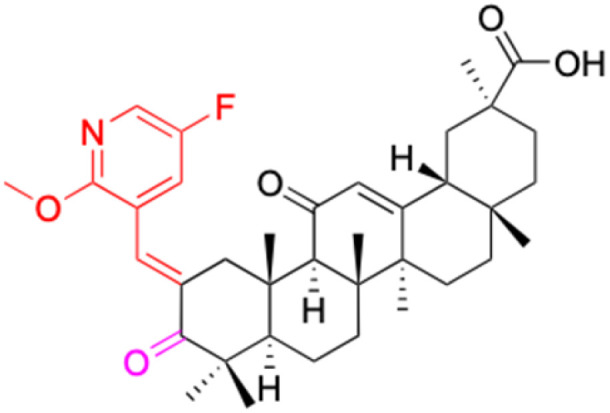
Effects or mechanisms	167: R =		172:
	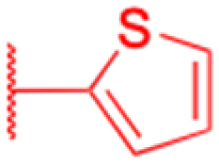		*Staphylococcus aureus* (ATCC 6538):
	*Staphylococcus aureus* (ATCC 6538): MIC = 54.88 μg mL^−1^		MIC = 6.25 μmol L^−1^
	*Staphylococcus aureus* (ATCC 29213): MIC = 6.86 μg mL^−1^		*Staphylococcus aureus* subsp. *aureus* (ATCC 29213):
	*Staphylococcus epidermidis* (ATCC 12228): MIC = 27.44 μg mL^−1^		MIC = 6.25 μmol L^−1^
	168: R =		*Staphylococcus epidermidis* (ATCC 12228):
	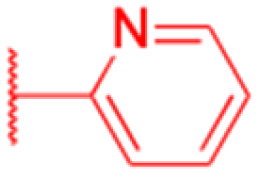		MIC = 6.25 μmol L^−1^
	*Staphylococcus aureus* (ATCC 6538): MIC = 3.39 μg mL^−1^		
	*Staphylococcus aureus* (ATCC 29213): MIC = 6.79 μg mL^−1^		
	*Staphylococcus epidermidis* (ATCC 12228): MIC = 3.39 μg mL^−1^		
	169: R =		
	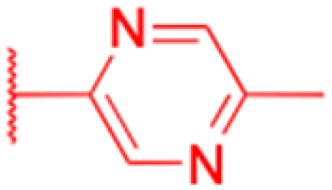		
	*Staphylococcus aureus* (ATCC 6538): MIC = 2.72 μg mL^−1^		
	*Staphylococcus aureus* (ATCC 29213): MIC = 2.72 μg mL^−1^		
	*Staphylococcus epidermidis* (ATCC 12228): MIC = 2.72 μg mL^−1^		
	170: R =		
	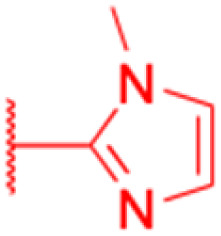		
	*Staphylococcus aureus* (ATCC 6538): MIC = 6.83 μg mL^−1^		
	*Staphylococcus aureus* (ATCC 29213): MIC = 13.67 μg mL^−1^		
	*Staphylococcus epidermidis* (ATCC 12228): MIC = 6.83 μg mL^−1^		
	171: R =		
	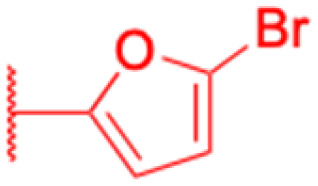		
	*Staphylococcus aureus* (ATCC 6538): MIC = 27.34 μg mL^−1^		
	*Staphylococcus aureus* (ATCC 29213): MIC = 54.68 μg mL^−1^		
	*Staphylococcus epidermidis* (ATCC 12228): MIC = 27.34 μg mL^−1^		
Reference	43		123
Compounds	173		174
Structure	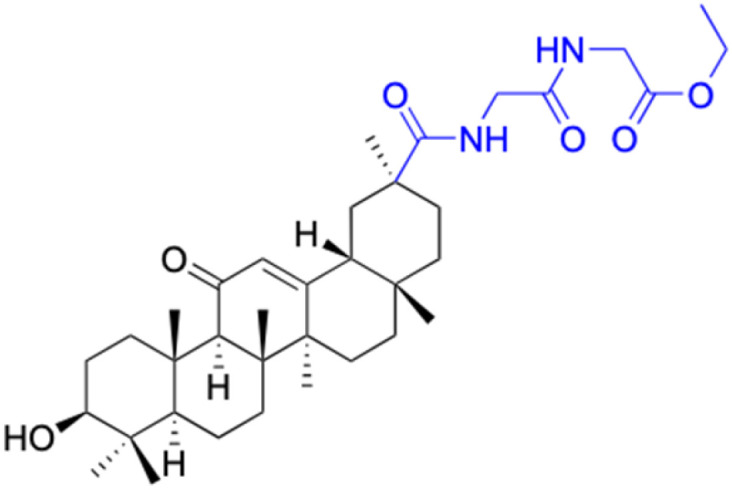		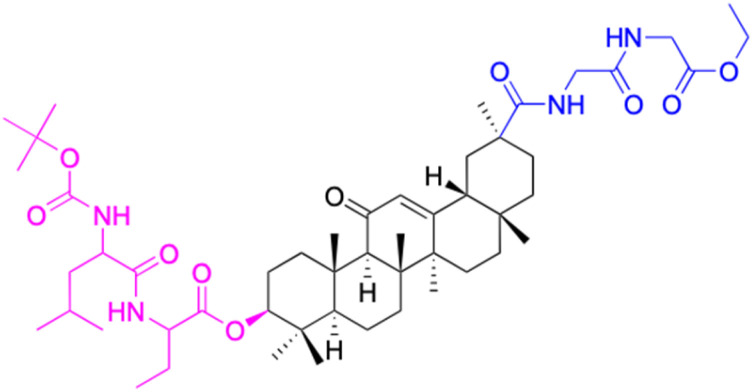
Effects or mechanisms	173:		174:
	*Streptococcus pneumonia* RCMB 010010:		*Streptococcus pneumonia* RCMB 010010:
	Diameter of inhibition zone = 15 mm		Diameter of inhibition zone = 12 mm
	*Staphylococcus aureus* ATCC25923:		*Staphylococcus aureus* ATCC25923:
	Diameter of inhibition zone = 15 mm		Diameter of inhibition zone = 17 mm
	*Micrococcus luteus*:		*Micrococcus luteus*:
	Diameter of inhibition zone = 30 mm		Diameter of inhibition zone = 30 mm
	*Escherichia coli* ATCC25922:		*Escherichia coli* ATCC25922:
	Diameter of inhibition zone = 20 mm		Diameter of inhibition zone = 18 mm
	*Pseudomonas aeruginosa* ATCC7853:		*Pseudomonas aeruginosa* ATCC7853:
	Diameter of inhibition zone = 18 mm		Diameter of inhibition zone = 15 mm
Reference	79		79
Compounds	175		176
Structure	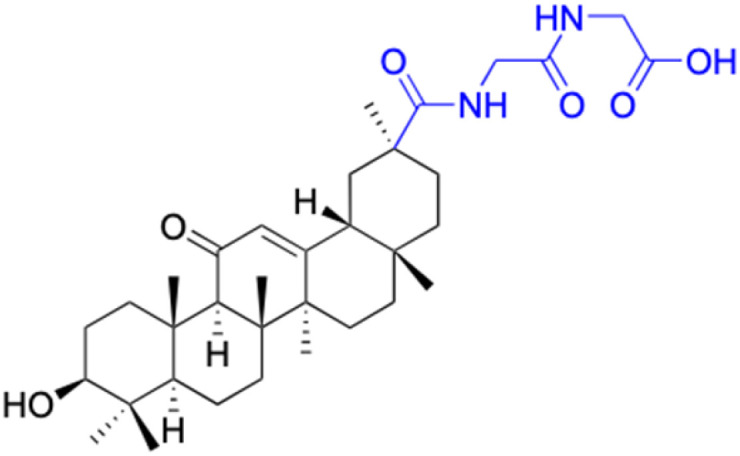		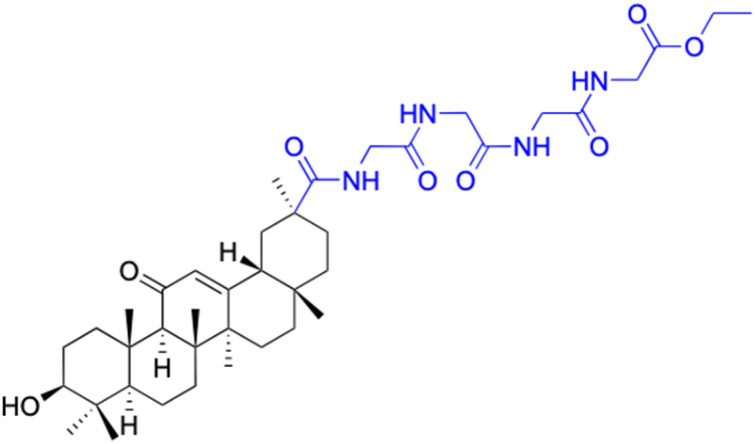
Effects or mechanisms	175:		176:
	*Streptococcus pneumonia* RCMB 010010:		*Streptococcus pneumonia* RCMB 010010:
	Diameter of inhibition zone = 17 mm		Diameter of inhibition zone = 11 mm
	*Staphylococcus aureus* ATCC25923:		*Staphylococcus aureus* ATCC25923:
	Diameter of inhibition zone = 17 mm		Diameter of inhibition zone = 10 mm
	*Micrococcus luteus*:		*Micrococcus luteus*:
	Diameter of inhibition zone = 30 mm		Diameter of inhibition zone = 29 mm
	*Escherichia coli* ATCC25922:		*Escherichia coli* ATCC25922:
	Diameter of inhibition zone = 16 mm		Diameter of inhibition zone = 13 mm
	*Pseudomonas aeruginosa* ATCC7853:		*Pseudomonas aeruginosa* ATCC7853:
	Diameter of inhibition zone = 15 mm		Diameter of inhibition zone = 13 mm
	*Proteus vulgaris* RCMB 010085:		*Proteus vulgaris* RCMB 010085:
	Diameter of inhibition zone = 17 mm		Diameter of inhibition zone = 12 mm
	*Candida albicans*:		*Candida albicans*:
	Diameter of inhibition zone = 12 mm		Diameter of inhibition zone = 15 mm
Reference	119		124
Compounds	177		178
Structure	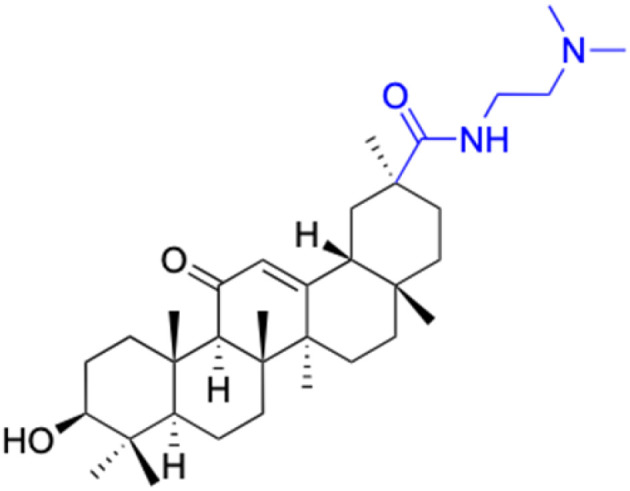		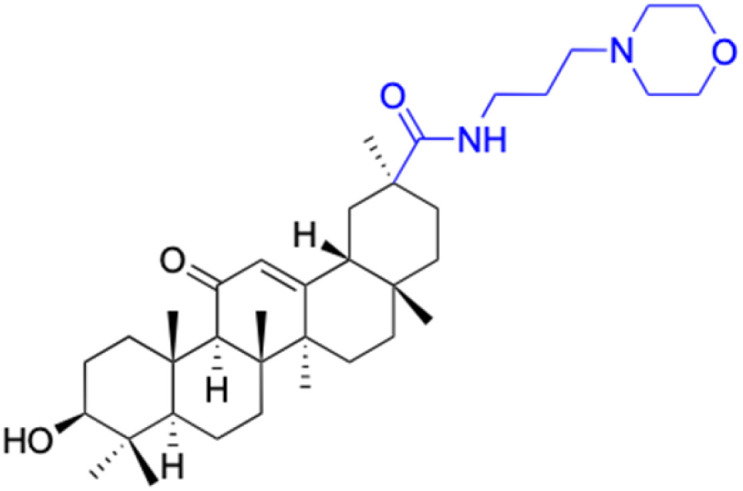
Effects or mechanisms	177:		178:
	Xoo: EC_50_ = 5.89 μg mL^−1^		Xoo: EC_50_ = 36.5 μg mL^−1^
	Psa: EC_50_ = 16.1 μg mL^−1^		Psa: EC_50_ = 114 μg mL^−1^
	Xac: EC_50_ = 3.64 μg mL^−1^		Xac: EC_50_ = 29.1 μg mL^−1^
Reference	119		119
Compounds	179–184		185–187
Structure	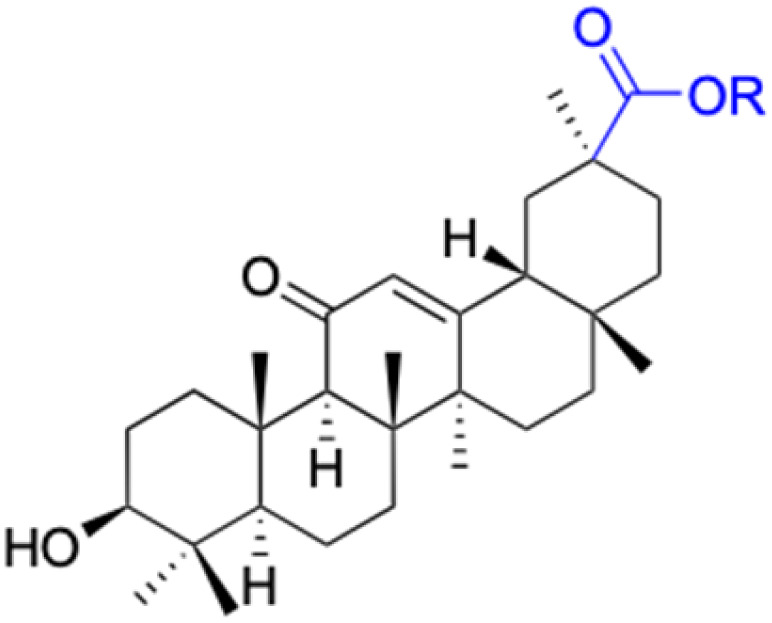		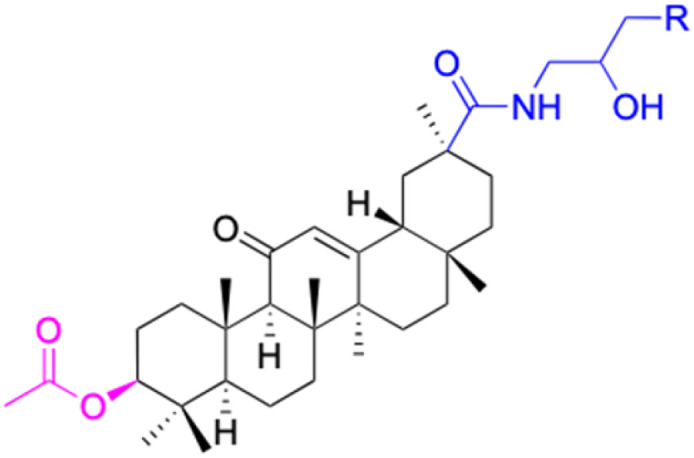
Effects or mechanisms	179:		185: R =
	R = Bn		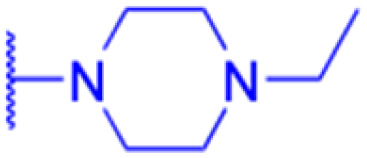
	*Theileria annulata* (T339): GI_50_ = 7.431 μmol L^−1^		Xoo: EC_50_ = 4.69 μg mL^−1^, Xac: EC_50_ = 6.29 μg mL^−1^
	*Theileria annulata* (T5815): GI_50_ = 7.595 μmol L^−1^		186: R =
	180:		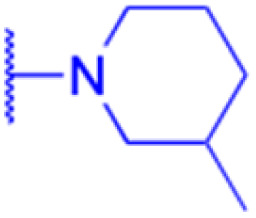
	R = CH_2_CH_3_		Xoo: EC_50_ = 3.64 μg mL^−1^, Xac: EC_50_ = 20.5 μg mL^−1^
	*Theileria annulata* (T339): GI_50_ = 5.638 μmol L^−1^		187: R =
	*Theileria annulata* (T5815): GI_50_ = 7.557 μmol L^−1^		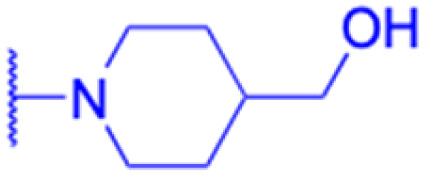
	181:		Xoo: EC_50_ = 5.56 μg mL^−1^, Xac: EC_50_ = 8.83 μg mL^−1^
	R = CH_3_		
	*Theileria annulata* (T5815): GI_50_ = 5.977 μmol L^−1^		
	182:		
	R = CH_2_CH_2_CH_3_		
	*Theileria annulata* (T339): GI_50_ = 5.549 μmol L^−1^		
	*Theileria annulata* (T5815): GI_50_ = 3.55 μmol L^−1^		
	183:		
	R = CH(CH_3_)_2_		
	*Theileria annulata* (T339): GI_50_ = 1.638 μmol L^−1^		
	*Theileria annulata* (T5815): GI_50_ = 1.499 μmol L^−1^		
	184:		
	R = (CH_2_)_3_CH_3_		
	*Theileria annulata* (T5815): GI_50_ = 9.946 μmol L^−1^		
Reference	124		120
Compounds	188–189		190–195
Structure	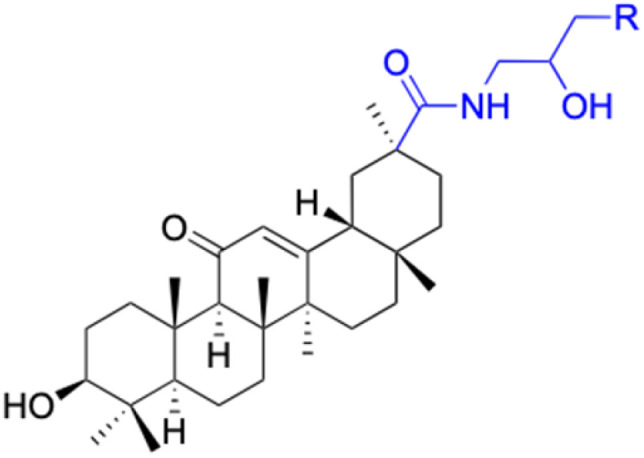		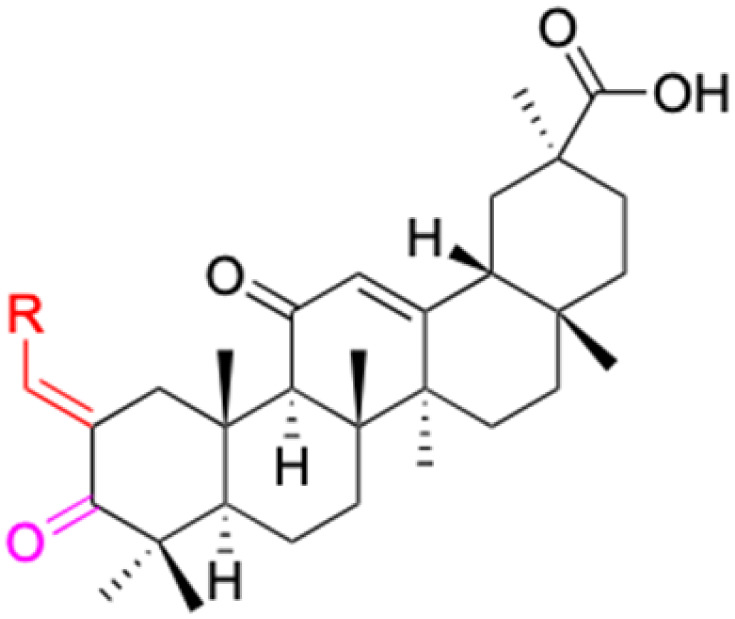
Effects or mechanisms	188: R =		190: R = 5-fluoro-2-nitro-benzene
	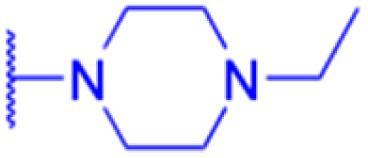		*Staphylococcus aureus* (ATCC 6538): MIC = 10 μmol L^−1^
	Xoo: EC_50_ = 10.2 μg mL^−1^		*Staphylococcus aureus* (ATCC 29213): MIC = 10 μmol L^−1^
	Xac: EC_50_ = 4.16 μg mL^−1^		*Staphylococcus epidermidis* (ATCC 12228): MIC = 10 μmol L^−1^
	189: R =		MRSA: MIC = 16 μmol L^−1^
	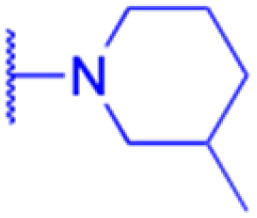		191: R = 4-chloro-2-nitro-benzene
	Xoo: EC_50_ = 10.9 μg mL^−1^		*Staphylococcus aureus* (ATCC 6538): MIC = 10 μmol L^−1^
	Xac: EC_50_ = 5.16 μg mL^−1^		*Staphylococcus aureus* (ATCC 29213): MIC = 5 μmol L^−1^
			*Staphylococcus epidermidis* (ATCC 12228): MIC = 5 μmol L^−1^
			MRSA: MIC = 8 μmol L^−1^
			192: R = 4-methoxy-2-nitro-benzene
			*Staphylococcus aureus* (ATCC 6538): MIC = 5 μmol L^−1^
			*Staphylococcus aureus* (ATCC 29213): MIC = 5 μmol L^−1^
			*Staphylococcus epidermidis* (ATCC 12228): MIC = 5 μmol L^−1^
			MRSA: MIC = 4 μmol L^−1^
			193: R = 5-bromo-2-nitro-benzene
			*Staphylococcus aureus* (ATCC 6538): MIC = 2.5 μmol L^−1^
			*Staphylococcus aureus* (ATCC 29213): MIC = 2.5 μmol L^−1^
			*Staphylococcus epidermidis* (ATCC 12228):MIC = 2.5 μmol L^−1^
			MRSA: MIC = 16 μmol L^−1^
			194: R = 4-bromo-2-nitro-benzene
			*Staphylococcus aureus* (ATCC 6538): MIC = 12.5 μmol L^−1^
			*Staphylococcus aureus* (ATCC 29213):MIC = 12.5 μmol L^−1^
			*Staphylococcus epidermidis* (ATCC 12228):MIC = 12.5 μmol L^−1^
			MRSA: MIC = 16 μmol L^−1^
			195: R = 4-fluoro-2-nitro-benzene
			*Staphylococcus aureus* (ATCC 6538): MIC = 5 μmol L^−1^
			*Staphylococcus aureus* (ATCC 29213): MIC = 5 μmol L^−1^
			*Staphylococcus epidermidis* (ATCC 12228): MIC = 5 μmol L^−1^
			MRSA: MIC = 8 μmol L^−1^
Reference	120		49
Compounds	196–201		202–225
Structure	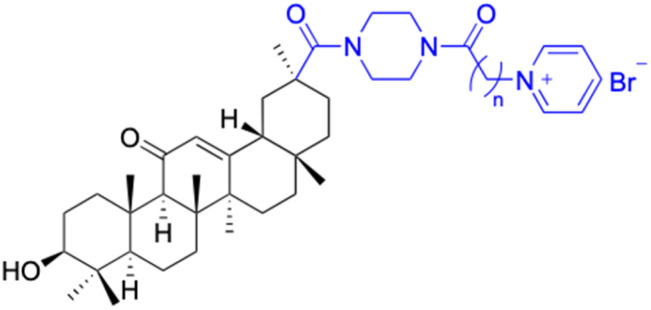		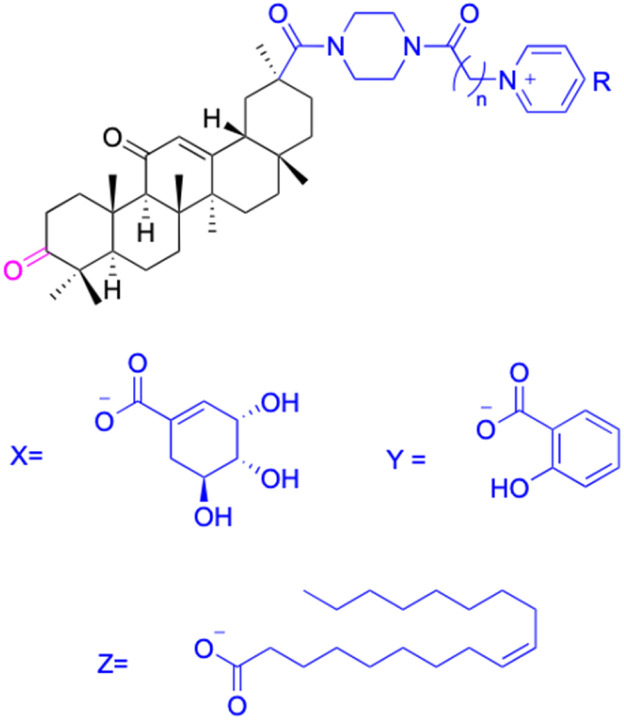
Effects or mechanisms	196:		202:
	*n* = 5		*n* = 5; R = Br^−^
	Xoo: EC_50_ = 8.57 μg mL^−1^, Xac: EC_50_ = 7.67 μg mL^−1^		Xoo: EC_50_ = 9.47 μg mL^−1^, Xac: EC_50_ = 11.8 μg mL^−1^
	195:		203:
	*n* = 6		*n* = 6; R = Br^−^
	Xoo: EC_50_ = 5.24 μg mL^−1^, Xac: EC_50_ = 9.55 μg mL^−1^		Xoo: EC_50_ = 9.18 μg mL^−1^, Xac: EC_50_ = 34.5 μg mL^−1^
	197:		204:
	*n* = 7		*n* = 7; R = Br^−^
	Xoo: EC_50_ = 5.06 μg mL^−1^, Xac: EC_50_ = 8.16 μg mL^−1^		Xoo: EC_50_ = 7.12 μg mL^−1^, Xac: EC_50_ = 9.53 μg mL^−1^
	198:		205:
	*n* = 8		*n* = 8; R = Br^−^
	Xoo: EC_50_ = 3.54 μg mL^−1^, Xac: EC_50_ = 10.3 μg mL^−1^		Xoo: EC_50_ = 3.38 μg mL^−1^, Xac: EC_50_ = 18.7 μg mL^−1^
	199:		206:
	*n* = 9		*n* = 9; R = Br^−^
	Xoo: EC_50_ = 3.47 μg mL^−1^, Xac: EC_50_ = 34.1 μg mL^−1^		Xoo: EC_50_ = 2.29 μg mL^−1^, Xac: EC_50_ = 25.6 μg mL^−1^
	200:		207:
	*n* = 10		*n* = 10; R = Br^−^
	Xoo: EC_50_ = 6.60 μg mL^−1^, Xac: EC_50_ = 17.4 μg mL^−1^		Xoo: EC_50_ = 1.37 μg mL^−1^, Xac: EC_50_ = 37.4 μg mL^−1^
			208:
			*n* = 5; R = X
			Xoo: EC_50_ = 14.08 μg mL^−1^, Xac: EC_50_ = 14.76 μg mL^−1^
			209:
			*n* = 5; R = Y
			Xoo: EC_50_ = 19.53 μg mL^−1^, Xac: EC_50_ = 6.8 μg mL^−1^
			210:
			*n* = 5; R = Z
			Xoo: EC_50_ = 19.06 μg mL^−1^, Xac: EC_50_ = 4.59 μg mL^−1^
			211:
			*n* = 6; R = X
			Xoo: EC_50_ = 12.11 μg mL^−1^, Xac: EC_50_ = 6.88 μg mL^−1^
			212:
			*n* = 6; R = Y
			Xoo: EC_50_ = 12.9 μg mL^−1^, Xac: EC_50_ = 25.03 μg mL^−1^
			213:
			*n* = 6; R = Z
			Xoo: EC_50_ = 20.59 μg mL^−1^, Xac: EC_50_ = 14.81 μg mL^−1^
			214:
			*n* = 7; R = X
			Xoo: EC_50_ = 6.5 μg mL^−1^, Xac: EC_50_ = 14.81 μg mL^−1^
			215:
			*n* = 7; R = Y
			Xoo: EC_50_ = 6.17 μg mL^−1^, Xac: EC_50_ = 11.69 μg mL^−1^
			216:
			*n* = 7; R = Z
			Xoo: EC_50_ = 17.25 μg mL^−1^, Xac: EC_50_ = 14.39 μg mL^−1^
			217:
			*n* = 8; R = X
			Xoo: EC_50_ = 5.17 μg mL^−1^, Xac: EC_50_ = 7.16 μg mL^−1^
			218:
			*n* = 9; R = X
			Xoo: EC_50_ = 4.18 μg mL^−1^, Xac: EC_50_ = 10.32 μg mL^−1^
			219:
			*n* = 10; R = X
			Xoo: EC_50_ = 1.6 μg mL^−1^, Xac: EC_50_ = 8.48 μg mL^−1^
			220:
			*n* = 8; R = Y
			Xoo: EC_50_ = 4.93 μg mL^−1^, Xac: EC_50_ = 3.82 μg mL^−1^
			221:
			*n* = 9; R = Y
			Xoo: EC_50_ = 7.56 μg mL^−1^, Xac: EC_50_ = 4.38 μg mL^−1^
			222:
			*n* = 10; R = Y
			Xoo: EC_50_ = 4.14 μg mL^−1^, Xac: EC_50_ = 10.15 μg mL^−1^
			223:
			*n* = 8; R = Z
			Xoo: EC_50_ = 13.77 μg mL^−1^, Xac: EC_50_ = 22.17 μg mL^−1^
			224:
			*n* = 9; R = Z
			Xoo: EC_50_ = 12.46 μg mL^−1^, Xac: EC_50_ = 2.07 μg mL^−1^
			225:
			*n* = 10; R = Z
			Xoo: EC_50_ = 2.98 μg mL^−1^, Xac: EC_50_ = 6.08 μg mL^−1^
Reference	121		121
Compounds	226		
Structure	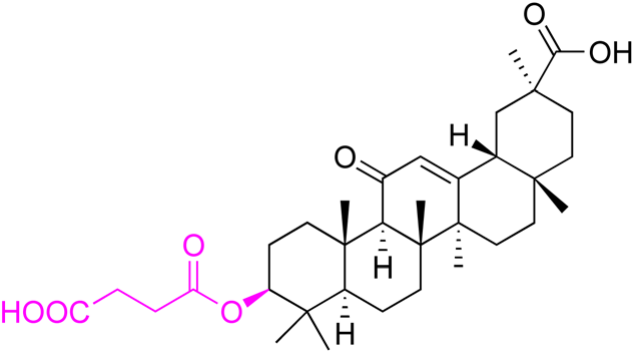		
Effects or mechanisms	226:		
	MRSA SA5002: MIC = 16 mg L^−1^		
	MRSA SA5053: MIC = 16 mg L^−1^		
	MSSA SA5028: MIC = 16 mg L^−1^		
Reference	122		
Abbreviations	Xac: *Xanthomonas citri* subsp. *citri*. Xoo: *Xanthomonas oryzae* pv. *oryzae*. Psa: *Pseudomonas syringae* pv. actinidiae. MRSA: methicillin-resistant *Staphylococcus aureus*		

Compounds 157–163 have emerged as potent inhibitors of *Streptomyces scabies*, a notorious plant pathogen. These derivatives have exhibited remarkable inhibitory activity, suggesting their potential application in managing plant bacterial diseases.^117^ Compound 161 has demonstrated superior inhibitory activity against *Bacillus subtilis*, *Staphylococcus aureus*, and MRAS compared to conventional antibiotics such as ampicillin, streptomycin, and vancomycin. This finding highlights the potential of 18β-GA derivatives as effective alternatives for combating drug-resistant bacterial strains.

Furthermore, compounds 164–166, compounds 177–178, compounds 183–187, and compounds 196–225 have displayed robust inhibitory activity against *Xanthomonas oryzae* pv. *oryzae* (Xoo) and *X. axonopodis* pv. *citri* (Xac).^118–121^ Xiang *et al.* particularly emphasized the potency of compounds 164 and 165. *In vivo* trials have further confirmed the potential of these compounds in managing rice bacterial blight disease, with control efficacy ranging between 50.57% and 53.70% at 200 μg mL^−1^.^118^

Moreover, Yang *et al.* discovered that derivatives of 18β-GA (compounds 167–176, 190–195, and 226) exhibit potent antibacterial activity against *Staphylococcus aureus*, *Staphylococcus epidermidis*, and MRAS.^43,122^ Compound 172, as identified by Guo *et al.*, has demonstrated robust antibacterial properties and has been used to prepare supramolecular self-assembly hydrogels with exceptional thermodynamic stability and high melting temperatures.^123^ Additionally, compounds 173–176 have exhibited high activity against various bacteria, particularly showing enhanced antibacterial effects against *Micrococcus luteus* compared to gentamicins.^79^

Tropical bovine theileriosis (TBT) is one of the progressive and lymphoproliferative tick-borne diseases caused by *Theileria annulata*. Buvanesvaragurunathan *et al.* investigated the effect of 18β-GA esters (compounds 179–184) on the growth of *Theileria annulata* and found that they induced apoptosis in parasite cells. Among these esters, the isopropyl ester of 18β-GA (compound 183) showed improved anti-theileriosis efficacy than other 18β-GA derivatives.^124^

In conclusion, the rise of drug-resistant bacteria necessitates the discovery of novel antibiotics that can effectively combat these resilient strains mentioned above. Natural compounds, such as 18β-GA and its derivatives, offer a promising avenue for antibiotic development. Future research efforts should focus on understanding the mode of action of these compounds and optimizing their efficacy against drug-resistant bacteria.

## Antiviral activity

Over the past two decades, the potencies have been extensively investigated for pentacyclic triterpenoids, such as asiatic acid, betulinic acid, boswellic acid, glycyrrhizin, 18β-GA, lupeol, oleanolic acid, and ursolic acid, and their analogs and derivatives, as potent antitumor and antiviral agents. These triterpenoids have displayed remarkable cytotoxic activity against various tumor cell lines and exhibit antiviral properties, in particular, anti-HIV activity.^126^ The main active constituents of licorice are triterpenoids, which have shown inhibitory effects on several viruses, including SARS-CoV-2.^127^ It has been revealed that these compounds achieve their antiviral effects through various mechanisms such as inhibiting virus replication, directly inactivating viruses, halting inflammation mediated by HMGB1/TLR4, preventing β-chemokines, reducing the binding of HMGB1 to DNA to weaken virus activity, and inhibiting reactive oxygen species formation.^128,129^ While these natural products offer great potential as anti-viral and anti-microbial agents, they comprise complex mixtures of organic molecules, making it difficult to determine their exact effectiveness. Hence, further research is required to gain an intricate understanding of their mechanisms of action and their potential for use as food or herbal medicine. Additionally, it is vital to carefully consider the pleiotropic effects of these compounds to avoid potential negative consequences.

**Fig. 5:**
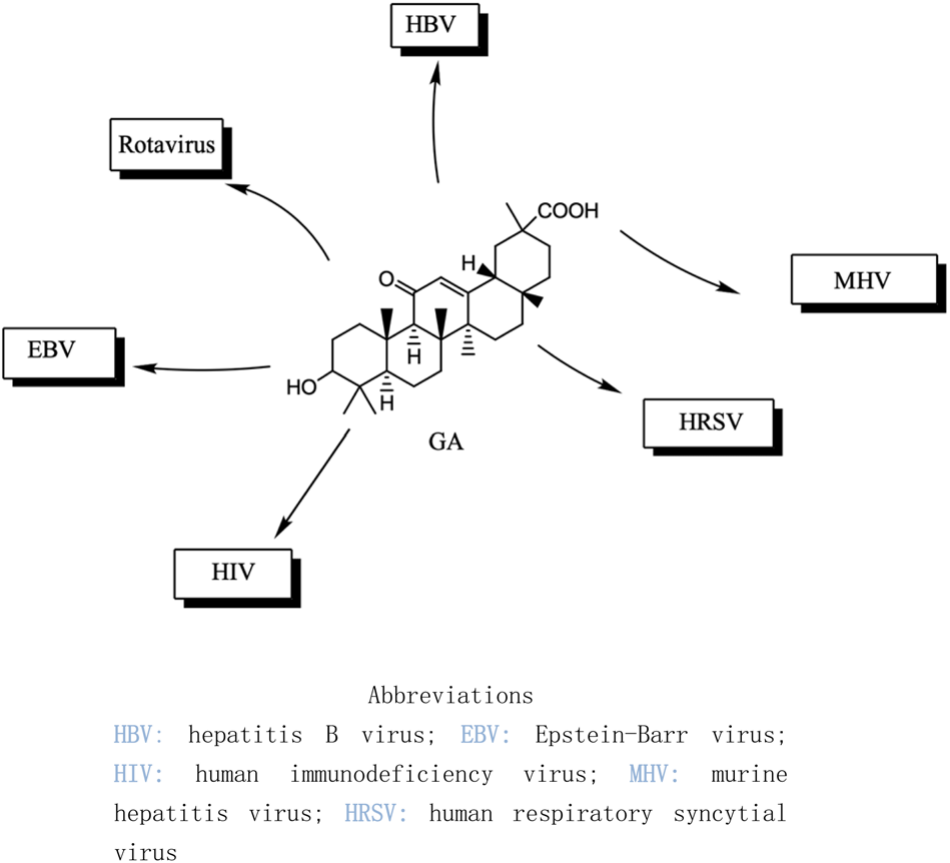
The effect of 18β-GA on antiviral.

Several studies have shown that 18β-GA inhibit several viruses (Fig. 5), for example, Sato *et al.* reported that 18β-GA inhibits *hepatitis B virus* (HBV) by suppressing surface antigens,^130^ while Hardy *et al.* showed that 18β-GA exhibits significant antiviral activity against rotavirus replication *in vitro*.^131^ Other investigations demonstrated that 18β-GA inhibited rotavirus SA11 *via* the Fas/FasL pathway, inhibits *Epstein–Barr virus* (EBV) in superinfected Raji cells, showed significant antiviral activity against human immunodeficiency virus (HIV), inhibits infection of *human respiratory syncytial virus* (HRSV), and significantly protects against *murine hepatitis virus* (MHV)-induced severe hepatic injury by suppressing HMGB1 release.^35,132–135^

In recent years, researchers have also worked on the antiviral properties of 18β-GA derivatives (Table 4). Baltina *et al.* synthesized a series of 18β-GA derivatives. They found that compounds 227–230 exert the most significant antiviral activity (IC_50_ = 0.13 μM) against ZIKV, with compound 227 demonstrating promising potential as an antiviral agent against ZIKV infection.^136^ Similarly, Zígolo *et al.* reported that compound 231 exhibited significant antiviral activity against TK+ and TK− strains of *herpes simplex* virus type 1 (HSV-1).^137^ Liang *et al.* found that water-soluble β-cyclodextrin-18β-GA (compounds 232–237) showed promising antiviral activity against the influenza A/WSN/33 (H1N1) virus.^138,139^ More recently, Ding *et al.* suggested that 18β-GA and its derivatives (compounds 238–241) could alleviate the symptoms of COVID-19 patients.^140^ Additionally, Wang *et al.* synthesized several compounds and observed that compounds 242–243 exhibited significant inhibitory activities against HBV DNA replication.^73^ These findings highlight the potential of 18β-GA and its derivatives as potent antiviral agents with remarkable antiviral activity against numerous viral infections.

**Table 4:** Chemical structure and antiviral activity of 230–246

Compounds	227–228	229–230	231
Structure	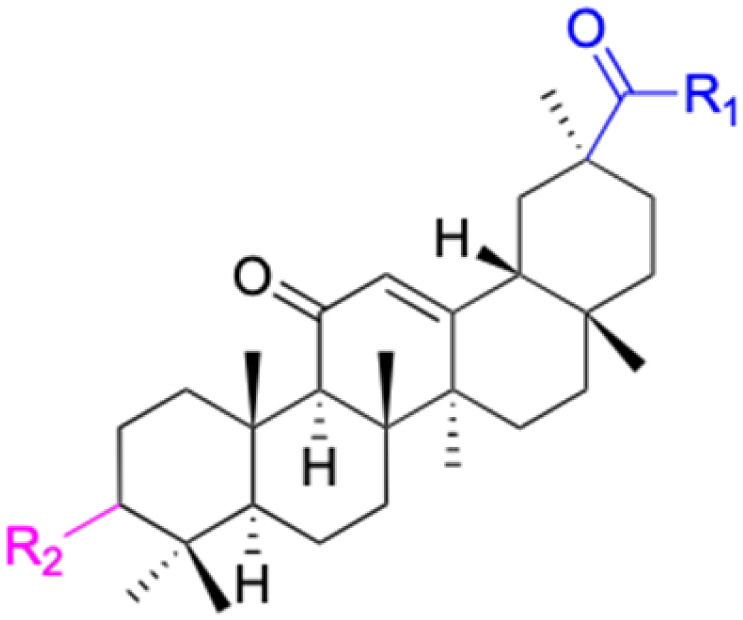	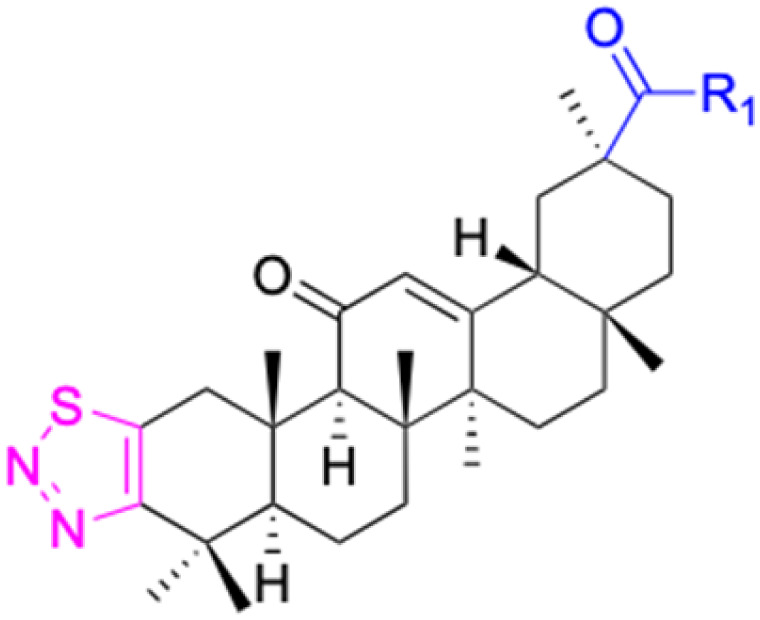	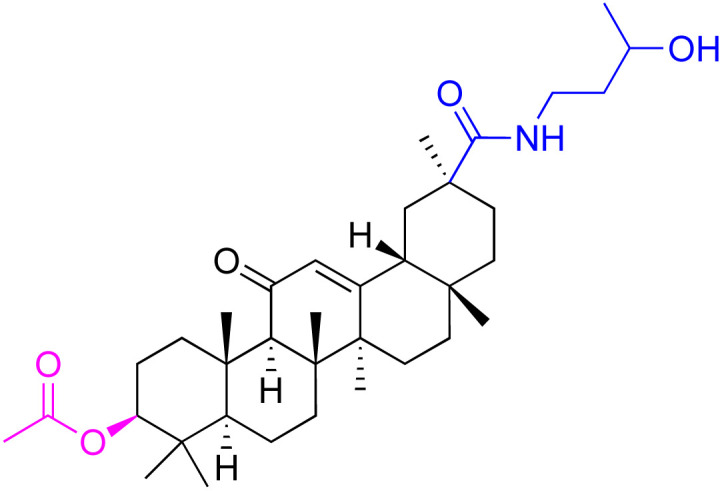
Effects or mechanisms	227:	229:	231:
	R_2_ = OAc	R_1_ = COOBu	HSV-1 virus: CC_50_ = 190.2 μM, EC_50_ = 4.95 μM, CC_50_/EC_50_ = 38.38
	R_2_ =	ZIKA virus: CC_50_ > 50 μM, IC_50_ = 0.29 μM, CC_50_/IC_50_ > 172.4	
	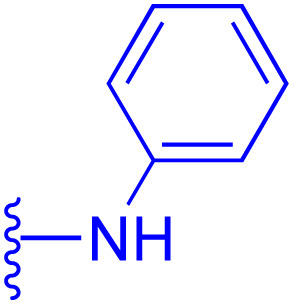	230:	
	ZIKA virus: CC_50_ > 50 μM	R_1_ = COOCH_3_	
	IC_50_ = 0.13 μM, CC_50_/IC_50_ > 384	ZIKA virus: CC_50_ > 50 μM, IC_50_ = 0.56 μM	
	228:	CC_50_/IC_50_ > 89.3	
	R_2_ =		
	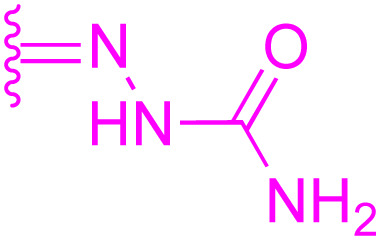		
	R_1_ = COOBu		
	ZIKA virus: CC_50_ > 50 μM		
	IC_50_ = 0.55 μM, CC_50_/IC_50_ > 90.9		
Reference	136	136	137
Compounds	232–237	238–241	
Structure	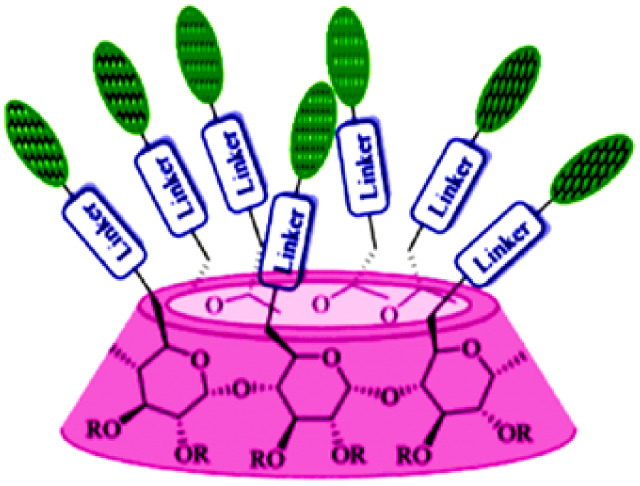	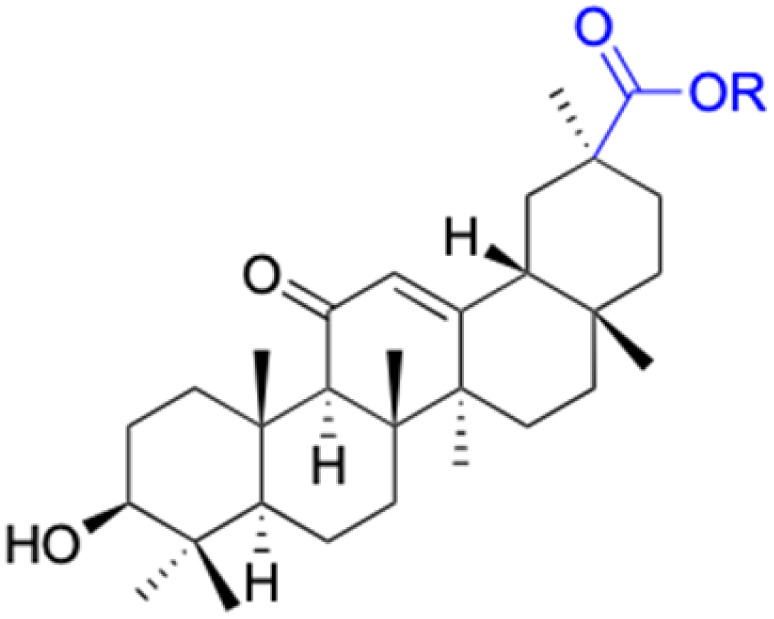	
	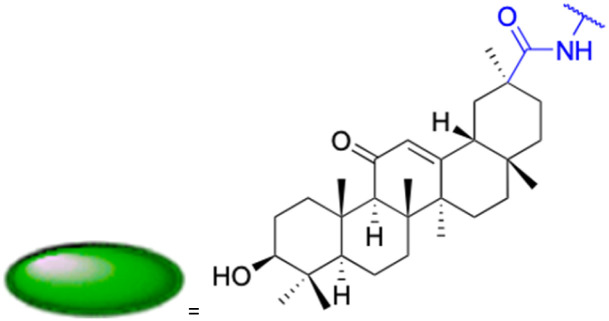		
Effects or mechanisms	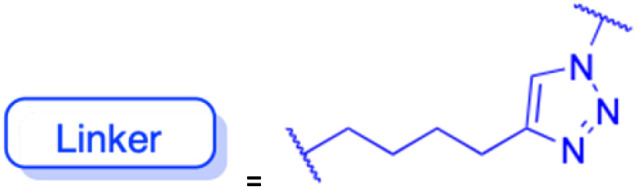	238:	
	232:	R =	
	R = Ac	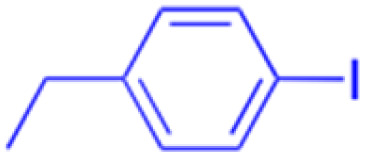	
	Influenza A/WSN/33 (H1N1) virus: IC_50_ = 12.1 μM, CC_50_ > 100 μM, SI > 8.3	HBV: CC_50_ > 985.68 μM, IC_50_ = 5.71 μM, SI > 172.6	
	233:	239:	
	R = H	R =	
	Influenza A/WSN/33 (H1N1) virus: IC_50_ = 9.03 μM, CC_50_ > 100 μM, SI > 11.1	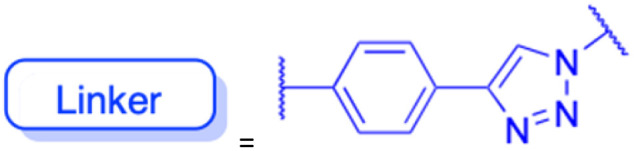	
	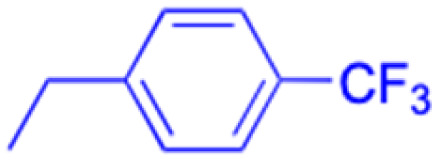	HBV: CC_50_ > 1373.13 μM, IC_50_ = 5.36 μM, SI > 255.9	
	234:	240:	
	R = Ac	R =	
	Influenza A/WSN/33 (H1N1) virus: IC_50_ = 20.7 μM, CC_50_ > 100 μM, SI > 4.8	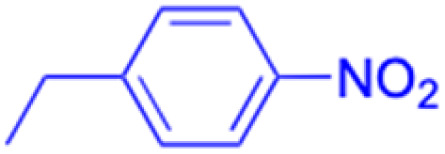	
	235:	HBV: CC_50_ > 1327.92 μM, IC_50_: 8.90 μM, SI > 149.2	
	R = H	241:	
	Influenza A/WSN/33 (H1N1) virus: IC_50_ = 11.0 μM, CC_50_ > 100 μM, SI > 9.1	R =	
	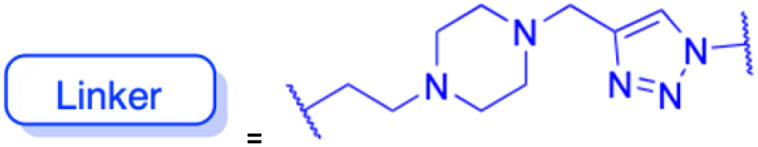	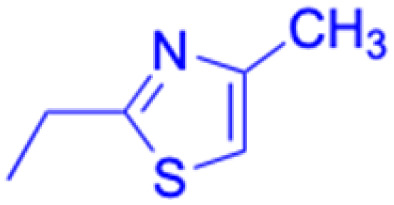	
	236:	HBV: CC_50_ = 37.17 μM, IC_50_ = 9.08 μM, SI = 4.1	
	R = Ac		
	Influenza A/WSN/33 (H1N1) virus:IC_50_ = 20.7 μM, CC_50_ > 100 μM, SI > 4.8		
	237:		
	R = H		
	Influenza A/WSN/33 (H1N1) virus: IC_50_ = 11.0 μM, CC_50_ > 100 μM, SI > 9.1		
Reference	138 and 139	140	
Compounds	242	243	
Structure	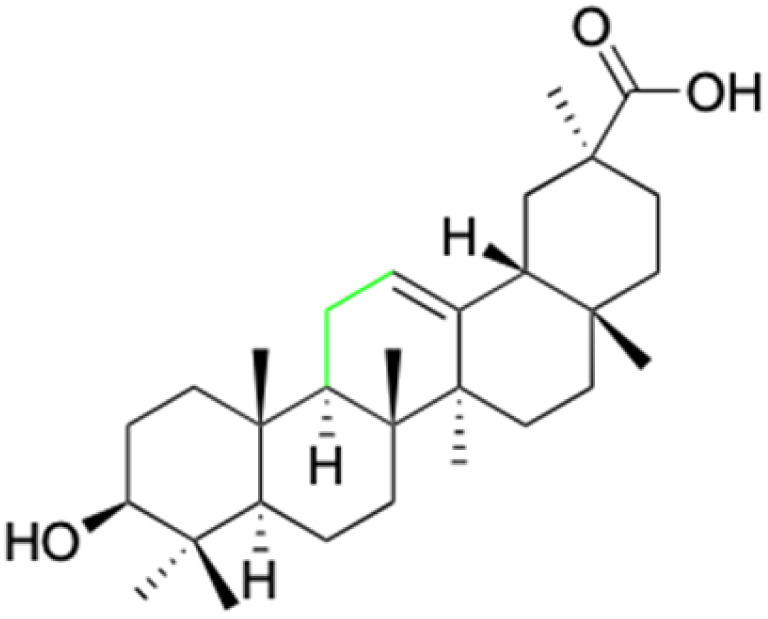	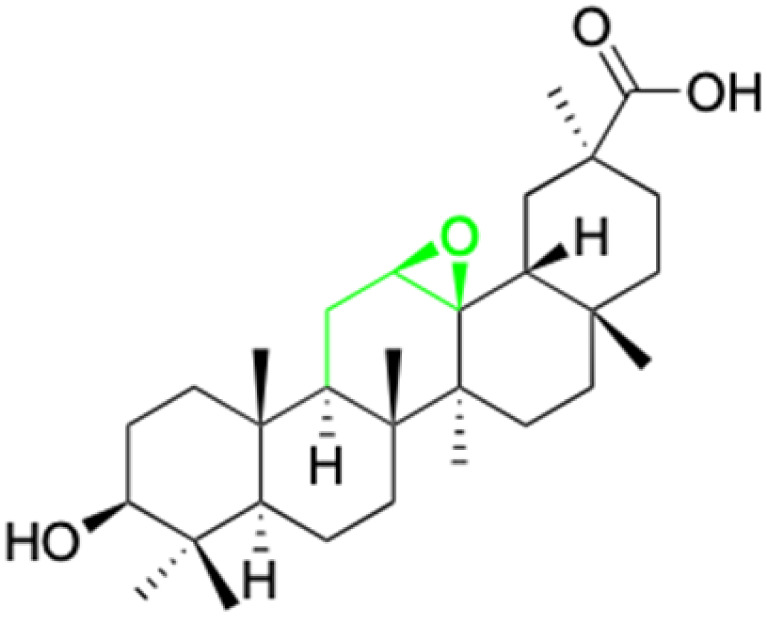	
Effects or mechanisms	242:	243:	
	HBV:	HBV:	
	CC_50_ = 161.68 μM	CC_50_ = 35.71 μM	
	IC_50_ = 47.00 μM	IC_50_ = 18.37 μM	
	SI = 3.4	SI = 1.9	
Reference	73	73	

In summary, the research on pentacyclic triterpenoids, including 18β-GA and its derivatives, suggests their immense potential as effective and safe antiviral agents. These compounds have demonstrated varying degrees of antiviral activity against numerous viral infections, making them a promising area of ongoing research. However, further studies are necessary to comprehensively investigate their mechanisms of action and how they can be effectively used as food or herbal medicine while considering the possible negative consequences of their pleiotropic effects.

## Antioxidant activity

18β-GA has been found to exhibit significant antioxidant activity, which makes it of great interest in the research of antioxidants. Alanazi *et al.* found that the serum concentrations of final glucose, aspartate aminotransferase (AST), alanine aminotransferase (ALT), and alkaline phosphatase (ALP) in mice treated with 20 mg per kg acrylamide (Acr) increased to 131 ± 12.2 mg dL^−1^, 76.5 ± 12.0 μ U^−1^, 47.7 ± 9.17 μ L^−1^, and 82.5 ± 10.3 μ L^−1^, which is much higher than the normal concentrations (serum final glucose, AST, ALT, and alkaline ALP concentrations of 87.7 ± 5.93 mg dL^−1^, 21.1 ± 2.60 μ U^−1^, 10.7 ± 1.16 μ L^−1^, and 24.1 ± 3.97 μ L^−1^), respectively, compared to these serums in the 18β-GA-Acr (50 mg per kg 18β-GA) group. The biochemical variables of rats return to normal. The findings provide sufficient evidence to demonstrate that 18β-GA possesses the capability to suppress the production of oxygen species and reinstate the antioxidant mechanisms in diabetic rats afflicted with acrylamide-induced liver and kidney cytotoxicity.^141^ Similarly, Melekoglu *et al.* discovered that the antioxidant defense system parameters, encompassing malondialdehyde (MDA), reduced glutathione (GSH), superoxide dismutase (SOD), and catalase (CAT), were significantly higher in the ovarian tissues of rats treated with 18β-GA (100 mg kg^−1^ day^−1^) compared to those subjected to ischemia-reperfusion (I/R) alone.^142^ These findings suggest that 18β-GA may have protective effects against oxidative stress in a variety of tissues and systems. In addition to its potential antioxidant properties, recent research has also explored the potential therapeutic applications of 18β-GA in the context of viral infections. For example, Rehman *et al.* found that 18β-GA exhibited a solid binding affinity for several SARS-CoV-2 protein targets, including main protease (binding energy = −9.46 kcal mol^−1^), helicase (binding energy = −9.91 kcal mol^−1^), spike glycoprotein (S) (binding energy = −8.08 kcal mol^−1^), and E-channel proteins (binding energy = −9.72 kcal mol^−1^), through ligand–protein interactions. This finding suggests that 18β-GA may have the potential as a therapeutic agent in the fight against COVID-19.^143^

**Fig. 6:**
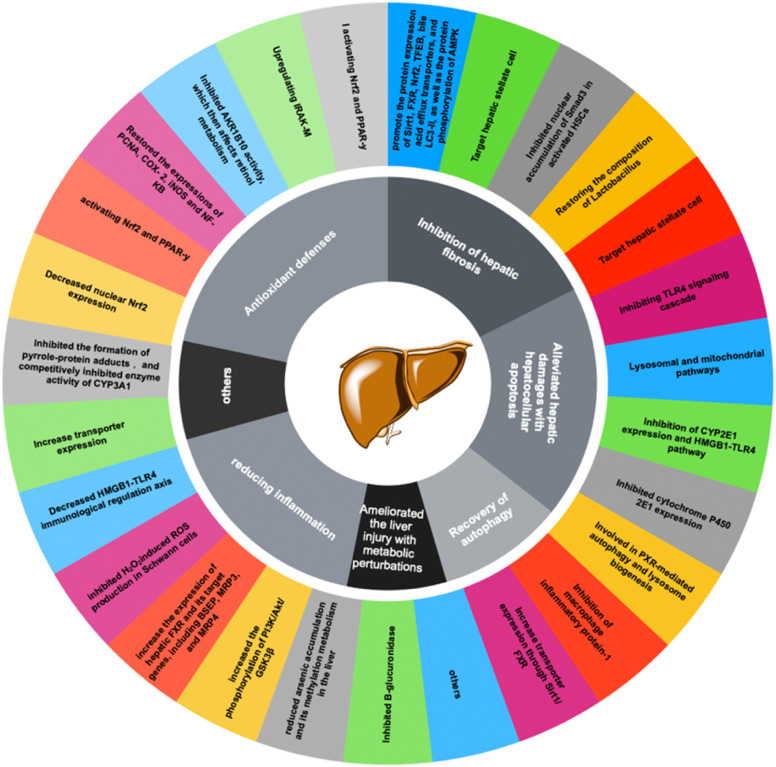
Mechanism of hepatoprotective effect of glycyrrhetinic acid.

We have discovered that a significant number of studies on the antioxidant properties of 18β-GA focus on its hepatoprotective function. In the mouse model of carbon tetrachloride (CCl_4_)-induced chronic liver fibrosis, it was observed that CCl_4_ inhibited the expression of Nrf2 regulatory genes, including CAT, glutathione peroxidase 2 (GPX2), and superoxide dismutase 3 (SOD3). However, 18β-GA was found to protect the mouse liver from oxidative stress by potentially activating the nuclear trans of Nrf2, enhancing the expression of its target genes, and increasing the activity of antioxidant enzymes.^37^ Furthermore, 18β-GA was also found to have the ability to inhibit the activity of xanthine oxidase (XO) significantly. XO is responsible for reducing O_2_ to superoxide anionic radical O_2_, leading to oxidative stress.^144^ In a mouse model of methotrexate (MTX)-induced liver injury, Mahmoud *et al.* discovered that 18β-GA was able to reverse the significant manifestations of Nrf2, hemooxygenase-1, and PPARg induced by MTX, thus restoring antioxidant defense.^38^ Another study demonstrated that 18β-GA significantly reduced alpha-naphthylisothiocyanate (ANIT)-induced liver damage primarily by increasing the expression of nuclear factors (such as Sirt1, FXR, and Nrf2) and their targeted excretion transporters in the liver, which play a crucial role in maintaining bile acidosis in hepatocytes. The plasma levels of ALT, AST, ALP, γ-glutamyl transpeptidase (GGT), and total bilirubin (TBIL) were significantly elevated by 31.2-, 33.4-, 5.1-, 5.0-, and 91.3-fold, respectively, in rats induced with ANIT (*P* < 0.0001). However, for 18β-GA (60 mg kg^−1^ for 7 days treatment), all of these levels showed a significant reduction of 62.0%, 38.5%, 45.7%, 51.6%, and 39.7%, respectively (*P* < 0.05).^145^ Moreover, the study also revealed that 18β-GA exerts its hepatoprotective effects against RTS-induced liver damage through the phosphatidylinositol 3-kinase (PI3K)/protein kinase B (AKT) pathway and enhanced glycogen synthase kinase 3 beta (GSK3β) pathway, which promotes the Nrf2-mediated antioxidant system.^146^Fig. 6 briefly illustrates the hepatoprotective effect of 18β-GA based on anti-inflammatory and antioxidant mechanisms. Additionally, other hepatoprotective mechanisms are also discussed, such as the inhibition of the TLR/NF-κB pathway and upregulation of hepatic FXR to facilitate bile acid synthesis, transport, and detoxification, competitive inhibition of cyto P450 (CYP) enzymes responsible for the activation of pyrrolizidine alkaloid (PA) metabolism, particularly C3A1, which protects against liver damage, activation of PXR to regulate autophagy and lysosomal biogenesis, thereby alleviating acute liver injury, inhibition of hepatic stellate cell activation, and direct transcriptional inhibition of α2 (I) collagen gene (COL1A2), as observed in transgenic reporter mice, and other mechanisms.^147–150^

18β-GA derivatives (Table 5) also demonstrated significant antioxidant activity. It was discovered that compounds 244–247 exhibited robust antioxidant activity and inhibited ROS activity by up to 41%.^151^ Maitraie *et al.* observed that compounds 249–258 displayed both anti-inflammatory and antioxidant properties, with compound 254 specifically exerting inhibitory effects on NO and superoxide anions in RAW 246.7 cells.^152^ Moreover, Zhang *et al.* found that compounds 259–263 hindered the proliferation of activated hepatic stellate cells (HSC)-T6 cells by inducing apoptosis and arresting them in the G0/G1 phase. They used rat hepatic stellate cell line T6 cells activated by transforming growth factor-β-1 as the cell model and as the 18β-GA control. The IC_50_ value of the compound on cell proliferation was determined by tetrazolium salt colorimetry. It was found that the inhibitory effect of compounds 259–263 on activated HSC-T6 was stronger than that of GA (IC_50_ = 78.4 ± 2.3 μM).^153^ Numerous studies have demonstrated a strong association between COX-2 and the activation of hepatic stellate cells (HSCs), thereby facilitating the initiation and progression of hepatic fibrosis. Among them, compounds 262 and 265 strongly inhibit the activation of HSC-T6 cells by downregulating the expression of alpha-smooth muscle actin (α-SMA) and type I collagen (Col1) proteins, which are biomarkers of liver fibrosis. After treatment with compound activated HSC-T6, the expression levels of the two biomarkers were down-regulated. Second, both compounds downregulated the expression levels of COX-2 and transforming growth factor beta1 (TGF-β_1_) and reduced ROS levels in a concentration-dependent manner. This suggests that they inhibit HSC-T6 activation and may also be due to downregulation of COX-2 levels, inhibition of the TGF-β1 signaling pathway, and reduction of ROS levels.

**Table 5:** Chemical structure and antioxidant activity of derivatives 247–266

Compounds	244	245	246	247
Structure	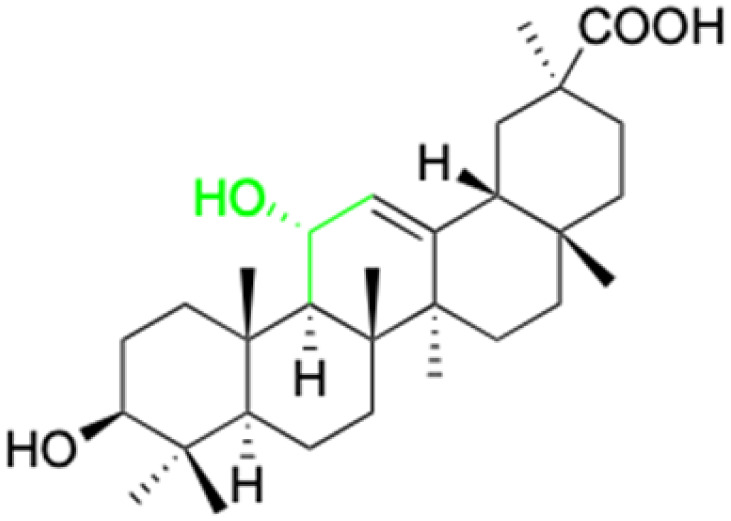	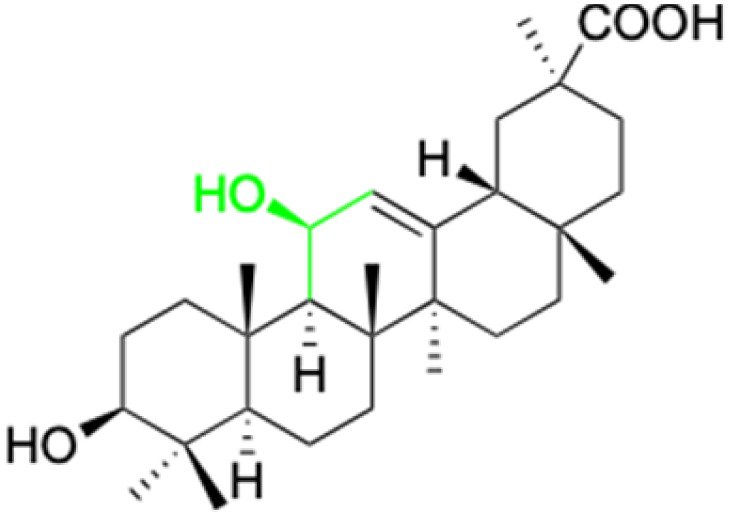	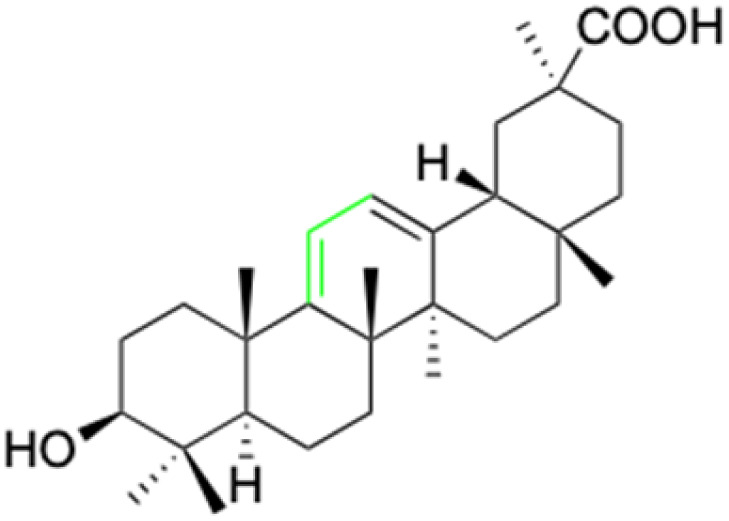	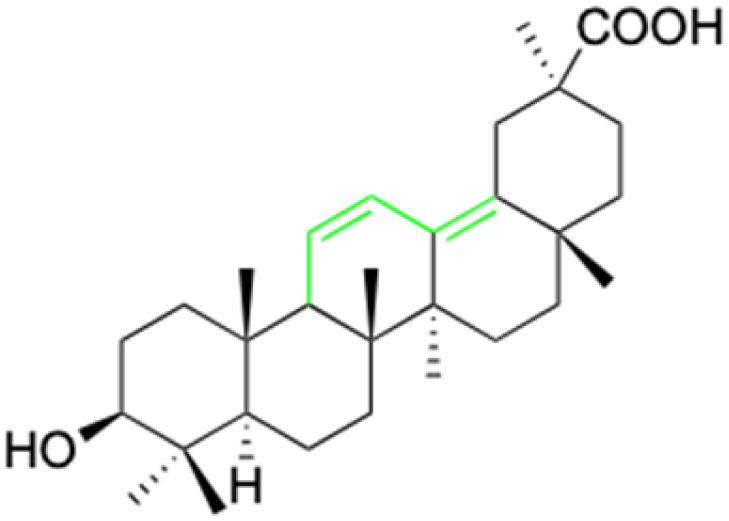
Effects or mechanisms	244:	245:	246:	247:
	ROS:	ROS:	ROS:	ROS:
	Inhibition of 50% activity (1.0 mg mL^−1^)	Inhibition of 51% activity (1.0 mg mL^−1^)	Inhibition of 44% activity (1.0 mg mL^−1^)	Inhibition of 41% activity (1.0 mg mL^−1^)
Reference	151	151	151	151
Compounds	248	249–258		
Structure	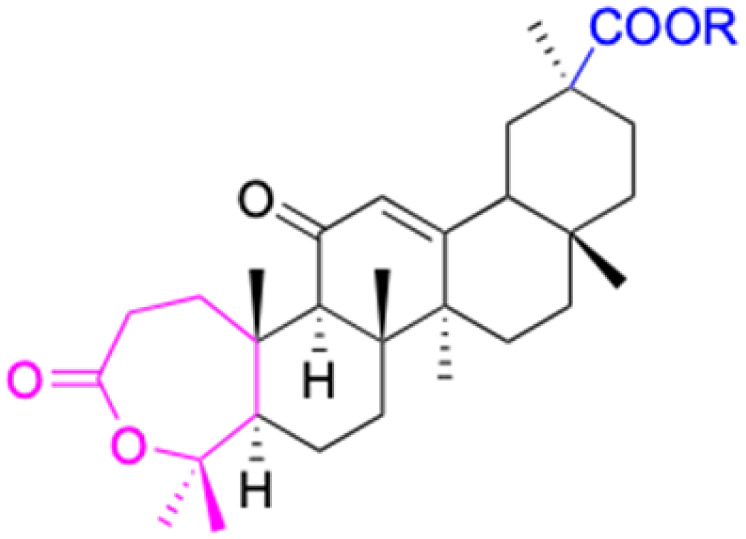	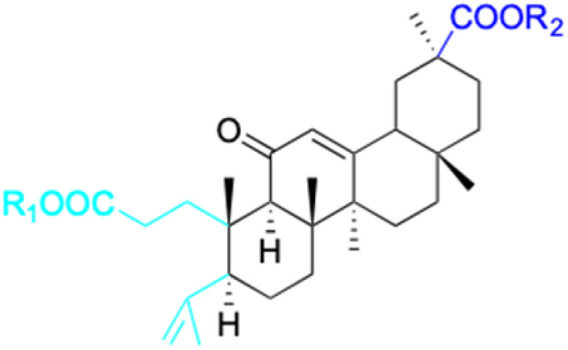		
Effects or mechanisms	248: R = CH_3_	249: R_1_ = H, R_2_ = H		
	PMA: IC_50_ = 12.9 μM	fMLP/CB: IC_50_ = 7.0 μM		
		250: R_1_ = CH_3_, R_2_ = H		
		RAW 264.7 (B): IC_50_ = 26.1 μM		
		251: R_1_ = CH_3_, R_2_ = CH_3_		
		RAW 264.7 (A): IC_50_ = 44.3 μM		
		252: R_1_ = CH_3_, R_2_ = CH(CH_3_)_2_		
		RAW 264.7 (A): IC_50_ = 43.0 μM		
		253: R_1_ = CH_3_, R_2_ = Bn		
		PMA: IC_50_ = 17.0 ± 1.5 μM		
		RAW 264.7 (A): IC_50_ = 44.5 μM		
		RAW 264.7 (B): IC_50_ = 13.7 μM		
		254: R_1_ = CH_3_, R_2_ = CH(CH_3_)_2_		
		PMA: IC_50_ = 15.6 μM		
		RAW 264.7 (A): IC_50_ = 13.1 μM		
		255: R_1_ = CH_3_, R_2_ = C_6_H_5_		
		RAW 264.7 (B): IC_50_ = 15.5 μM		
		256: R_1_ = H, R_2_ = Bn		
		RAW 264.7 (B): IC_50_ = 2.3 μM		
		257: R_1_ = H, R_2_ = CH(CH_3_)_2_		
		fMLP/CB: IC_50_ = 9.8 μM		
		258: R_1_ = H, R_2_ = C_6_H_5_		
		RAW 264.7 (B): IC_50_ = 27.7 μM		
Reference	152	152		
Compounds	259	260		
Structure	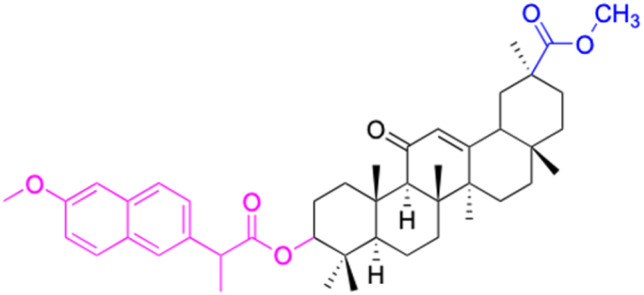	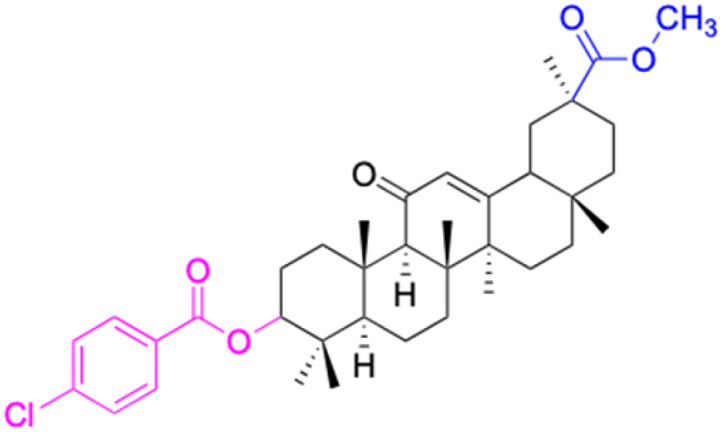		
Effects or mechanisms	259:	260:		
	HSC-T6*: IC_50_ = 17.6 μM	HSC-T6*: IC_50_ = 63.8 μM		
Reference	153	153		
Compounds	261	262		
Structure	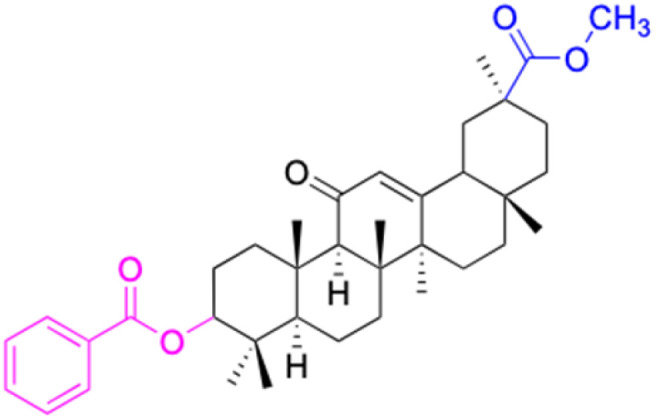	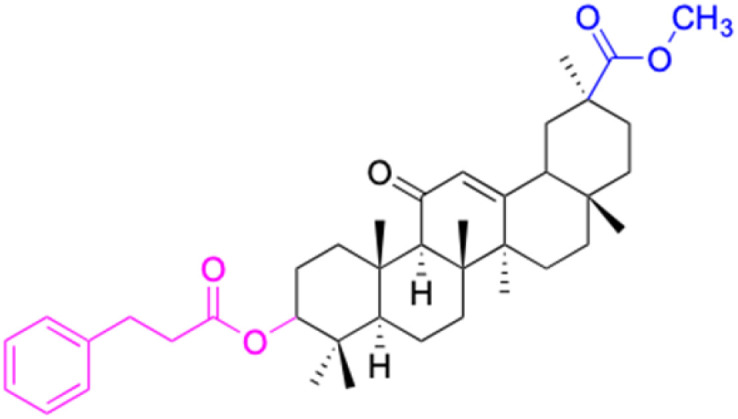		
Effects or mechanisms	261:	262:		
	HSC-T6*: IC_50_ = 54.5 μM	HSC-T6*: IC_50_ = 30.3 μM		
Reference	153	153		
Compounds	263			
Structure	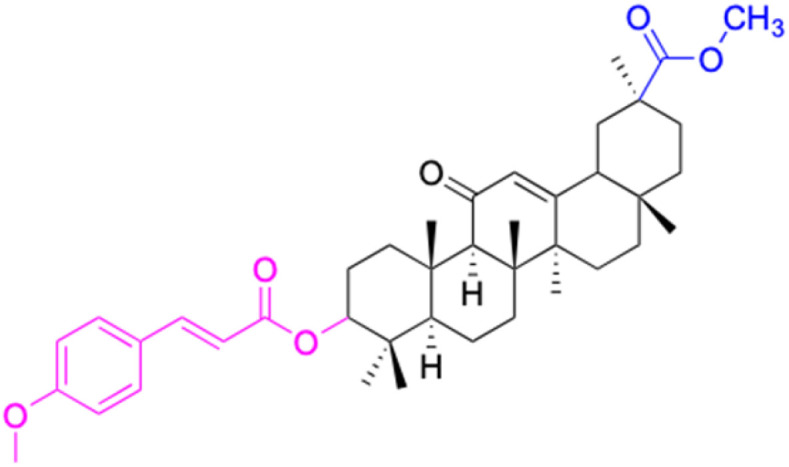			
Effects or mechanisms	263:			
	HSC-T6*: IC_50_ = 59.8 μM			
Reference	153			
Abbreviations	ROS: reactive oxygen species. PMA: superoxide anion formation fromrat neutrophils stimulated with PMA. fMLP/CB: superoxide anion formation from rat neutrophils stimulated with fMLP/CB. RAW 264.7 (A): the accumulation of NO_2_ inRAW 264.7 cells stimulated with LPS. RAW 264.7 (B): TNF-a formation from RAW 264.7 cellsstimulated with LPS. HSC-T6*: HSCs activated by TGF-β1 (10 ng mL^−1^)			

Overall, while the study of oxidative stress and its effects on the body is complex, recent research has shed light on the potential benefits of compounds like 18β-GA in combatting this process. By exploring the mechanisms of these compounds and their effects on various tissues and systems, we can better understand how to combat oxidative stress and its associated health risks.

## Discussion

Experience has imparted the understanding that when a compound manifests a biological activity characterized by an IC_50_ value lower than 10 μM, it may be classified as potential biological efficacy. Additionally, in the process of scrutinizing lead and candidate compounds, it is importance to consider both cost-effectiveness and the intricacy of synthetic routes. Keeping these pivotal factors in consideration, the investigation unveiled that compounds 16–21 exhibited noteworthy inhibitory activities against 11β-HSD2 within the sub-micromolar (nM) range. Particularly remarkable is compound 16, which boasts an exceptionally modest synthetic complexity, necessitating a single-step reaction initiated from 18β-GA. The incorporation of amide and hydroxyl groups at the C-30 position has substantially augmented the solubility of 18β-GA. Compounds of this kind exhibit tremendous promise for further in-depth exploration. Moreover, numerous studies have demonstrated that the majority of structural alterations to 18β-GA revolve around rigid five-ring skeleton structure, encompassing the addition, removal, and replacement of functional groups. Comparatively, few studies explore the strategy, such as scaffold hopping and changes in the skeleton itself to the biological activity. Reports about compounds 38–41, 116–122, 227–230, and 248–259 have discernible indicated that brought about a substantial augmentation in the anti-tumor, antiviral, and antioxidant properties of 18β-GA through the processes of ring opening and ring expansion. The modifications in 18β-GA from the complexity of the derivative structure is mainly due to addition rather than subtraction. It may be connected with that there are few reaction methods for removing carbon atoms in the rigid alkyl skeleton.

It is particularly noteworthy that compounds 227–230 demonstrate an inhibitory activity against the ZIKA virus within the sub-micromolar (nM) range. Perhaps designing modifications that involve adding or reducing rings could provide excellent solutions for enhancing the target binding strength, selectivity, bioavailability, selective tissue distribution, and metabolic stability of 18β-GA derivatives. However, further studies are necessary to comprehensively reveal their mechanisms or the target protein to further guide the modification of compounds. Moreover, 18β-GA derivatives that self-assemble, including gels, micelles, nanoparticles, and liposomes, hold potential for application in food additives and intelligent drug delivery due to availability, biocompatibility, and controllable degradability.^154^ Additionally, while the mainstream research direction focuses on the aforementioned topics, shifting the focus to other biologically active research areas such as anti-diabetes, anti-coagulation, and neuroprotection, could prove worthwhile, as the studies in these areas are still relatively scarce. This could further broaden the development prospects of 18β-GA derivatives and increase their role in various fields.

## Conclusions

In conclusion, the past decade has yielded promising research on the therapeutic potential of 18β-GA and its derivatives for various diseases, including cancer, inflammation, bacterial infection, hepatic diseases, and viral infections. Pharmacological effects have been observed through a variety of pathways, including inflammation-related signaling, immune response modulation, and gene expression regulation. However, it is unfortunate that no derivatives have entered clinical trials (from https://www.clinicaltrials.gov) due to their poor pharmacological properties, low bioavailability, significant toxic side effects, and other factors.

The review of over 200 chemical structures and key activity data in this review article serves as a valuable data resource for pharmaceutical chemists and also provides future research directions. Future research, except self-assembling derivatives, as well as exploring other related fields should more focus on revealing the mechanisms of action or the target protein and the relationship with the SAR of derivates and to further guide the structural modifications. With further research and optimization, 18β-GA derivatives will address the above crucial issues that hold great promise as potential therapeutic agents for various diseases.

## Conflicts of interest

There are no conflicts to declare.

## Supplementary Material
